# A review on green synthesis of silver nanoparticles (SNPs) using plant extracts: a multifaceted approach in photocatalysis, environmental remediation, and biomedicine

**DOI:** 10.1039/d4ra07519f

**Published:** 2025-02-06

**Authors:** Sehar Shahzadi, Sehrish Fatima, Qurat ul ain, Zunaira Shafiq, Muhammad Ramzan Saeed Ashraf Janjua

**Affiliations:** a Department of Chemistry, Government College University Faisalabad Faisalabad 38000 Pakistan Dr_Janjua2010@yahoo.com Janjua@gcuf.edu.pk +92 300 660 4948

## Abstract

A sustainable and viable alternative for conventional chemical and physical approaches is the green production of silver nanoparticles (SNPs) using plant extracts. This review centers on the diverse applications of plant-mediated SNPs in biomedicine, environmental remediation, and photocatalysis. *Ocimum sanctum* (tulsi), *Curcuma longa* (turmeric), and *Azadirachta indica* (neem) and many others are plant extracts that have been used as stabilizing and reducing agents because of their extensive phytochemical profiles. The resulting SNPs have outstanding qualities, such as better photocatalytic degradation of organic dyes like methylene blue, antibacterial efficacy towards multidrug-resistant pathogens, biocompatibility for possible therapeutic applications, and regulated magnitude (10–50 nm), enhanced rigidity, and tunable surface plasmon resonance. Significant effects of plant extract type, amount, and synthesis parameters on the physical and functional characteristics of SNPs are revealed by key findings. Along with highlighting important issues and potential paths forward, this review also underlines the necessity of scalable production, thorough toxicity evaluations, and investigating the incorporation of SNPs into commercial applications. This work highlights how plant-based SNPs can be used to address global environmental and biological concerns by straddling the division between sustainable chemistry and nanotechnology.

## Abstract 2

A sustainable and viable alternative for conventional chemical and physical approaches is the green production of silver nanoparticles (SNPs) using plant extracts.

## Graphical abstract

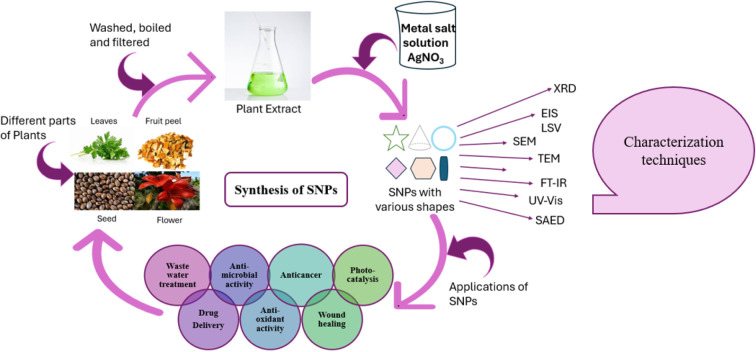

## 1. Introduction

Due to their incredibly small size (measured in nanometers) and elevated surface to volume ratio, which result in both physical and chemical changes in their properties when compared to most materials with the same chemical makeup, NPs are of great interest.^1^ Inorganic NPs include semiconductor NPs (ZnS, CdS, ZnO), metallic NPs (Ag, Au, Al, Cu), and magnetic NPs (Co, Ni, Fe), while organic NPs contain carbon NPs (fullerenes).^2^ Due to their ecological compatibility, which is attributed to the value of materials produced from the environment. Over the past few decades, green metallic NP-centered goods have become increasingly accepted and popular in research. Given the wealth of resources found in the biosphere that fit into this category, a wide range of goods would always be available to fulfil the need for green NT.^3^ Plant components like leaves, pods, stem bark, roots, fruits, and others have long been used in the environment friendly synthesis of metallic NPs, including titanium dioxide, gold, copper, zinc, platinum, and silver.^4,5^ Plants have found use in the manufacture of metal NPs, specifically in the field of nanoparticle synthesis involving living organisms.^6^ Compared to other biological methods that are safe for the environment, using plants to synthesize NPs could be advantageous because it reduces the complex procedure of preserving cell cultures. It would be more beneficial for biosynthetic processes to make NPs extracellularly from plants or their extracts in a regulated way that considers their magnitude, dispersity, and form.^7^

One form of nanomaterial with several uses in food packaging, industrial operations, medicine, and water treatment is SNPs.^8^ Their remarkable electrical, thermal, optical, and biological characteristics which also bear similarities to those of noble metals like copper and gold—set them apart from other metal ions.^9^ SNPs are a particular kind of 0D material that has distinct morphologies and diameters ranging from 1 to 100 nm.^10^ According to the periodic chart, noble metals include silver, copper, and gold.^11^ Thus, noble metals produced metallic NPs that have drawn numerous consideration as a result of their unique biological, chemical and physical features contrasted to other metals.^12^ Interest in the biological assessment of SNPs in everyday human life was first aroused by an unusual antibacterial characteristic of the locus. One of the most important factors is particle size since it establishes the basic characteristics of the substance.^13^ For example, it has been reported that size affects a number of important features and functions in biological systems, such as drug transport, distribution, and targeting. The SNPs antibacterial activity is more potent the smaller the silver nuclei. Thus, size management and size distribution are essential characteristics of higher-performing end goods. Changing the stabilizers and reducing agents during preparation is a common technique to regulate the size and size distribution of SNPs.^14^

Research has demonstrated that phenolics, flavonoids, and glycosides, among other various bio-active metabolites, are abundant in natural goods and can easily reduce metallic ions when combined in the similar reaction vessel.^15–18^ This category comprises polymers such as 3-amonibenzene boronic acid groups, polyethylene glycol, polyvinylpyrrolidone, polyvinyl alcohol, and pluronic groups.^19,20^ NPs can be produced using a number of well-established techniques, such as chemical, physical, and green synthesis methods. High transparency, controlled structure, and high revenue are the main advantages of physical synthesis, which includes vapor deposition, microwave irradiation, pulsed laser, sonochemical reduction, gamma radiation, and plasma chemical.^21^ Another common method is chemical synthesis, which generates the use of heat breakdown, electrochemical synthesis, microemulsions, and lowering agents in polyol. Both physical and chemical preparation techniques, however, have drawbacks, such as the requirement for premium materials, exacting procedures, large budgets, and possible biological toxicity because of hazardous residues.^22^ Green synthesis techniques, on the other hand, provide a biocompatible and sustainable substitute by using naturally occurring reducing agents derived from yeast, fungi, bacteria, and plant extracts that are not poisonous or pathogenic. Green synthesis benefits both the environment and technology since it reduces the need for dangerous chemicals and unfavorable synthetic conditions that are frequently used in the production of NPs.^23,24^

The goal of this study is to present a thorough background of the green synthesis, characteristics, characterization methods, and uses of SNPs in biological, industrial, environmental, biosensing, and photocatalytic fields. It also emphasizes new developments and viewpoints in this highly competitive field. The sources included in this review were chosen for their topical relevance, recent publications (mostly from the last ten years), and keywords related to the characterization, antibacterial, industrial, biological, environmental, and biosensor uses of SNPs as well as green synthesis.

## 2. Principle of green synthesis

“Green Chemistry” in relation to “Sustainable Development” has been extensively researched for fewer than 15 years globally. One definition of sustainable development is development that addresses current demands while maintaining the capacity of future generations to meet their own needs. Since sustainable development is concerned with proof of pollution and the excessive usage of natural resources, it is particularly significant for companies dependent on chemistry. For a long time, chemistry has been perceived as a dangerous subject, and people often equate the name “chemical” with hazard and toxicity.^25^

In general, there are numerous ways to reduce risk by using what is known as protective gear; nevertheless, the danger of hazards and exposure rises when safety precautions fail. When there are significant risks and inadequate exposure, the results can be catastrophic, leading to harm or even death. Thus, minimizing intrinsic dangers and lowering the risk of accident and damage are necessary when designing safe, sustainable chemicals and processes.^26^

### 2.1 Green synthesis of SNPs

**Fig. 1:**
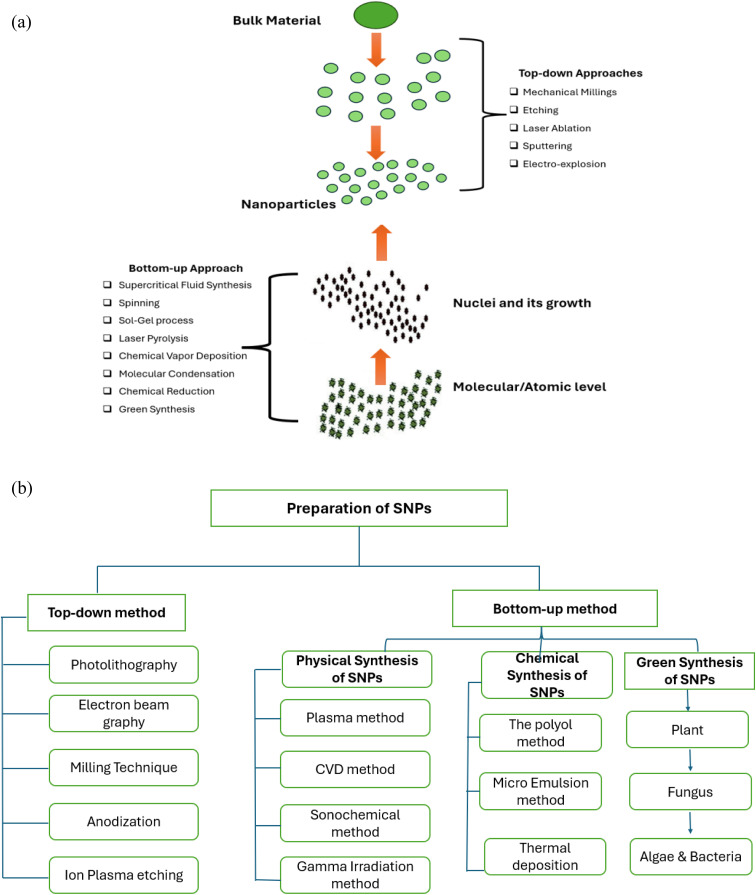
(a) Methods for producing NPs.^29^ (b) Enlisting the conventional and green synthesis methods of SNPs (reconstructed and modified from ref. 30).

The choice of a safe stabilizing substance, an effective reducing agent, and an environment friendly solvent are the 3 crucial requirements for the preparation of NPs. Many synthetic techniques, including physical, chemical, and biosynthetic processes, have been used to synthesize NPs. The chemical methods that are usually used are very costly and include the usage of risky and deadly materials that present several environmental risks.^27^ The biosynthetic way is a green, unharmed, and ecologically acceptable way to create NPs for use in biomedical applications using microbes and plants. Among other things, algae, fungi, bacteria, and plants can be employed to carry out this synthesis. Many NPs have been synthesized from plant parts such as leaves, fruits, roots, stems, and seeds because these plant parts include phytochemicals that function as stabilizing and reducing agents in the extract. Numerous biological and physicochemical processes for the creation of NPs can be divided into two distinct classes: top–down and bottom–up.^28^Fig. 1a and b illustrates the development of NPs using several biological and physicochemical methods.

Fig. 1a shows the top–down and bottom–up methods for creating nanomaterials. The top–down approach is predicated on the idea of using chemical or mechanical interventions to reduce large-size materials to nano-size. Unlike the physical top–down strategy, which is based on chemical reactions, the bottom–up approach creates atoms or molecules through a variety of chemical reactions. Some other conventional methods (physical and chemical) along with green synthesis method are shown in Fig. 1b.^31^

#### 2.1.1 Stabilizer's and caping agent role in green synthesis of SNPs

SNPs may now be produced sustainably and environmentally by green synthesis, which replaces traditional chemical and physical processes. This method does not use harmful reducing chemicals or stabilizers because it uses biological organisms including fungi, algae plants, and bacteria to convert Ag ions into NPs. The rich source of secondary metabolites that are particularly favored are plant extracts, which serve as both capping and lowering agents.^13^ For example, it has been observed that the utilization of *Azadirachta indica* and *Eucalyptus globulus* plant extract results in SNPs with enhanced antibacterial capabilities as well as controlled size and form.^14^ Furthermore, *Syzygium jambos*,^32^*Coriandrum sativum*,^33^*Prunus yedoensis*,^34^*Bunium persicum*,^35^*Vigna radiate*,^36^*Adenium obesum*,^37^ and *Microsorum pteropus*^38^*Lantana camara*,^39^ have all shown encouraging outcomes. In the same way, *Aspergillus niger* and other fungi have been employed to synthesize SNPs that indicate the function of fungal proteins in the maintenance route.^37,40^ Additionally, the algal-mediated production shows promise; in mild conditions, *Chlorella vulgaris* (algae like) can aid in the production of SNPs.^32,33^

These characteristics make green synthesis a desirable choice for environmental and medical applications. The preparation of SNPs using green procedures is dependent on a number of aspects, including the amount of Ag ions, pH, the type of biological material, temperature, and reaction time. For example, the type of plant extract utilized can have a significant impact on the size and structure of the created NPs as described in Table 3. According to research by Singh *et al.* and Dutta *et al.* the aqueous extract of Parsley leaves generated sphere-shaped SNPs with a standard range of 20–30 nm.^32,41^

Temperature and pH are examples of reaction parameters that might be quite significant. It has been discovered that in neutral pH conditions, increasing the reaction's temperature encourages the formation of smaller NPs.^42^ The reduction and stabilization processes are further aided by the presence of particular phytochemicals in the plant extracts, such as alkaloids, flavonoids and terpenoids. For example, employing extract from *Camellia sinensis* (green tea),^43^ flavonoids have been found to be important reducing agents in the production of SNPs.^44^ The green synthesis approach improves the biological activity of the NPs while simultaneously lessening its negative effects on the environment.

#### 2.1.2 Reducing agents

**Fig. 2:**
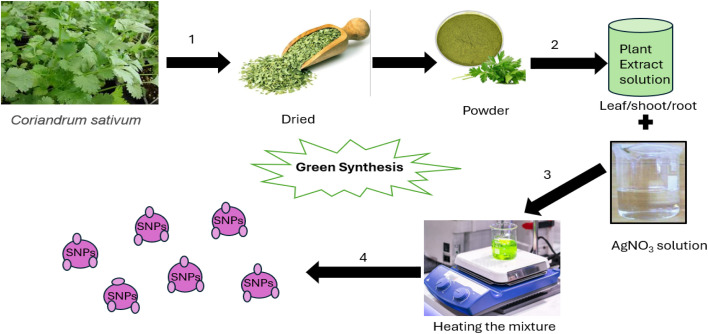
Preparation method of synthesizing SNPs using *Coriandrum sativum* extract.

**Fig. 3:**
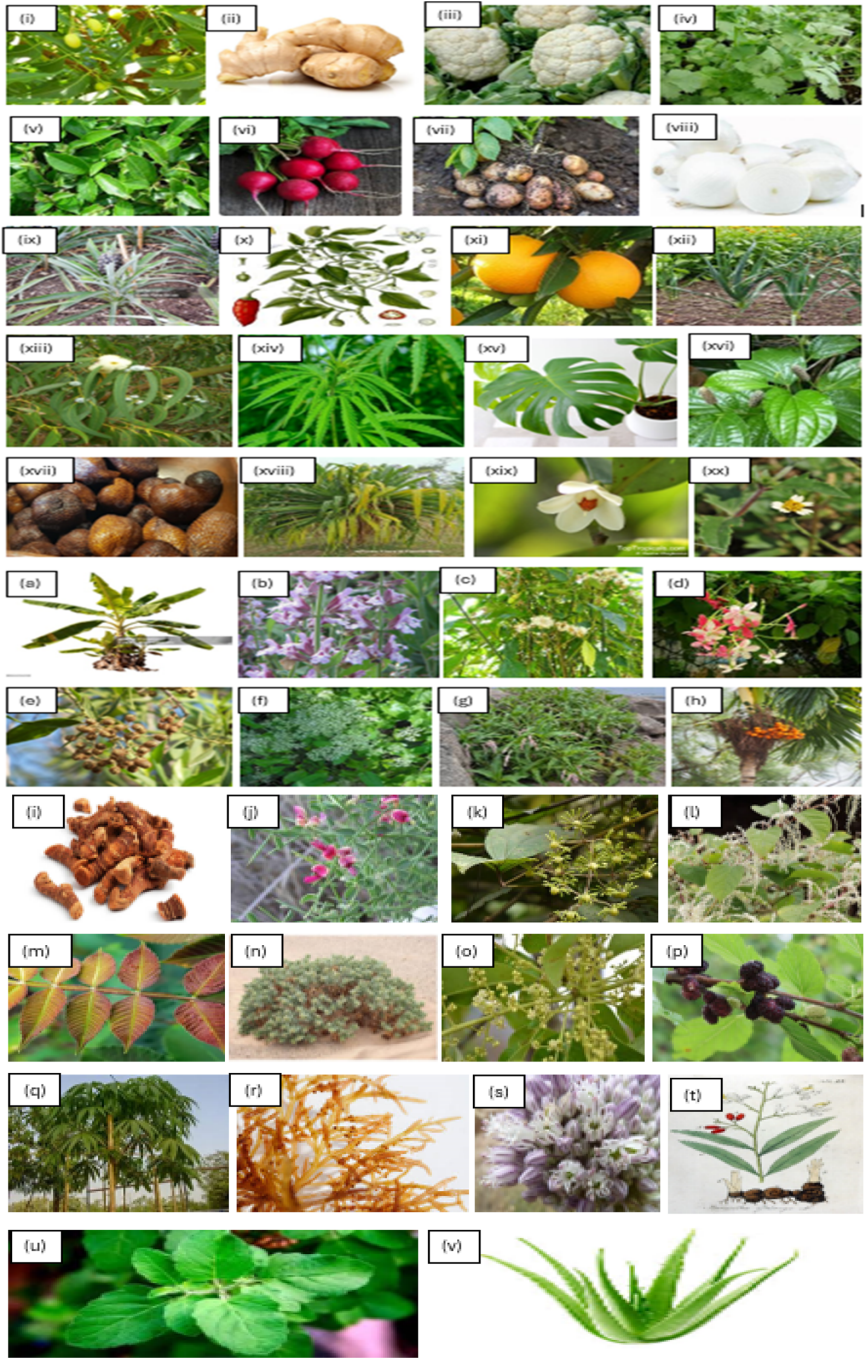
Synthesis of Ag NPs using plant extracts: (i) *Azadirachta indica* (neem)^14,44,45^ (ii) *Zingiber officinale* (ginger)^46,47^ (iii) cauliflower (*Brassica oleracea*)^48^ (iv) *Coriandrum sativum* (parsley)^49,50^ (v) *Camellia sinensis* (green tea)^43,51,52^ (vi) *Beta vulgaris* (radish)^53,54^ (vii) *Solanum tuberosum* (potato)^55^ (viii) *Allium cepa* (white onion)^56^ (ix) *Ananas comosus* (pineapple)^57^ (x) *Capsicum annuum* (red pepper)^58^ (xi) *Citrus sinensis*^59^ (xii) *Pandanus atrocarpus*^60^ (xiii) *Eucalyptus globulus*^14^ (xiv) *Cannabis sativa*^61^ (xv) *Monstera deliciosa*^62^ (xvi) *Piper chaba*^63^ (xvii) *Salacca zalacca* (snake fruit)^64^ (xviii) *Pandanus tectorius*^65^ (xix) *Adinandra poilanei*^66^ (xx) *Tridax procumbens*^67^ (a) *Musa acuminata* (banana peel)^68^ (b) *Salvia officinalis*^69^ (c)*Vernonia amygdalina*^70^ (d) *Rangoon creeper*^71^ (e) *Conocarpus lancifolius*^72^ (f) *Mikania cordata*^73^(g) *Persicaria senegalensis*^74^ (h) *Areca catechu*^66^ (i) *Curcuma longa*^75^ (j) *Alhagi graecorum*^76^ (k) *Nervalia zeylanica*^77^ (l) *Reynoutria bohemica*^78^ (m) *Rhus chinensis* mill^79^ (n) *Rhazya stricta*^80^ (o) *Buchanania lanzan spreng*^81^ (p) *Morus rubra* (mulberry)^82^ (q) *Bombax ceiba*^83^ (r) *Champia parvula*^84^ (s) *Allium ampeloprasum*^85^ (t) *Alpinia galanga*^86^ (u) *Ocimum sanctum* (tulsi)^87–89^ (v) *Aloe barbadensis* miller (*Aloe vera*).^90,91^

Research has demonstrated that SNPs produced with environment friendly methods have strong antimicrobial, antifungal, and anticancer properties, which makes them appropriate for a variety of medicinal uses.^44^Fig. 2 shows the vivid representation of the synthesis manner, emphasizing important stages like the withdrawal of bioactive complexes from coriandrum and their contact with Ag ions, which alleviate the reduced Ag ions to form silver NPs, prevent accumulation, and yet bring about the formation of standardized particle dimension. Fig. 3 explained that the method involves multiple steps: (1) gathering fresh leaves, washing them well, and allowing them to dry at room *T* to obtain the plant, algae, or fungal extract; (2) making a fine powder out of the dried leaves and mixing it with distilled water to create an aqueous extract; and (3) heating the blend while continuously moving it to assurance the withdrawal of bioactive constituents. Strain the extract to eliminate any solids and leave behind a pure solution when it has been heated, generating an AgNO_3_ solution at a specific proportions. Next, stir the plant extract and water together in a 1 : 1 ratio while the liquid is still at room temperature. The reaction lasted for a few hours to ensure complete Ag ion reduction. (4) To obtain the final product, centrifuge the synthesized SNPs, rinse them in deionized water to remove any remaining Ag ions or nonreactive plant extract, and then dry them.^44^ Factors that affect the green synthesis methods are discussed in Table 1. From the latest literature review, SNPs can be produced from different plant extracts (as shown in Fig. 3) and their diameter and shape from each plant extract obtained are discussed in Table 3.

**Table 1:** Factors that affect the green synthesis methods^30,92,93^

Factors	Description	Impact on NPs synthesis	Ref.
pH	How acidic or alkaline the reaction media is	pH has an impact on the nucleation and growing processes of NPs by changing the charge on their surfaces. Smaller particles are often produced by higher pH, but aggregation can occur at lower pH	94
Temperature	The temperature during the process of synthesis	Greater heat can cause aggregation; lower temperatures promote nucleation and reduction rates, resulting in lesser and more homogeneous NPs	95
Concentration	The quantity of reducing agent and metal precursor	Reducing agent amount impacts stability and reduction rate; higher precursor amounts enhance nucleation sites, producing reduced atoms but can also promote accumulation	49
Time	Time periods of reaction process	Affects the development and maturation of NPs	30
		Larger, more definite shapes might result from longer durations, but too much time can also lead to aggregation and polydispersity	
Light intensity	Crucial factor that significantly impacts the synthesis of SNPs	Since UV light gives off energy that accelerates the reduction of silver ions, its function is very noteworthy	96

## 3. Properties of SNPs

Having a thorough understanding of SNPs' characteristics is essential to maximizing their possible applications. For the purpose of determining how SNPs affect the environment, the benefits and drawbacks of employing them must be precisely measured. Research and exploration into the potential advantages of SNPs in various applications have been conducted extensively. Adverse effects of SNPs have also been studied by numerous studies. SNPs are discussed in this section along with their morphologies, sizes, crystalline structures, toxicity, and optical, electrical, and catalytic capabilities.

### 3.1 Structure and size of SNPs

SNP sizes and forms are highly dependent on the solution's concentration. For example, their size and distribution change in proportion to the quantity of precursor metal salts and polysaccharides. The investigation discovered that raising the silver nitrate amount from 2.5 to 15 mM produced bigger particles and Ag clusters. With a higher polysaccharide concentration, larger spherical SNPs were produced.^97^ Raza *et al.* produced SNPs in a variety of sizes and morphologies by employing a variety of reducing and capping agents. According to their research, spherical SNPs with diameters of 15 and 43 nm can be created by 1 weight percent decrease of an aqueous starch solution of silver nitrate consuming NaOH and dextrose glucose at of 70 °C for 30 minutes.^98^

Reducing agent impact on NP ranges at room *T* was also examined in their investigation. It was discovered that when the reducing agent was applied to the Ag salt in liquid crystalline pluronic P-123 and L-64, spherical Ag particles with a diameter of 8 and 24 nm were produced. In a separate procedure, when the silver nitrate precursor salt was reduced by NaOH in a combination of polyvinyl pyrrolidone (0.053 g) and ethylene glycol (11 g), SNPs were produced in the form of substantial 520 nm self-assembled cubes. Scientists concluded that SNPs may be synthesized into a range of shapes, including as mixed geometries, pure cubes, and stars, by using the capping chemicals PVP and poly(methyl vinyl ether) as shape-modifying agents. Conversely, spherical SNPs were produced by employing NaOH and D(+) glucose as reducing agents in a water-soluble environment.^99^

Commonly the shape of SNPs has a significant impact on their characteristics. The result of intricate combinations of surface, crystalline, and molecular characteristics is morphological transformation. Numerous size- and shape-controlled synthesis techniques for SNPs have been suggested and improved in order to optimize their characteristics. We now have a good understanding of the connections between their morphology—such as size and shape—and certain attributes. For instance, the high surface-to-volume ratio of isotropic geometry—such as that of a spherical form—compared to anisotropic geometry was responsible for the higher antibacterial activity.^100^

Smaller particles with a bigger surface area work better as antibacterials than larger ones because SNPs can contact bacterial cells. In terms of plasmonic characteristics, larger particles have a broader UV-vis absorption spectrum than smaller ones. Furthermore, the sizes of their NPs have an impact on their thermodynamic properties. For example, because of its surface free energy, bulk silver has a lower molar heat capacity than SNPs. Furthermore, it has been demonstrated that NPs have a higher molar entropy than bulk silver. The entropy connected to the initial derivatives of Gibbs free energy is the cause of this. According to a review research, SNPs with common diameters in general applications fall between 1 and 10 nm. SNPs quantum confinement and surface area-to-volume ratio may be impacted by its size. SNP size may potentially have an impact on the presence of bacteria or viruses. SNPs between 1 and 10 nm, for example, have been demonstrated *in vitro* to interact with the HIV-1 virus *via* binding to the host cells. Furthermore, when compared to spherical and rod-shaped NPs, the truncated triangular NPs showed the highest biocidal effect against the Gram-negative bacterium *E. coli*.^101^

### 3.2 Toxic behaviour

The exceptional chemical characteristics of SNPs make them useful for a broad extent of purposes. Reports have shown that SNPs are especially efficient antimicrobials against viruses, eukaryotic and bacteria pathogens. SNPs have been linked to certain commercial products, such as feminine hygiene products and contraceptive devices, which may pose a risk to human reproductive health.^102^ SNPs have been linked to certain commercial products, such as feminine hygiene products and contraceptive devices, which may pose a risk to human reproductive health. The researchers reviewed the antibacterial activity of SNPs against *E. coli*. SNPs were used in the study at different doses ranging from 10 to 100 μg cm^−3^. Due to the widespread utilization of these materials in several fabrics and cosmetics, the number of dangerous SNPs that are exposed to human skin when using consumer goods may increase. Depending on the aggregate of silver coating, fabric quality, pH, and perspiration formation, SNPs may be released from consumer products. Utilizing fake individual covering, it was found that SNPs were secreted from antibacterial fabric goods into perspiration. In a different findings, SNPs used an *in vitro* method to cause skin cancer and human fibrosarcoma cells to undergo oxidative stress and death.^103^

SNPs can also have a variety of detrimental effects, as numerous studies have shown. These effects include an increase in the oxidative stress and cytotoxicity of human hepatoma HepG2 cells, a decrease in the chemotaxis and proliferation of human mesenchymal stem cells, and more. As discussed before, the toxicity of SNPs depends significantly on their doses and sizes.^104^ While there is ongoing discussion over their toxicity mechanisms, other potential pathways have been proposed. For example, structural alterations and eventual damage were brought about by the interaction of SNPs with bacterial membrane constituents, which ultimately resulted in bacterial cell death. On the other hand, SNPs cause cell harm by blocking the respiratory enzyme of bacteria and promoting the production of reactive oxygen. Furthermore, it's also feasible that the chemical changes that the NPs undergo during their intracellular operations is the driving force that causes the toxicity mechanisms. By employing this method, the chemical alteration of SNPs allowed for the successful capture of their 3D dispersion within a single human monocyte. According to the relevant study, elemental silver is converted to Ag^+^ ions and eventually Ag–S species, which are the principal processes indicating particulate silver's harmfulness.^105^

### 3.3 Polycrystalline structures

XRD patterns can be used to reveal the crystalline configuration of SNPs. According to a number of studies, SNPs have a cubic structure, with peaks located at 38.06∼, 44.22∼, 64.48∼, and 77.32∼, respectively. These peaks correspond to the dispersing angle 2*θ* from the (1 1 1), (2 2 0), (2 0 0), and (3 1 1) planes. Furthermore, SNPs exhibit a diffraction pattern at 38.5, 44, and 64.5 (2*θ*). The fcc silver's (111), (200), and (220) planes can be indexed to these patterns.^8^

### 3.4 Qualities of optics

Numerous studies have demonstrated that SNPs use a process called the stimulation of localized surface plasmon resonance to grasp EM radiation in the range of 380 to 450 nm in visible region.^106^ It was discovered that the spherical SNPs produced by glucose reduction exhibited Surface Plasma Resonance (SPR) at 400 nm. Furthermore, for the same structure of SNPs formed following NaOH reduction, their analysis found that the NPs absorbed the highest EM radiation at 420 nm. Instead, the scientists illustrated that SNPs synthesized in various proportions using gallic acid utilizing an aqueous chemical reduction method.^107^

It was shown that spherical SNPs with a diameter of 7 nm have an SPR at 410 nm, whereas those with a diameter of 29 nm have a resonance at 425 nm. Additionally, a broader band with an extreme at 490 nm was displayed by SNPs with proportions of 89 nm. The width of SPR was found to be correlated with the size distributions of the NPs. SNPs with irregular shapes may exhibit two or more plasmon resonances, contingent upon the proportion of the nanoparticle. The results presented above indicate that sensor devices may be able to make use of SNPs. Their special qualities have recently been exploited to their advantage as sensors for the sensitive colorimetric detection of Cr in vegetable samples, industrialized wastes, and surface waterways.^108^ Additionally, the essential micelle concentration of cationic surfactants was found using their superior qualities. Furthermore, it was discovered that their antibacterial activity was dependent on surface plasmon resonances. Furthermore, an SNP-based sensor opens the door to ultrasensitive bio-detection studies using incredibly straightforward, compact, lightweight, durable, and affordable equipment. It has been shown that SNPs increase the signal strength of surface-enhanced Raman scattering (SERS) filter paper. As an alternative, they can be applied to enhance solar cell performance (Table 2).^97^

**Table 2:** Summary of physical properties of some plants^109,110^

Plant source	Size	Shape	Optical properties	Toxicity
*Azadirachta indica* (neem)	10–30 nm	Spherical	Sharp SPR peak around 400–420 nm, characteristic of smaller nanoparticles	Low toxicity towards mammalian cells; eco-friendly. Antibacterial activity reduces microbial toxicity
*Camellia sinensis* (green tea)	10–15 nm	Spherical to slightly oval	Strong SPR peak around 420 nm, indicating small and uniform size	Low toxicity is considered biocompatible. Suitable for biomedical applications
*Ocimum sanctum* (tulsi)	20–50 nm	Spherical	Moderate SPR peaks around 420–440 nm. Slight red-shift with larger size	Low toxicity, no significant adverse effects on human cells
*Zingiber officinale* (ginger)	30–50 nm	Spherical to slightly oval	SPR peak around 420–430 nm with good intensity	Low toxicity; non-toxic to humans with potential for biomedical applications
*Aloe vera*	20–50 nm	Spherical	SPR peak at around 430 nm, moderate intensity	Biocompatible, very low toxicity, suitable for cosmetics and biomedical uses

**Table 3:** Synthesis of SNPs using variety of plant's extract and their morphology

Plant name	Plant part	Diameter of NPs (nm)	Type of NPs	Shapes of NPs	Ref.
*Zingiber officinale* (ginger)	Rhizome	28–105	Ag	Spherical	46 and 111
Cauliflower (*Brassica oleracea*)	White flower	25–100	Ag	Globular	48 and 112
*Coriandrum sativum* (parsley)	Fresh leaves	8–75	Ag	Spherical	33, 49 and 50
*Camellia sinensis* (green tea)	Dried leaves	77.4	Ag	Spherical	32, 43, 51, 52 and 113
*Beta vulgaris* (radish)	Root	15	Ag	Spherical	53 and 54
*Solanum tuberosum* (potato)	Potato tuber	20	Ag	Spherical	55 and 92
*Allium cepa* (white onion)	Inner layer	10	Ag	Spherical	47 and 56
*Ananas comosus* (pineapple)	Leave	40–150	Ag	Hexagonal spherical shape	57 and 114
*Capsicum annuum* (red pepper)	Fresh leaf	19	Ag	Spherical	58
*Citrus sinensis*	Fruit peel	25	Ag	Spherical	59
*Pandanus atrocarpus*	Leaves	14	Ag	Spherical	60 and 115
*Eucalyptus globulus*	Leaves	34.21	Ag	Spherical	14 and 116
*Cannabis sativa*	Seed	43	Ag	Triangular and quasi-spherical	61 and 117
*Monstera deliciosa*	Leaf	Spherical NPs = 25–78	Ag	Spherical	62 and 118
		Small = 25–40			
		Large NPs = 40–78			
*Piper chaba*	Stem	19	Ag	Face centered cubic and spherical	63
*Salacca zalacca* (snake fruit)	Fruit peel	10–50	Ag	Spherical	64
*Pandanus tectorius*	Aerial roots	10–20	Ag	Spherical	119–121
*Adinandra poilanei*	Leaves and twigs	12–20	Ag	Spherical	66 and 122
*Tridax procumbents*	Leaves	11.1–45.4	Ag	Spherical	67
*Musa acuminata* (banana peel)	Banana peel	10–20	Ag	Spherical	123–125
*Salvia officinalis*	Leaf	41	Ag	Spherical	69, 126 and 127
*Rangoon creeper*	Leaves	12	Ag	Oval shaped	71 and 128
*Conocarpus lancifolius*	Leaves	5–30 nm	Ag	Spherical	7, 72 and 129
*Mikania cordata*	Leaves	26.8–46.0	Ag	Spherical	73 and 130
*Persicaria senegalensis*	Leaves	23–71	Ag	Diverse shapes including spherical	74 and 131
*Areca catechu*	Leaves and twigs	12–20	Ag	Spherical	66 and 132–134
*Curcuma longa*	Leaf	15–40	Ag	Spherical	92 and 135
*Alhagi graecorum*	Leaves	22–36	Ag	Spherical	76 and 136
*Ocimum sanctum* (tulsi)	Fresh leaves and stem	17	Ag	Spherical	87–89
*Nervalia zeylanica*	Leaf	34.2	Ag	Spherical	77
*Reynoutria bohemica*	Leaf	40–50	Ag	Spherical	78
*Rhus chinensis* mill	Root and leaf	54.40–30.89	Ag	Spherical	79
*Rhazya stricta*	Leaves	21–90	Ag	Oval-circular	80
*Buchanania lanzan spreng*	Green leaves	23–62	Ag	Spherical	81
*Morus rubra* (mulberry)	Leaves	15–20	Ag	Spherical	82
*Bombax ceiba*	Stem and flower	19.4–20.6	Ag	Spherical	83 and 137
*Champia parvula*	Seaweeds	79	Ag	Round	84
*Allium ampeloprasum*	Aerial part	8–50	Ag	Spherical	85 and 138
*Alpinia galanga*	Stem	50–90	Ag	Spherical	86
*Azadirachta indica* (neem)	Leaves	30–50	Ag	Spherical	123
*Vernonia amygdalina*	Leaves	10–30	Ag	Spherical	70
*Aloe vera*	Fresh leaves	15	Ag	Spherical	90 and 91

### 3.5 Thermal attributes

A material's thermal behaviour is a crucial factor that is carefully considered throughout production or application. Because of the thermodynamic size effect, metal NPs have an amazing low melting temperature. It was frequently used for a variety of objectives. To investigate the thermal characteristics of SNPs, thermogravimetric analysis or differential scanning calorimeters are frequently used.^107^ The Gibbs–Thomson equation is another method for studying the thermal properties of NPs theoretically. More specifically, a material's melting point is important for a number of uses. Because the surface-to-volume proportion in bulk material is low, surface influences on melting point can be disregarded. On the other hand, the melting point of nanomaterials with a high surface-to-volume ratio varies with their size.^106^ Thermodynamic theory provides an excellent explanation for this behaviour. There have been numerous experiments carried out to support this notion. The melting temperatures of SNPs with proportions ranging from 4 to 50 nm were examined in these investigations. It was discovered that melting happened at lower temperatures as the size of SNPs shrank. The thermal behaviour of SNPs with sizes ranging from 3 to 6 nm was investigated in a distinct study.^108^ It was discovered that SNPs heated to 100 °C did not exhibit any appreciable size changes. Conversely, the samples that were heated to 150 °C had a much higher melting point. Furthermore, at 200 °C, the SNP particle size gradually grew, signifying total melting. Moreover, DSC curves displaying a strong exothermic peak at 150 °C further supported this melting temperature. The average thermal conductivity of bulk silver is 429 W m^−1^ K^−1^. According to researchers, SNPs own a 0.37 W m^−1^ K^−1^ thermal conductivity. The analysis's minimal result suggested that organic surfactants were present and were stabilizing the NPs in the solution by coating them.^139^

### 3.6 Electrical characteristics

SNPs can be used in electronic devices because of their special electrical properties. SNPs produced in glass-ceramic medium and ranging in size from 4 to 12 nm were tested for electrical conductivity. 211 SNP films' DC electrical resistance was assessed between 80 and 300 K in temperature. From 120 to 300 K, it was discovered that the surface resistivity rose linear with temperature. The study's other key discovery was that as SNP size increased, so did the effective Debye temperature.^140^ As an alternative, SNPs were used in electrically conductive adhesives (ECAs) as conductive fillers. The charge on a particle in the suspension is represented by this parameter. It can also be utilized as a predictor of the colloidal system's possible stability. This characteristic can be described using the widely used technique of dynamic light scattering. All of the particles in suspension have a large negative or positive zeta potential, which suggests that there is no inclination for the particles to flocculate and instead, they tend to reject one another.^141^ A low worth for this value, on the other hand, indicates that the elements tend to flocculate. Since for this link, Singh *et al.*^41^ examined the zeta potential of the hexagonal and spherical SNPs.

It was discovered that the potential of hexagonal and spherical SNPs was −15.3 and −5.11 mV, respectively. These results suggest that compared to spherical SNPs, hexagonal SNPs are more stable. In comparison to isotropic NPs, anisotropic NPs contain more edges and a larger surface area. Consequently, the anisotropic NPs exhibit an increased amount of negative charge.^142^

### 3.7 Catalytic features

SNPs have been used as efficient catalytic agents to reduce a variety of dyes, including methyl orange, eosin, yellow-12, methylene blue and Rose Bengal.^141^ It was discovered that SNPs produced by the peach kernel shell approach might act as a catalyst to convert 4-nitrophenol into 4-aminophenol. Without the catalyst, the reduction process might take 200 min. On the other hand, with the catalyst present, the reduction took 105 s to complete, using the ideal settings of 10.0 mg SNPs and 250 mM NaBH_4_.^143^ In contrast, 4-nitrophenol can also be reduced by using resin-Au NPs, gum acacia-Pt NPs, Nipolyvinylamine/SBA-15 composite, SNP-seashell, and Ag/TiO_2_ nanocomposite; the last two methods require 8 hours 20 min, 85 min, 4.5 m, and 2 min to completely reduce the compound.^144^ Among the previously mentioned suggested catalysts, SNPs-peach kernel shell is the most effective in terms of time. Moreover, their NPs were seen to reduce methyl orange more quickly than those of SNP-seashell, Cu NP, mesoporous silica SBA-15, and Ag/TiO_2_ nanocomposite.^145^ Similar outcomes were seen when the NPs were utilized to reduce methylene blue more successfully than SNP-seashell, porous Cu microspheres, and Ag/TiO_2_ nanocomposite.^146^ In the presence of NaBH_4_, the reduction mechanisms of a number of dyes utilizing SNPs have been demonstrated to adhere to the Langmuir–Hinshelwood model. NaBH_4_ modifies the pH of the entire solution by acting as an e^−^ and H donor.^147,148^ Subsequently, the SNP undergoes a positive surface charge change prior to BH_4_^−^, and the dye is simultaneously adsorbed on the SNP surface. Following their receipt from tetrahydridoborate ion, the SNPs transfer the electrons to the dye molecules. Furthermore, the hydrogenation of azo dyes is facilitated by a significant amount of hydrogen provided by NaBH_4_ when SNPs are present.^149^ Additionally, the end product may desorb to a colorless state due to SNPs' enormous surface area.^150^

## 4. Mechanistic pathway of SNPs synthesis *via* plant extracts

Since past few decades, SNPs have emerged as one of the most widely studied subjects. Their benefit in many applications is their capacity to build SNPs using various synthetic methods based on the desired properties and applications. The most favorable synthesis method nowadays is green synthesis of SNPs. To develop new techniques for creating NPs, researchers are focusing on the green synthesizing process because it is both economical and eco-friendly. The synthesis of large-scale NPs, which could only be accomplished at the laboratory scale, was done using the green method.^102^ SNPs have found extensive use in food packaging, medical equipment, and other products due to their potent antibacterial action. Gaining a deeper comprehension of SNPs toxicity and probable toxicity mechanisms is becoming essential due to the growing usage of SNPs.^151^ SNPs synthesized by using plants are the easiest to prepare.^152–157^ SNPs need Ag^+^ ion solution and reducing unit. The main challenge is the reduction followed by the stabilization of the Ag^+^ ions. It can only be completed by combining Ag^+^ with other biomolecules like vitamins, amino acids, alkaloids, terpenes and proteins, these are the simplest and cheapest way to synthesize NPs. It is possible to use any plant to prepare SNPs. Plant-based green NPs are also less expected to have serious adverse effects on human beings in comparison to the chemical and physical methods, but these also have widespread applications.

### 4.1 Role of phytochemicals in the Ag ion reduction

Development of metal NPs by a variety of phytochemicals like cellulose, protein, flavonoids, alkaloids, polysaccharides, along with some other secondary metabolites,^158^ the modest and cost-effective approach to synthesize NPs. The amount of the reducing agent utilized in the extraction process determines the size of the NPs. Metal ions can be depleted into the metal NPs by the involvement of the hydroxyl group.^159^ Extracts obtained from different plants can serve as stabilizers and reductants in the production of metal NPs. NPs are created when different extracts of plants are combined with the solutions of metal and salt. A color shift brought on by the reaction will indicate the formation of NPs. There is a strong demonstration of the fact that wide range of phytochemicals, including proteins, polysaccharides, phenolic compounds, alkaloids, and flavonoids, can produce metal NPs. SNPs are synthesized *via* biochemical, physical, and organic (biological) processes in nanotechnology.^160^

**Fig. 4:**
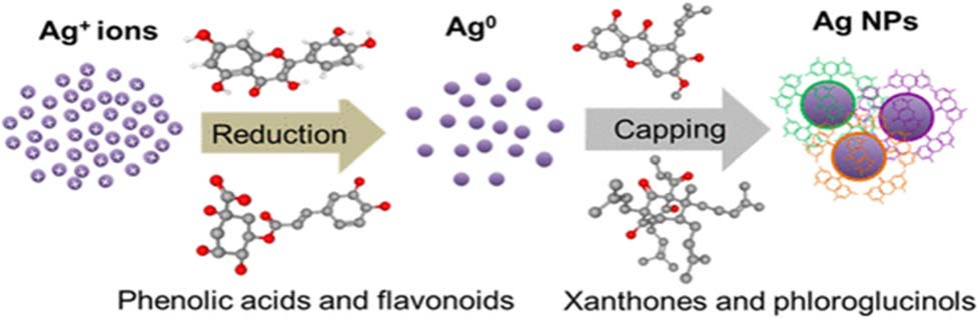
Reduction and capping of SNPs^167^

For metallic NPs, there are two synthetic methods: bottom–up method and top–down method.^161^ Several methods are used in the former one to reduce the magnitude of a suitable bulk material into smaller particles. In the later approach, atoms will self-assay themselves into new nuclei, which subsequently expand and form many nanoscale particles, to generate NPs through chemical and biological means. So, one can produce atomic or molecular nanostructures and controlled synthetic forms of certain nanomaterials by looking at the structures of the reactants and target products. One popular technique is chemical reduction, which converts Ag into SNPs but requires a reductant.^162^ Common reductants such as citrate,^163^ sodium borohydride^164^ and block copolymers^165^ play a significant part in ensuring the stability of the SNPs.^166^ Production of the SNPs is done by first reducing the Ag^+^ ions into the Ag^0^ and then by performing the capping of the reduced Ag^0^. This phenomenon is presented in Fig. 4 given below.

The process of formation of SNPs starts with the generation of Ag atom that will be used as a precursor for the formation of Ag^+^ ions. More and more atoms assemble together and form a cluster that regulates the magnitude and form of SNPs. With its extended applications, the advantages of the chemical reduction process seem to be more obvious. The biggest benefit in this method is that extensive quantity of NPs can be produced without difficulty.^2,168^ However, some drawbacks of using this chemical reduction method for synthesis of SNPs are also there. Reductant, metal precursor, and agents responsible for stabilization or capping, like polyvinylpyrrolidone, are needed to confirm stable chemically synthetized colloids. These kind of chemical waste and substrates are damaging for human beings.^169^ Naturally occurring phytochemical compounds like terpenes, ketones, flavonoids, aldehydes, as well as carboxylic acids are efficient free radical scavengers. They function as stabilizers and potent reductants in the generation of NPs generation.^170^ More studies have revealed that characteristics of the synthesized NPs fluctuate significantly because they are dependent on the part of the plant obtained in the form of extract and then utilized for the synthesis of the SNPs. Saratale *et al.*^171^ used green leaves of *Punica granatum* to make SNPs. Most ideal SNPs are sphere-shaped, with size ranging from 20 nm to 45 nm. Abbasi *et al.*^172^ successfully synthesized SNPs, using bio extract of purple basil.

### 4.2 Mechanistic pathways of SNPs formation

**Fig. 5:**
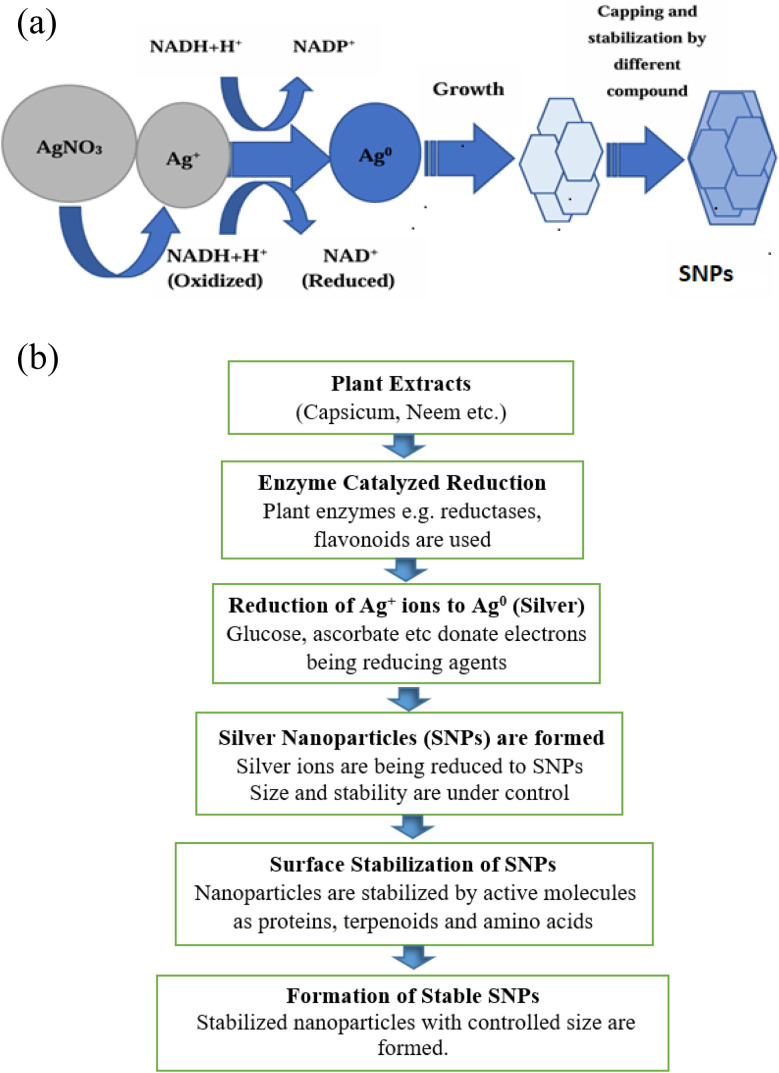
(a) The supposed biosynthesis mechanism of SNPs.^177^ (b) Flowsheet diagram showing mechanistic pathway of SNPs.

There is not any well-explained mechanistic pathway for the synthesis of SNPs. The supposed mechanistic pathway for the production of NPs is an enzyme catalyzed reaction in which complex of enzymes that causes reduction are derived from plant extracts which reduce Ag(NO_3_)_2_ into Ag^+^ and NO_3_^2−^ ions as shown in flowsheet diagram Fig. 5a and b. Composite network of metabolites acting as anti-oxidants along with enzymes of the selected plant work together to inhibit oxidative loss occurring in cellular components. Extract derived from plants encompasses biomolecules containing ketones, alkaloids, β-phenylethylamines ascorbic acid, triterpenes, sterols, polysaccharides, and fructose along with enzymes which can be successfully used as reducing agent to react with Ag^+^ ions, consequently utilized as frameworks to direct the production of SNPs in the solution. Theoretically, biosynthesized cofactors play a crucial part in reducing the corresponding salts to NPs. Though, it looks possible that NPs are synthesized using glucose and ascorbate to reduce AgNO_3_ and HAuCl_4_.^173–176^ Terpenoids act as surface active molecules when neem broth is taken as substrate, which help in stabilization the NPs and reaction is probably facilitated by proteins and reducing sugars containing amino groups, also played dynamic part in the reducing SNPs produced through *Capsicum annuum* extract. There is a change in the secondary structure of proteins that was also formed after reacting with Ag ions.^178,179^

Leaves of *Ficus benghalensis* leaf comprise of large number of antioxidants and polyphenols like flavonoids and quercetin can be used for scavenging molecular species of active oxygen. Hydrogen atoms can donate electrons or hydrogen atoms which help them in their antioxidant action, which will then act as a precursor for changing keto form to enol group. Proteins, phenolic compounds, and other chemicals present in the extract obtained from the leaf of different plants reduce Ag salts and also provide tremendous persistence against accumulation, which can be used to apprehend the mechanistic pathway of development by biological systems.^176,180^

### 4.3 Influence of plant components on the synthesis of SNPs (plant metabolites)

The utilization of bio-mediated routes for SNPs synthesis is appealing due to its ability to produce nontoxic and cost-effective NPs in a single step. Additionally, the production of SNPs can be regulated in size and shape, depending upon the extent of interaction among SNPs and the phytochemical capping agents used in the respected process. The precise interaction between the Ag salts and phytochemicals in the solution that react to form SNPs must be identified and understood. The precise interactions between all phytochemicals have not yet been determined, despite the fact that a widespread phytochemicals, such as amides, flavonoids, and peptides, have been identified as being involved in SNPs biosynthesis.^181^ Alkaloids can operate as reducing agents while terpenoids along with flavonoids mostly act as capping and stabilizing agents, while protein and carbohydrates play its part as reducing as well as stabilization agents throughout the transformation of metallic salts to metallic NPs.

#### 4.3.1 Flavonoids

Plants include well-known secondary metabolites called flavonoids. Plants produce secondary metabolites due to biotic and abiotic stress; flavonoids are also one of them. The synthesis pathways and structural variations of flavonoid are subjected to variation. As a result, leaves, flowers, fruit skin, *etc.* contain them. Among the flavonoids are anthocyanidins, flavanols, flavanones, flavan-3-ols, flavones, and iso-flavones.^182^ Studies have shown that electrons as well as hydrogen-donating ability of the flavonoids make them attractive agent for the synthesis of NPs.

#### 4.3.2 Terpenoids

These are naturally occurring substances that plants release in reaction to biotic and abiotic stressors. The terpene synthase gene is activated by stimulation, which leads to the production of terpenes. Naturally occurring organic molecules, terpenes assemble differently and are generated from five-carbon isoprene units. These types of organisms have the capacity to create both monoterpenoid and sesquiterpenoid. Terpenes in their oxygenated form are called terpenoids. Terpenoids are a class of chemical molecules that are a constituent of the essential oils that plants contain. Out of roughly 3000 recognized essential oils, only 300 are significant from a business standpoint. A basic hydrocarbon molecule called an isoprenoid is frequently present in terpenoids and all other secondary metabolites.^183^ Terpenoids with a lot of potential for depleting Ag^+^ ions into metal oxide NPs include eugenol and methyl chavicol (estragole).^184^

#### 4.3.3 Alkaloids

These find applications in a variety of pharmacological settings. Alkaloids have also been utilized as drugs to treat serious illnesses. One alkaloid that is employed as an insecticide is atropine. Treatment for ovarian cancer involves the use of paclitaxel and its derivative.^185^ Quinine is another kind of alkaloid, it was used in the treatment of the malaria. It can be found in cinchona tree bark.^186^ The production of amino acids such as proline, ornithine, *etc.* is the first step in the synthesis of alkaloids. Afterwards, these amino acids become alkaloids.^187^

### 4.4 Factors affecting synthesis

Certain physiochemical characteristics, including temperature, time, pH, optical, substrate concentration, and enzyme sources, influence the creation of NPs. The information in Table 1 provides the explanation of different factors that affect the synthesis of SNPs.

#### 4.4.1 Effect of temperature

The primary physical factor influencing NP production is temperature. According to reports, Ag(NO_3_)_2_ and starch solution can be autoclaved at 121 °C and 15 psi to create SNPs.^188,189^ Ag and iron NPs were synthesized at standard room temperature utilizing plant's extracts, like aqueous extracts of sorghum bran, according to Njagi *et al.* (2011).^189^ The temperature also affects the additional stability of the NPs; the SNPs produced are kept between 18 and 25 °C for two to three months.^190^

#### 4.4.2 Effect of concentration of the substrate and the reducing agents

SNPs synthesis can be influenced by amount of plant extract and salt concentrations.^191^ Ag nitrate (AgNO_3_) is often employed as a precursor for the production of NPs by using Ag ions. The size and rate of the NPs is greatly influenced by the quantity of salt present in the solution. Larger NPs sizes are frequently the outcome of higher salt concentrations since there are additional Ag ions available for carrying out the reduction. Conversely, low concentration of the salt can produce smaller NPs.^192^ Plant extracts have a wealth of bioactive chemicals, which makes them useful as stabilizing and reducing agents when making NPs.^193^ Increasing the quantity of plant extract can result in larger NPs and a quicker elimination of Ag ions. But excessive concentrations can also lead to inadequate reduction or aggregation, which degrades the NP's quality. The preparation of NPs using chemical methods, such as Tollens' reagent (ammonical Ag nitrate), that causes the reduction of carboxyl group of substrates of sugar like C_6_H_12_O_6_ and ribose, can provide a better understanding of the effect of pH. The size of the NPs is dependent on the ammonium concentration, and this reaction causes the development of a stable complex ion due to ammonia's strong attraction for Ag^+^. Therefore, it is thought that concentration of ammonia and type of reductant used both have a significant influence on the formation of SNPs.^194^

#### 4.4.3 Effect of pH

Another physical element which influences the morphology (size and shape) is pH.^195^ Particle size is influenced by the structural differences between disaccharides and monosaccharides. At pH 11.5, disaccharides in this reaction often yield smaller particles than monosaccharides. Furthermore, compared to particles formed at pH 12.5, those obtained at pH 11.5 were smaller. One way to reduce polydispersity is to lower the reaction medium's pH.

## 5. Types of plants used for synthesis

### 5.1 Examples and case studies

Although the mechanism behind the process of reducing metal ions using green extracts was initially recognized in the early 1900s, it was not fully understood. Subsequently, a variety of metals have been effectively decreased by the use of numerous parts and materials of plants. While throughout the previous 35 years, there has been great attention of scientists towards the biosynthesis of SNPs utilizing extracts from different parts of plants tor even the entire plant.^196^ Temperature, reaction pH, contact time, and the relative quantities of plant extract and metal salt are some of main variables affecting kind, yield, quality, and characteristics of SNPs produced.^197^ SNPs were synthesized utilizing *Z. officinale* and *O. gratissimum*, and UV-vis spectra was examined to verify their identity in detail.^198^ SNPs were bio-synthesized utilizing waste extract from cauliflower and their prospective uses in the light – mediated catalytic degradation of methylene blue dye Hg^2+^ bio sensing were further tested.^199^ The thrombolytic action of *Coriandrum sativum* extracts and Murraya *koenigii* leaf extract carried out manufacturing of SNPs. The aim of the study was to create the SNPs made from *Murraya koenigii* and *Coriandrum sativum* which will have the capacity to lyse clots. GC-MS analysis was performed on the methanolic extract that was extracted from both leaves. After the produced NPs from leaf extracts were analyzed, the standard pattern and peaks were obtained by using XRD technique.^200^ A wide range of plant extracts, including those from lucerne, pine, persimmon, magnolia, platanus, apple, pineapple, and ginkgo, were used extensively in the biosynthesis of SNPs. Similarly, it was discovered that the extract of *Phyllanthus amarus* (stone breaker) leaves was useful in the synthesis of SNPs because of its antibacterial and catalytic qualities. Furthermore, *Beta vulgaris* L.'s aqueous extract showed promise for SNPs biogenesis since it contains pigments, vitamins, manganese, folate, and magnesium in addition to other nutrients that help in the reducing the metal ions to NPs. SNPs biosynthesis is dependent on several important parameters, including as pH, Ag nitrate concentrations, and incubation time.^201^ The aqueous extract of pineapple peel was used to create, describe, and assess SNPs. Colloidal solutions of SNPs exhibited highest absorption at approximately 460 nm following the optimization of SNPs production.^202^*Capsicum annuum*, a chili pepper that is grown all over the world and is highly acknowledged for accumulating large amounts of active chemicals, is a viable option for SNPs biosynthesis. The aggregation of 4.38 mg g_DW_^−1^ of total capsaicinoids, 14.56 mg_GAE_^−1^ g_DW_^−1^ of total phenol containing compounds, 1.67 mg_QE_^−1^ g_DW_^−1^ of total flavonoids, and 1.03 mg_CAE_^−1^ g_DW_^−1^ of total phenolic acids was revealed by phytochemical screening of the aqueous extract of *C. annuum* pericarps. Every identified aromatic compound has a variety of active functional groups that contribute to SNPs production and have a strong potential for antioxidants. As a result, the current study concentrated on the simple, rapid, and efficient process for the bio-synthesis of SNPs, which were then examined for their morphology, including size and form (shape), using scanning UV and FTIR along with some other techniques.^203^ The fabrication of metal NPs using green extracts especially plant extracts has garnered more interest because of its numerous applications, low cost, and less toxic consequences. SNPs were created using an extract from *Eucalyptus globulus*. SNPs formation was verified by observing the color shift from light brown to reddish brown. It was further confirmed by observing the peak of UV-vis spectral lines at 423 nm.^204^ In Indonesia, people eat the pulp of the snake fruit, but discard its peel. In this case, the aqueous extract of snake fruit's phytochemical composition not only aided in reduction process carried out for the creation of SNPs. The phytochemical screening revealed that SNPs were synthesized using snake fruit peel, which contains tannins, alkaloids, saponins, flavonoids and polyphenols.^64^*Salvia officinalis* extract obtained from its leaves was effectively used in the biosynthesis of SNPs. SNPs are created by causing the reduction of metal salts from Ag^+^ to Ag^0^, which releases the abundant phyto-constituents found in extracts of plants, like flavonoids, alkaloids, and terpenoids. It was further verified by FTIR and EDX signals obtained by the observation of their spectra. This method of creating NPs was widely accepted because it is economical, non-toxic, sustainable, and environment friendly.^205^

### 5.2 Phytochemical profile of different plants

The selection of the extract of various species of the plants may also be important because selected plants may have such kind of molecules which can take part in reduction and stabilization of NPs. Different plants, their family name and details about the size and shape of the NPs have been discussed in the table given below (Table 4). A detailed reference to the phytochemicals accountable for the reduction of the Ag salt along with the applications is also debated. The majority of the SNP particles made using plant components produced spherical SNPs, typically measuring between 5 and 85 nm in size.^216^ However, employing *Eclipta prostrata* leaf extract, non-spherical SNPs, in the form of triangles, pentagons and hexagons were also recorded. The reaction took place at room temperature, and the particle size was observed to be varied in between 30-60 nm.^217^ Similarly, the seeds of *Artocarpus heterophyllus* and *Trachyspermum ammi* were used to create both cubic and irregular SNPs.^218^ Reaction times for biosynthesis varied from 10 to 300 minutes at room temperature. One explanation for the Ag precursor's bio-reduction was the high concentration of biomolecules found in the various plant components, including the leaves, seeds, fruits, bark, flowers, and roots. These biomolecules could include a wide variety of biomolecules, including alcohols, alcoholic compounds, alkaloids, alkynes, amino acids, amide, amino acid residues, ascorbic acid, anthraquinones, benzoates, carotenes, carbohydrates, flavonoids, glycosides, leucocyanidin, saponins, proteins, phenolic compounds, steroids, sugars, traces of reducing sugars, triterpenes, and vitamin C.^219^

**Table 4:** Some plants used in the synthesis of SNPs^181^

Sr. no.	Plant	Family	Size of SNPs (nm)	Shape	Phytochemicals required for Ag salt reduction	Applications	Ref.
1	*Alpinia officinarum* (rhizome)	Zingiberaceae	20 to 80	Hexagonal	Amides, polypeptide, carbonyl groups	Photocatalytic degradation of methylene blue	206
2	*Centella asiatica*	Apiaceae	30 to 50	Spherical	Proteins, polyphenols, terpenoid, flavonoids	Catalytic degradation of methyl red, methyl orange	207
3	*Aegle marmelos*	Rutaceae	22.5	Hexagonal, roughly circular, spherical	Phytosterols, flavonoids, and amino acids	Antibacterial activity	208
4	*Bergenia ciliata*	Saxifragaceae	25 to 73	Spherical	Flavonoids, amino acids, and pigments	Antibacterial activity	209
5	*Gracilaria birdiae*	Gracilariaceae	20.2 to 94.9	Spherical	Polysaccharide	Antibacterial activity	210
6	*Dunaliella salina*	Dunaliellaceae	15.26	Spherical	Peptide, polyphenolic	Anticancer potential	211
7	*Waltheria americana*	Malvaceae	7 to 24	Rectangular flakes	Alkaloids, anthraquinones, glycosides, phenols, terpenoids	Antibiotic and antimicrobial activity	212
8	*Areca catechu*	Arecaceae	18.2	Spherical	Polyphenols	Catalytic antioxidant activity	213
9	*Delphinium denudatum*	Ranunculaceae	<85	Spherical	Proteins, terpenoids, amine, alcohol, ketone, aldehyde and carboxylic acid	Antibacterial and mosquito larvicidal activities	214
10	*Punica granatum* (peel)	Lythraceae	30	Spherical	Hydrolysable tannins, chebulic, ellagitannins, esters, gallic acid, and chebulic acid	Catalytic activity on reduction of methylene blue	215

The bacterial cell death brought about by SNPs piercing the cell wall and triggering the bacterial degradation in cytotoxic assays employing cell lines of humans further confirmed the efficacy of SNPs manufactured utilizing floral extracts as antibacterial agents. Additionally, it was demonstrated that SNPs demonstrated effective catalytic activity by producing active free radicals (˙O_2_^−^, ˙OH, and 
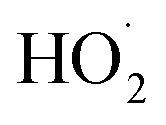
) that could reduce cationic dyes like methylene blue when NaBH_4_ was present.^220^ Additionally, fruit extract (*Lycium barbarum*) mediated SNPs are generated and effectively employed as sensors.^221^ While in 2019 Ameen *et al.*^222^ described the successful synthesis of SNPs using flower extracts of *Mangifera indica*, there was no indication of phytochemical accountable for reduction. Some other scientists mainly Hamedi and Shojaosadati^223^ however, involve a broad screening and characterization of the phytochemicals in case of the synthesis of SNPs.

### 5.3 Bio synthesis using some other parts of plant

Fast biosynthesis of SNPs using plant extracts has been stated by some other plant parts like pericarp extracts of *Sapindus emarginatus*,^224^*Musa* sp. (banana) peel extract *Allium stipitatum* (shallot),^225^ and apricot tree gum,^226^ latex extract of,^227^ inflorescence of *Cocos nucifera*,^215^ and banana peel extract.^228^ The majority of the biosynthesized SNPs were spherical and had particle sizes ranging from 4 to 60 nm, similar to the majority of other plant extract-derived NPs. Alcohol, amines, amide II, aldehydes, carbohydrates, carboxylic acid, alkanes, amino acids, carbonyl compounds, cellulose, ester, hemicelluloses, hydroxyl group, lycopene, pectin, proteins, vitamins (K, C, E), and β-carotene were among the biomolecules found to be involved in bio reduction.^44^

### 5.4 Comparative analysis of different plants in SNPs synthesis

Although the synthesis of SNPs *via* chemical and physical means has been thoroughly investigated, one crucial area of nanotechnology is the development of dependable NPs production technologies.^229^ Synthesis of the NPs by chemical and physical means may have substantial ecological defect, and are usually expensive.^115^ The biological methods, using enzymes and microorganisms, have been suggested as possible eco-friendly substitutes.^173^ Green synthesis of the NPs, which is carried out by using plants or plants extract which aids in reduction of synthesis process, are more advantageous over other biological processes.^230^

Additionally, plant-mediated synthesis of NPs is favored because it is an inexpensive, environment friendly, single-step process, safe for use in human therapy, and can be easily considered for large-scale synthesis. They do away with the complex process of culturing and maintaining the cell. This green synthesis approach appears to be a non-toxic, economical, ecofriendly alternative to the conventional microbiological, chemical and physical methods. It would be suitable for developing an organic process for large-scale production. These SNPs might be applied to lower the microbial burden throughout the waste treatment process.^228^

### 5.5 Gaps and future research in synthetic mechanism

#### 5.5.1 Specific mechanisms of reduction

The precise mechanisms by which phytochemicals reduce silver ions remain unclear. Further research is required for elucidating the molecular interactions between numerous phytochemicals and silver ions. Additionally, the role of specific functional groups in plant metabolites necessitates further investigation.^199^

#### 5.5.2 Size and shape control

Controlling the size, shape, and morphology of silver nanoparticles is vital, yet the underlying mechanisms are not fully implicit. While certain conditions, such as pH, temperature, and concentration, manipulate these characteristics, a more nuanced understanding of their relationship at the molecular level is prerequisite.

#### 5.5.3 Toxicity and biocompatibility

The potential toxicity of silver nanoparticles produced employing plant extracts is an arduous concern. The potential toxicity of silver nanoparticles produced employing plant extracts is an arduous concern. Further investigation is essential to evaluate the environmental and health bearings of these biosynthesized nanoparticles.

#### 5.5.4 Interaction with plant components

The interactions between plant components, such as secondary metabolites, and silver ions are complex and multifaceted. Further exploration is desirable to elucidate how these interactions manipulate the synthesis mechanism, morphology, and stability of nanoparticles.

#### 5.5.5 Scalability

While laboratory-scale synthesis has been considerably investigated, scaling up the green synthesis process for industrial applications establishes significant challenges. Developing standardized protocols and understanding the challenges of large-scale production are indispensable for practical applications which further requires extensive research.

## 6. Characterization of SNPs

Measurement of size, behavior as well as nanostructure of the NPs was made possible with the use of different characterization techniques. There are diverse techniques designed for the analyzation of the NPs. These take into account UV-vis spectroscopy,^231^ SEM, FT-IR, TEM, XRD, and SAED, as some important characterization techniques. Moreover, there are techniques like EIS and Photocurrent measurements that are considered for measuring the performance of Ag@*m*-TiO_2_.

### 6.1 UV-visible spectroscopy

**Fig. 6:**
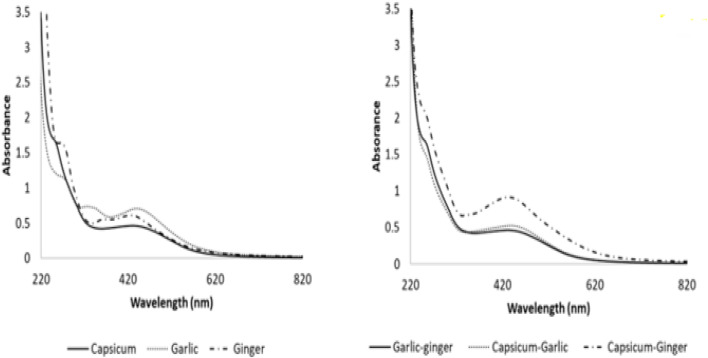
UV-vis spectra of SNPs (left) extracts of pure plants, (right) extracts obtained from mixtures of plant extracts^236^

The modest and potent technique for the determination of characterization of synthesized NPs is the use of UV-vis spectroscopy. SNPs are able to interlink with particular wavelength of light due to their photosensitive characteristics.^232^ A UV-vis spectrophotometer is capable of characterizing variety of NPs morphologies. It is a quick, practical, and careful method for characterizing NPs.^233^ Due to closeness of valence and convection bands, electron mobility is permitted. These free electrons oscillate when exhibited to light waves, which is the reason for the development of SPR. The environment of chemicals and particle size affects the absorption of light by NPs.^234,235^ A detailed spectra of the SNPs prepared by using pure plant extract and mixtures of the plant extracts of capsicum, garlic and ginger has been discussed in Fig. 6 given below. Fig. 6a shows the pure plant extract while Fig. 6b shows the mixture of plant extracts.

UV-vis spectroscopy has shown that SPR peaks at the same wavelength, supporting the constancy of SNPs produced *via* biological processes for over a year. To provide comprehensive information regarding SNPs, UV-vis spectroscopy by itself would be inadequate. Within the reaction media, the UV-vis spectra of SNPs were monitored at 15, 30, 45, and 60 minutes, respectively. The results of the performed research indicated that the SNPs from garlic, ginger, and cayenne pepper caused absorbance peaks extending between 375 nm and 480 nm. A strong resonance was seen at 375 nm, 400 nm, and 480 nm for the first, fifteen, and sixty minutes, respectively.

The SNPs steadily developed throughout the course of the following 24 hours, as indicated by the UV-vis spectra, in less than 60 minutes. The results of the 15 minutes experiment revealed that after a considerable degree of decreasing capacity, the strongest plasmon bands were found at 480 nm in cayenne pepper and the strongest bands between 400 nm and 435 nm in ginger. At 375 nm in spectrum obtained by using UV-vis technique, garlic's SNPs were absorbed. We used absorption spectroscopy to study the optical properties of SNPs. The UV-vis spectra showed a characteristic peak at 440 nm, which verifies the synthesis of SNPs (Fig. 6).^236^

### 6.2 Scanning electron microscopy

This electronic microscopy is the unique process that managed to capture molecules' surface structure. It aids in analyzing the dimensions and dissemination of NPs.^237,238^ When used in combination with SEM, EDX, it provides insight into the sample's constitution.^239^ Using SEM, only the external surface of the sample can be analyzed, excluding internal details, while the degree of purity and the presence of aggregates can be assessed.^240^

**Fig. 7:**
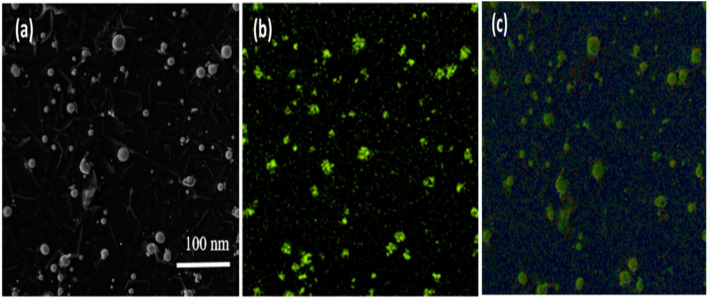
SEM measurements of (a) SNPs with capsicum (b) SNPs with garlic (c) SNPs with ginger.^236^

The composition, shape, and size distribution of SNPs are among those variables that may significantly impact their antibacterial activity. Thus, SEM was used to characterize the form and size of SNPs. The images of SNPs obtained from SEM are displayed in Fig. 7. The measurements of the capsicum (Fig. 7a), garlic (Fig. 7b) and ginger (Fig. 7c) has been determined. According to SEM data, the produced SNPs' size was less than 100 nm.^236^

### 6.3 Transmission electron microscopy

It is a prominent, widely used, and crucial method to calculate the arithmetical values of particle distribution, magnitude, and form. By removing the objective lens from the sample and its image plane, one can calculate the magnification of the TEM.^241^ The method works well for analyzing the volume fraction and the NPs' shape. In addition to offering finer structural resolution, it can be used for additional analytical measurements.^231^ TEM makes use of beam of electron that intermingles with the sample to generate an image on the photographic plate. Individual NPs' chemical and electrical structures can be ascertained using this technique.^242^ Consequently, TEM provides improved resolution and sample information presentation.^243^

**Fig. 8:**
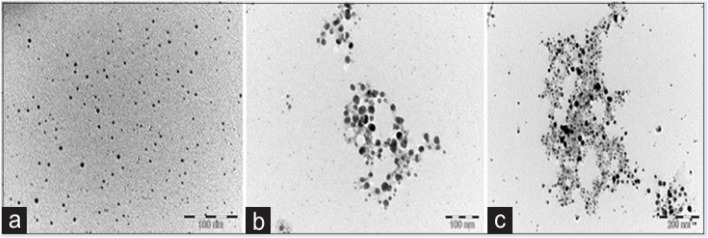
Micrographs of SNPs taken through TEM (a) garlic, (b) ginger, and (c) cayenne pepper.^244^

It is cleared from Fig. 8 given above that Garlic (Fig. 8a), ginger (Fig. 8b), and cayenne pepper (Fig. 8c) all had average diameters of 5.28, 12.97, and 10.86 nm, respectively. Moreover, spherical morphologies with homogeneous particle size distribution have been observed by using TEM imaging of SNPs generated from the spice extracts.

### 6.4 Fourier transform infrared spectroscopy

Physiochemical properties of particles are analyzed using this method to find out the role played by biomolecules in the creation of metallic NPs.^245^ In addition, since the functionally active molecules will be imbedded onto the metal during the catalytic process, FTIR can disclose the information exchange between the substrate and the enzyme.^183,246^ In this technique, an IR beam of radiation enters the taken sample and infiltrates or is absorbed by the remaining portion. The radiation that the sample has absorbed and transmitted can be inferred from the changing spectrum. Less sample heating and quicker data gathering are made possible by FTIR. In light of this, it is thought to be a useful, non-invasive, and practical method for characterizing NPs.

**Fig. 9:**
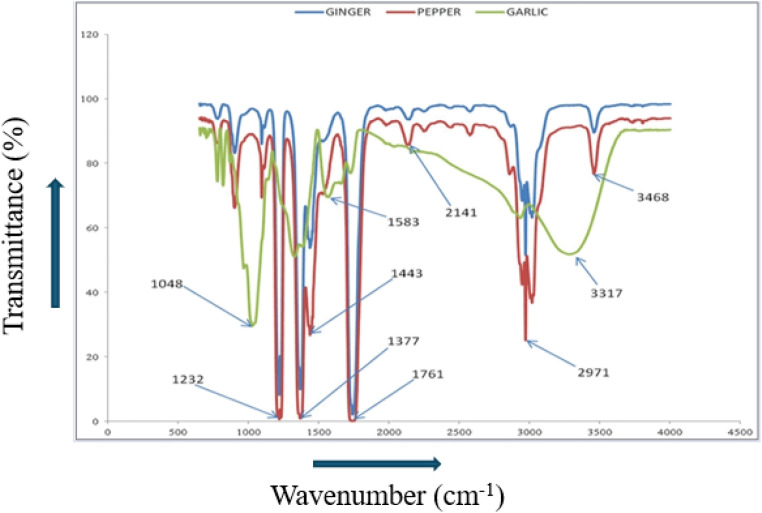
FTIR spectra of SNPs from garlic, ginger, and cayenne pepper^244^

Fig. 9 given above gives detailed information about the FTIR spectra of the SNPs of garlic, ginger and cayenne pepper.

### 6.5 Selected area electron diffraction (SAED)

There are two main reasons why TEM is better than SEM: it can perform more thorough research and has a higher resolution. The high vacuum requirement, small sample size, and labor-intensive sample preparation procedure of TEM are major downsides. One of the convenient technique for visualizing and analyzing the crystalline structure of NPs is SAED (Selected Area Electron Diffraction). Electron scatter-back diffraction studies are commonly carried out in TEMs by means of electrostatic attraction, which accelerates the electrons to the proper frequency and velocity prior to their interaction with the material under examination. After becoming polydisperse, SNPs were mostly spherical, having an average diameter of about 14 nm. ^(106)^

### 6.6 X-ray diffraction

**Fig. 10:**
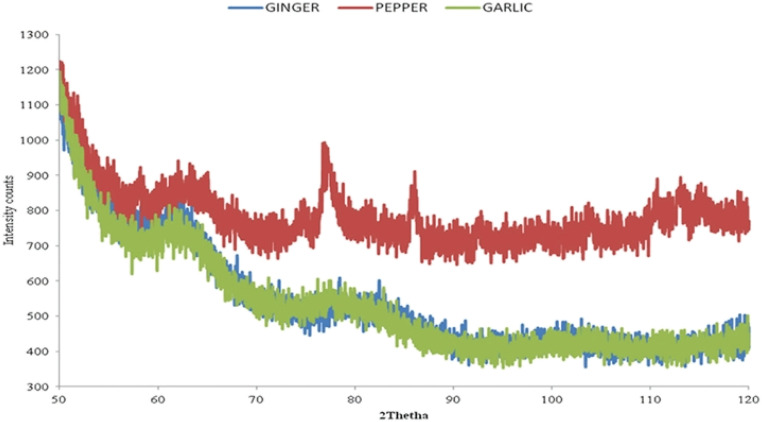
XRD of SNPs obtained by using garlic, ginger, and cayenne pepper^244^

This widely used method for analyzing NPs determine the crystalline formations, statistical complex magnification, various chemical types, the degree of crystallinity, and physical characteristics.^247,248^ Interference between the scattering X-rays was observed by applying Bragg's equation to the polycrystalline material's property.^248^ Thus, a broad variety of substances, such as biomolecules, polymers, super conductors, and so on, that can be investigated using XRD research. The only way to analyze the components stated above is to look for diffraction peaks. The physical and chemical characteristics of crystal molecules can be investigated using this method.^249^ It can be used to examine crystalline materials that are inorganic or inorganic in their nature (Fig. 10).

### 6.7 EIS (electrochemical impedance spectroscopy) measurements

In order to manufacture SNPs, several contemporary methods additionally use titanium oxide nanotubes (TNT) as a base. To recognize the effect of SNPs and flaws in *m*-TiO_2_, measurements of the Ag@*m*-TiO_2_ nanocomposite, EIS and LSV measurements were taken by exposing it to visible light is used in addition to other techniques. It helps in understanding their photoelectrochemical behavior. EIS is a potent method for measuring the separation efficiency of the photo-generated the charge transfer resistance and electron–holes and over the surface of photo-electrodes.^250^ Smaller radius of arc in the EIS plot generally shows lower electron transfer resistance, which usually leads to quick charge shift and more successful segregation.^92^ The Ag@*m*-TiO_2_ photo-electrode had the lowest semicircular arc when compared to the other photo-electrode containing *p*-TiO_2_ and *m*-TiO_2_. This implies faster interfacial charge transfer and better partition of the photo-generated electron–hole pairs under the irradiation of visible light. This implies that the synergistic effects of defects and SPR lead to the highly productive of photo-initiated electrons into holes. This improves the photoelectrochemical performance by facilitating quicker charge transfer between the surfaces of the photoelectrodes. These results are also consistent with photo-catalysis activity.^251^

### 6.8 Measurement of photocurrent

**Fig. 11:**
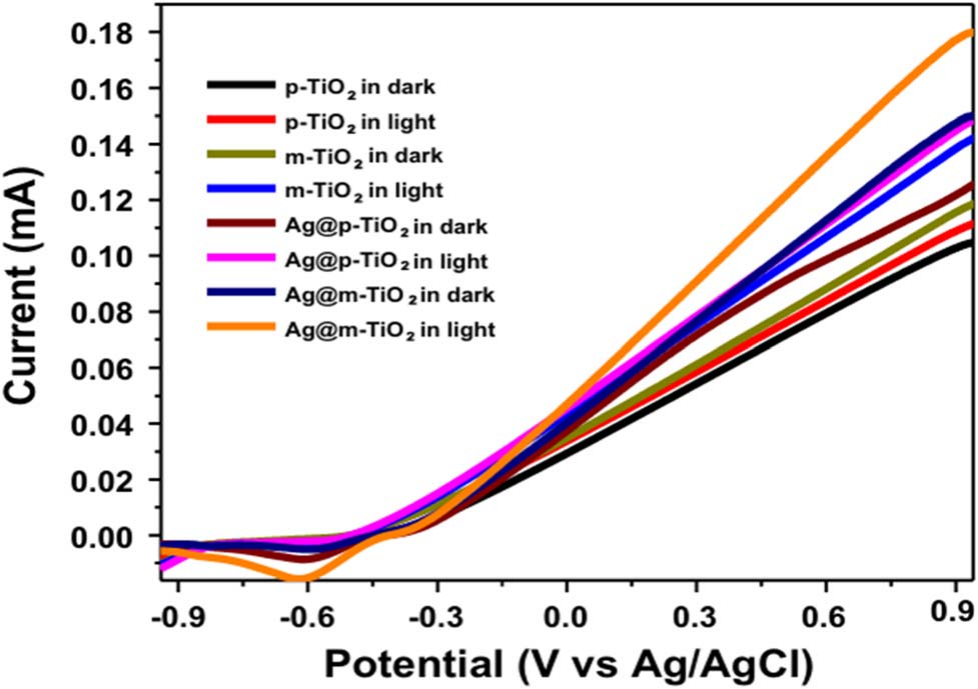
Linear scan voltammograms of the *p*-TiO_2_ and *m*-TiO_2_ NPs as well as Ag@*p*-TiO_2_ and Ag@*m*-TiO_2_ nanocomposites photoelectrodes in the dark and under visible light irradiation.^253^

For the exploration of the synergistic outcomes of the SPR phenomena of SNPs and flaws in *m*-TiO_2_ on the visible light outcome of Ag@*m*-TiO_2_, LSV was implemented for the Ag@*p*-TiO_2_ and Ag@*m*-TiO_2_ nanocomposites under light and in dark along with *p*-TiO_2_*m*-TiO_2_^(107)^ In comparison to the *m*-TiO_2_ NPs, the photoelectrode containing *p*-TiO_2_ NPs showed a reduced photocurrent response; due to wider band gap. Conversely, attaching SNPs to the *p*-TiO_2_ NPs significantly amplified the photocurrent responsiveness. The Ag@*p*-TiO_2_ NPs showed elevated photocurrent than the *p*-TiO_2_ and *m*-TiO_2_ NPs because SNPs exhibit SPR. After the SNPs were anchored at outer area of the *m*-TiO_2_ NPs, the photocurrent reflux significantly enhanced due to the synergistic outcomes of the defects in the NPs and the SPR phenomena of the SNPs. The Ag@*m*-TiO_2_ photocurrent enhancement revealed improvements in both the photo-generated electron–hole pair separation and the photoinduced carrier transport rate. A different theory is that a Schottky junction forms at the metal–metal oxide border, which could help in isolating the holes and photoelectron as well as in increase in the photocurrent.^252^ Observing these results, it was reported that the *m*-TiO_2_ NPs' surface flaws and the SPR phenomena detected on the SNPs work together to enhance their visible light harvesting capabilities (Fig. 11).

## 7. Applications

**Fig. 12:**
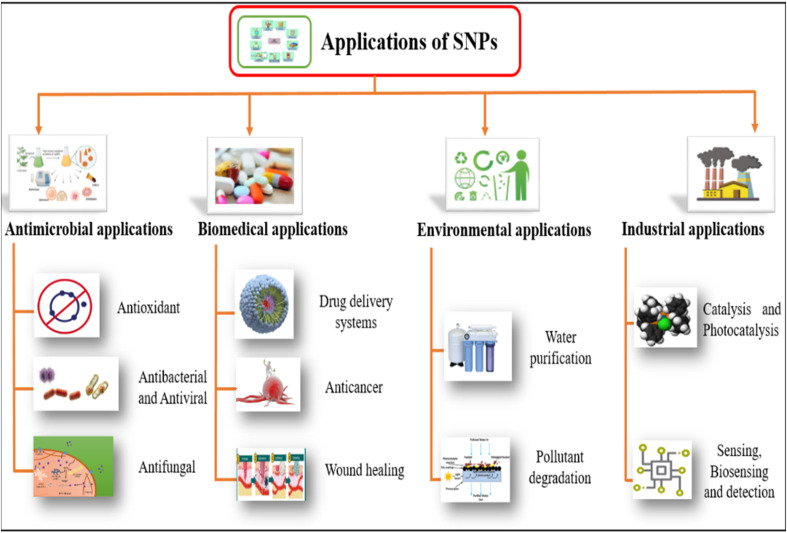
Applications of plants-based SNPs in various sectors.^254^

In recent years, significant progress has been made in the plants-based synthesis of SNPs. Hence being used in tremendous fields including antimicrobial activity, biomedical, environmental and industrial applications. Weather utilized in medicines, cancer treatment, wound healing, drug delivery systems, water purification, pollutant degradation, catalysis or sensing and detection. SNPs have shown strong applications prospects. Applications of SNPs in various sectors have been shown in Fig. 12.

### 7.1 Antimicrobial activity

Silver is a widely known antibacterial ingredient that can effectively combat more than 650 pathogenic organisms, including various types of bacteria (both Gram −ve and Gram +ve), fungi and viruses. SNPs are currently being utilized as a form of metal. Silver has been noted as an agent of healing for numerous ailments in the ancient Indian medical system known as Ayurveda. Starting in 1884, it became widely accepted to apply drops of aq. AgNO_3_ to the eyes of newborns following childbirth in order to stop the spread of *N. gonorrhoea* from affected mothers. Among all the metals with antimicrobial capabilities, silver was discovered to exhibit the most potent antibacterial activity while being the least detrimental to animal cells. Silver gained widespread usage in pharmaceuticals, particularly in the care of injured soldiers during World War I, as a source to inhibit the growth of microorganisms.^255^ The medicinal benefits of silver have been recognized for over two thousand years.^256^ Plant extracts from various sources have been utilized to synthesize SNPs, which were then tested for their antibacterial properties against a range of microorganisms.

#### 7.1.1 Mechanism of action against viruses

**Fig. 13:**
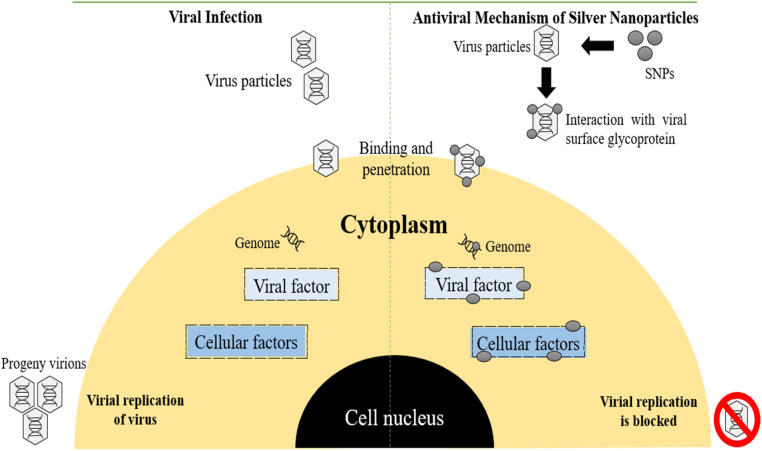
Viral infection and antiviral mechanism of SNPs.^257^

Despite there are some studies on the impact of SNPs on viruses, there is a lack of specific information on the nature of these interactions and mechanism supported universally. However, this may be attributed to the intricate nature of virus structure, which hinders our understanding of the process by which SNPs act on viruses. Salleh *et al.* proposed two mechanisms by which SNPs interact with harmful viruses: (1) SNPs bind to the outer covering of the virus, preventing its binding to cell receptors, and (2) SNPs attach to the RNA or DNA of the virus, inhibiting its reproduction or transmission.^257^Fig. 13 shows the mechanism of action against viruses.

#### 7.1.2 Mechanism of action against bacteria

**Fig. 14:**
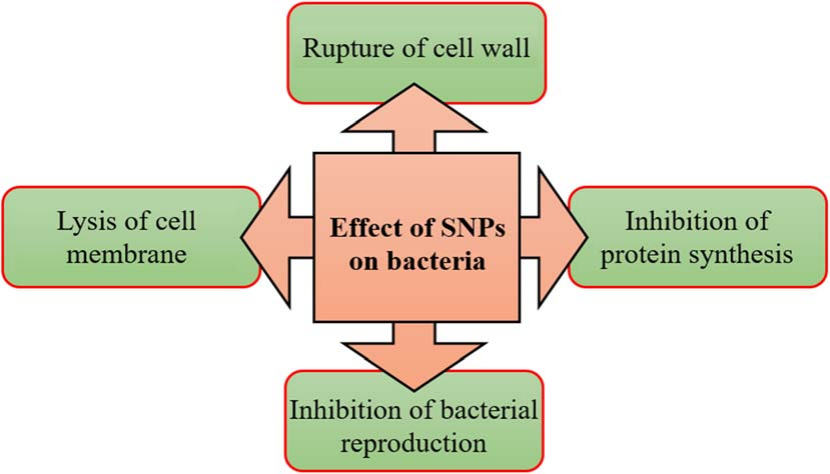
Effect of SNPs on bacteria.^87^

SNPs have a significant function as antibacterial agents. Silver nano formulations have demonstrated a strong capacity to hinder the proliferation of bacteria and other microbes. SNPs effect bacteria in following ways; rupturing of cell wall, lysis of cell membrane, inhibition of protein synthesis, and bacterial reproduction as shown in Fig. 14.^258^

**Fig. 15:**
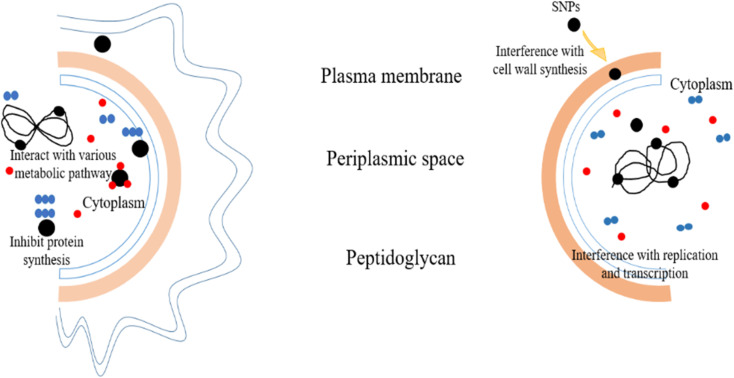
Mechanism of actions (ROS activation) of SNPs against Gram −ve and Gram +ve bacteria.^261^

According to research, SNPs have potent antibacterial properties against both Gram −ve and Gram +ve bacteria. In contrast to Gram +ve bacteria, which possess a thick peptidoglycan layer with a periplasmic membrane, Gram −ve bacteria are characterized by a thin peptidoglycan layer and an extra outer membrane. Research findings indicate that Gram-positive bacteria have a higher degree of resistance to SNPs.^259^ Moreover, literature has indicated that the presence of SNPs has been found to enhance the antibacterial efficacy of certain medicines. Numerous studies have demonstrated the interaction between SNPs and the bacterial membrane, resulting in cell penetration and subsequent disruption of cellular function, generating reactive oxygen species, structural integrity, inhibition of protein synthesis, interaction with various metabolic pathways, interference with replication and transcription and eventual cell death.^260^Fig. 15 shows mechanism of action against bacterial strains.

#### 7.1.3 Mechanism of action against fungi

**Fig. 16:**
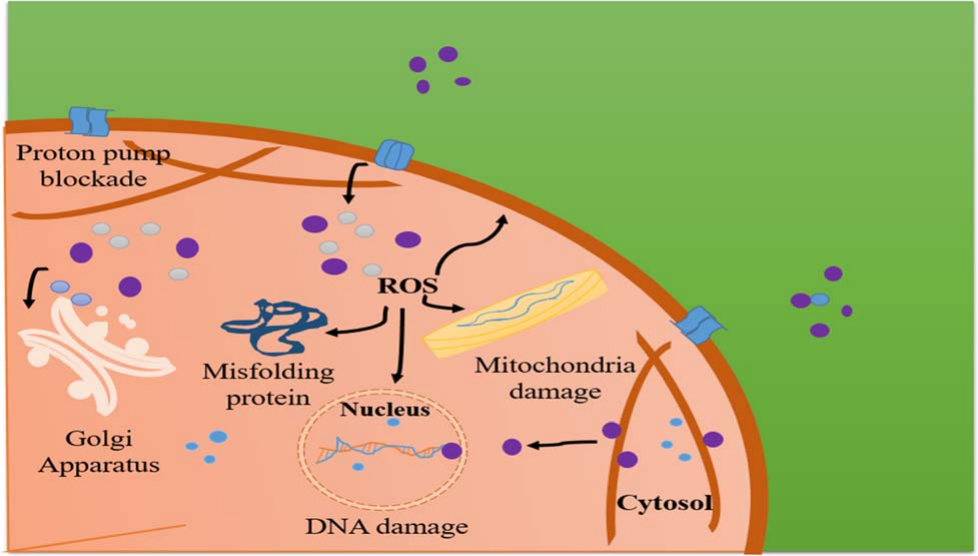
Mechanism of action of SNPs against fungi.^265^

Advancements in recent research pertaining to the composition, framework, and role of fungal cell walls in drug tolerance have facilitated the identification of emerging targets against diseases caused by fungi. Additionally, these advancements have contributed to a deeper comprehension of the mechanisms behind the development of antifungal resistance. SNPs may have a significant influence on the degradation of resistance. These induce surface protein impairment, nucleic acid damage and cellular wall disintegration, through the generation and generation of reactive oxygen species (ROS) and free radicals, as well as the inhibition of proton pumps, interaction with fungal DNA, protein denaturation. One hypothesis is that the presence of SNPs results in the buildup of silver ions, therefore impeding respiration through the outflow of intracellular ions and subsequently causing damage to the electron transport system.^262^ The observed antifungal activity can be related to the smaller size and large surface ratio of NPs. Smaller-sized SNPs have enhanced permeability across cellular membranes. The toxicity of SNPs might be partially ascribed to the generation of ROS, which subsequently induces apoptosis. The hypothesis postulated that the observed toxicity of SNPs *in vitro* can be attributed to either the combined influence of silver ions and SNPs, or their individual effects. Further evaluation is required to elucidate the exact modes of action of SNPs.^263,264^Fig. 16 shows mechanism of action against fungi.

#### 7.1.4 Mechanism of action of antioxidant

**Fig. 17:**
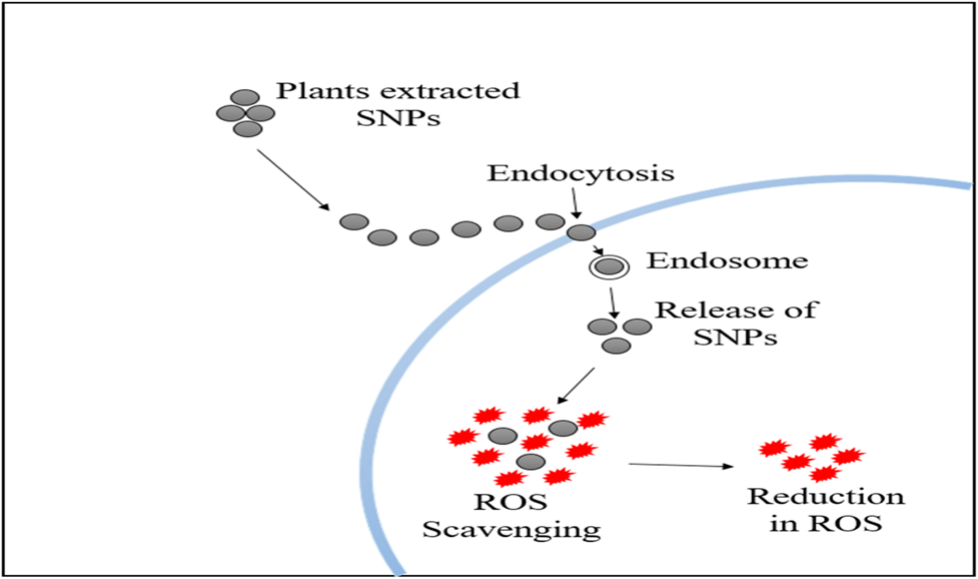
Radical oxygen scavenging mechanism.^266^

Antioxidants obtained from eco-friendly sources have demonstrated extraordinary efficacy in neutralizing free radicals, namely the DPPH radical, also known as DPPH. NPs derived from natural sources have the ability to capture free radicals by several processes, such as chelation of metal ions, enzyme inhibition and direct scavenging of ROS. It is worth mentioning that in certain cases, the natural extract may have a higher antioxidant power than the artificially created NPs, while in other situations, the reverse may be true. The effectiveness of these environment friendly NPs in preventing oxidation depends on the amounts of phenolic compounds and flavonoids found in the extract.^254^ Radical oxygen scavenging mechanism follows endocytosis process for the reduction of ROS. Fig. 17 shows radical oxygen scavenging activity mechanism.

#### 7.1.5 Examples of antibacterial and antifungal studies and results

SNPs have emerged as very promising agents in the ongoing struggle against diseases during the worldwide search for innovative bio medicines. The vast majority of studies supports the notion that SNPs have the power to hinder the growth and trigger the death of harmful bacteria responsible for many human illnesses globally. Currently, plant extracts serve as an invaluable source for the manufacturing of SNPs. The capacity of SNPs to adhere to many biomolecules in microbes enables them to exert a persistent antibacterial influence.^267^Table 5 shows different antifungal and antibacterial activities of SNPs extracted from different plant sources.

**Table 5:** Antifungal and Antibacterial activities of SNPs extracted from different sources

Plant sources	Bacteria	Fungi	Ref.
*Euphorbia hirta*	—	*C. albicans*, *C. kefyr*	268
*Usnea longissima*	*S. aureus*, *S. Pyrogenes*, *S. Viridans*, *C. xerosis*	—	269
*Adathoda vasica*	*V. parahaemolyticus*	—	270
*Svensonia hyderobadensis*	*Proteus mirabilis*	*Fusarium*, *Rhizopus*, *A. flavus*, *A. niger*	271
Green tea	*Klebsiella pneumonia*, *Pseudomonas aeruginosa*	—	272
Green tea	*B. subtilis*, *E. coli*, *S. aures* and *S. pyogenes*	—	273
*Solanum torvum*	*P. aeruginosa*, *S. aureus*	*A. flavus* and *A. niger*	274
*Cucumis sativus* plant extract	*M. tuberculosis*	—	275
*Vigna radiata*	*S. aureus*, *Escherichia coli*	—	276
*Citrus limon*	—	*F. oxysporum*, *A. brassicicola*	277
*Pu-erh* tea leaves	*E. coli*, *K. pneumoniae*, *S. typhimurium*, *S. enteritidis*	—	278
*Boerhavia diffusa*	*A. hydrophila*, *P. fluorescens* and *F. branchiophilum*	—	279
*Argemone mexicana*	*E. coli*; *P. aeruginosa*	*Aspergillus flavus*	280

#### 7.1.6 Examples of antiviral studies and results

The emergence of contagious diseases outbreaks caused by recently identified virulent viruses and those who have developed immunity to existing antiviral medications has driven the search for new antiviral agents.^281^ In the course of the evolution of humanity, viruses have consistently been recognized as extremely fatal human infections. Viruses exhibit pathogenicity by attaching to and invading into the host cell. Avoiding cell infection is best achieved by preventing such penetration and binging.^267^Table 6 shows several plants used to extract SNPs and their antiviral activity.

**Table 6:** Antiviral activities of SNPs extracted from various plants

SNPs extracted from plants sources	Virus	Application	Ref.
*Cinnamomum cassia*	H7N3	Inhibits contaminating the vero cells	282
*Andrographis paniculata*	Chikungunya	Prevents affecting vero cells in a dosage dependent manner	283
*Phyllanthus niruri*	Chikungunya	Prevents affecting vero cells in a dosage dependent manner	283
*Tinospora cordifolia*	Chikungunya	Prevents infecting vero cells in a dosage dependent manner	283
*L. coccineus* hexane	HSV-1, HAV-10, and coxsackie B4	Prevented infection of vero cells	284
*L. coccineus* aqueous SNPs	HSV-1	Prevented infection of vero cells and showed weaker antiviral activity against this virus	284
*M. lutea*	HAV-10 and CoxB4	Prevents infection of vero cells	284

#### 7.1.7 Examples of antioxidant studies and results

Several scholars conducted a study on the antioxidant properties of plant extract-mediated produced SNPs at different times. The antioxidant activity of NPs generated using plant extracts is improved, possibly due to the efficient absorption of antioxidants from the plant extracts onto the surface of the NPs. The disease-fighting abilities of a silver phyto-nanosystem are enhanced by their antioxidant qualities. Therefore, it was shown that silver phyto-NPs obtained from plant extracts possess significant antioxidant activity.^285^ According to Salari *et al.* the SNPs produced using an aqueous extract from the fruit of *Prosopis farcta* have shown exceptional efficacy in removing free radicals.^286^ The same impact was demonstrated in a laboratory setting for a water-based extract of apple,^287^*Indigofera hirsuta*,^288^ and leaf extracts of *Elephantopus scaber*.^289^ Thus, elevated levels of antioxidant phyto-nanoparticle activity may be linked with particular encapsulation of antimicrobial SNPs, particularly for medicinal plants, which contains various bioactive compounds such as polyphenols and flavonoids.^276^

### 7.2 Biomedical applications

#### 7.2.1 Drug delivery system

**Fig. 18:**
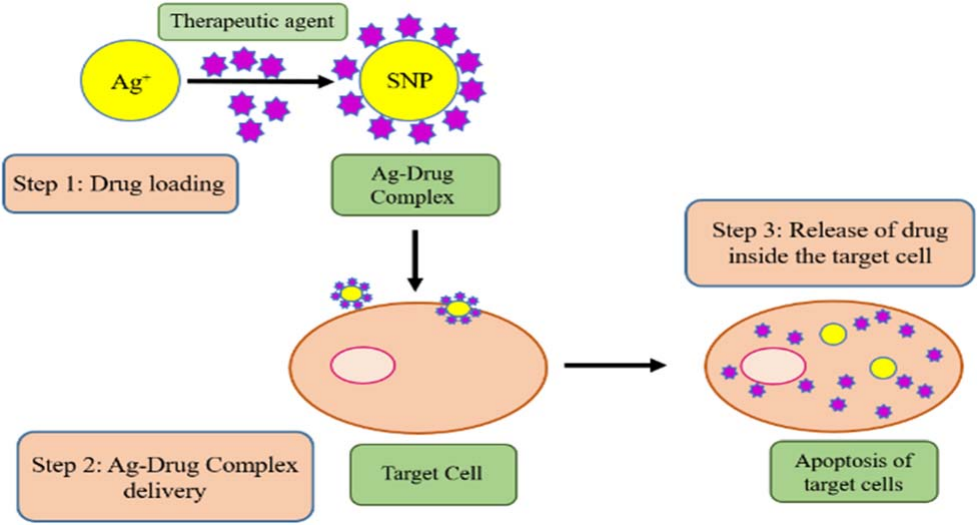
Drug delivery system in the target cell.^292^

Drug delivery by SNPs has shown to be a highly efficacious approach in the management of several medical conditions. The efficacy of drug delivery systems is contingent upon two primary strategies: the gradual and continuous release of drugs, as well as the precise targeting of drug delivery to specified targets. These criteria can be satisfied by employing either active or passive delivery techniques.^290^ SNPs earned significant interest in the realm of designing and advancing innovative drug-delivery systems.^9^ In a more precise manner, the use of green synthesized SNPs has the potential to address the drawbacks commonly connected with current treatments by mitigating their adverse effects and augmenting their effectiveness. The surface properties of SNPs can be modified to improve the targeting capabilities. For instance, positively charged NPs have shown enhanced interaction with negatively charged cell membranes, facilitating targeted drug delivery to specific tissues and cells.^291^Fig. 18 shows the drug delivery system in the target cell.

The integration of green SNPs with anti-cancer medications presents a novel strategy for enhancing disease therapy. By leveraging the SNPs capacity to traverse diverse biological barriers, the direct delivery of pharmaceuticals to tumor tissues may be achieved.^293^ The intercellular drug absorption and distribution are influenced by the size of the NPs by the process of endocytosis. SNPs derived from the extract of *Aerva javanica*, when combined with the anti-cancer medication gefitinib, exhibited greater apoptotic efficacy compared to gefitinib alone when tested on MCF-7 cells.^294^ In addition to its application in cancer, SNPs have been employed in conjunction with anti-seizure medications targeting brain eating amoebae (*Naegleria fowleri*) for the treatment of central nervous system infections. Pharmacological compounds with anti-seizure properties, including diazepam, phenobarbitone, and phenytoin, were incorporated onto the outer surface of SNPs as stabilizing agents. These medications exhibited broad-spectrum anti-amoebic effects against both trophozoite and cyst stages. The conjugation of SNPs with medicines have shown a significant enhancement in fungicidal efficacy against both trophozoite and cyst amoebic phases, in comparison to the individual medications.^295^ Drug delivery involves the transportation of natural or pharmaceutical chemicals to achieve intended therapeutic outcomes. Several preparations utilizing NPs have been documented to have a significant impact on targeting drugs for specific disorders.^87^ Benyettou *et al.* developed a drug-delivery system using SNPs to transport medications like doxorubicin and alendronate directly into cells at the same time. This drug-delivery method has demonstrated the ability to enhance the therapeutic effectiveness of both medications in treating cancer.^296^ A separate study has shown that combining Fe_3_O_4_ and SNPs can serve as effective magnetic hyperthermia mediators with exceptional performance.^297^

#### 7.2.2 Wound healing

**Fig. 19:**
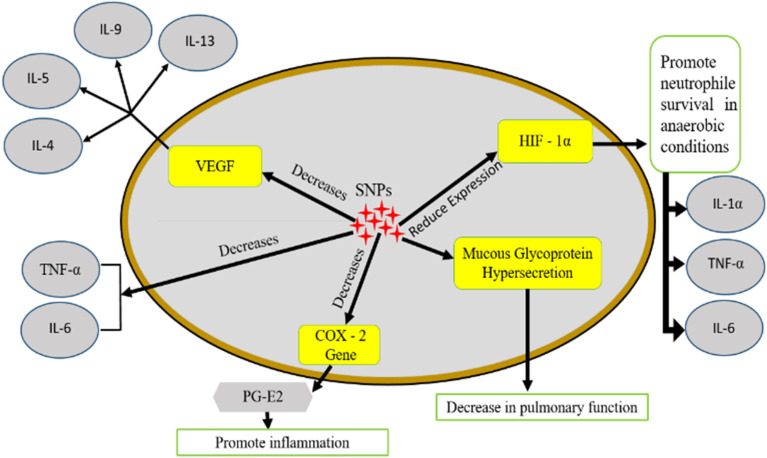
Anti-inflammatory mechanism of plant extracted SNPs for wound healing process.^300^

Wounds arise due to the transection, cutting, tearing, or burning of epidermal tissues in reaction to external stimuli or traumatic events. The classification of wounds encompasses two distinct categories: acute and chronic, delineated by the duration of healing and potential problems. SNPs show considerable anti-inflammatory aspects that can be effective in handling chronic inflammation-related diseases, such as rheumatoid arthritis. Chronic inflammation is often linked to protein denaturation, which NSAIDs aim to inhibit. SNPs have been shown to reduce levels of Vascular Endothelial Growth Factor (VEGF),^298^ a key player in inflammation, by inhibiting the phosphorylation of Src kinase at Y419, thereby decreasing vascular permeability induced by inflammatory mediators like VEGF and IL-1. This action not only reduces mucin hypersecretion but also mitigates the secretion of pro-inflammatory cytokines like TNF-α and IL-12. SNPs suppress the expression of Hypoxia-Inducible Factor (HIF)-1, which regulates genes associated with inflammation and promotes angiogenesis. By inhibiting HIF-1 activity, SNPs prevent the transcription of pro-inflammatory genes and reduce the inflammatory response in tissues. Experimental studies have demonstrated that SNPs can effectively decrease mucin production in lung tissues and alleviate perivascular inflammation in models of allergic responses, highlighting their potential as a safer alternative to traditional anti-inflammatory medications by minimizing side effects while enhancing therapeutic efficacy.^299^Fig. 19 shows anti-inflammatory mechanism for wound healing process.

Scientific evidence has shown that the manufacture of SNP by *Fusarium oxysporum* is precise when conducted *in vivo*. The generated SNPs possess a diameter ranging from 20 to 40 nm. Subsequently, they are combined with Enox for a duration of 28 days in model of *in vivo* burn wound.^301^ In a study conducted by Garg *et al.*, the therapeutic efficacy of biogenic SNPs derived from hydrogel containing *A. nobilis* extract from its root was established. An investigation was conducted to examine the healing efficacy of SNPs with a diameter ranging from 40 to 70 nm and spherical morphology, utilizing an excision wound model. The hydrogel preparation exhibited a substantial enhancement in wound contraction and closure within the initial and subsequent weeks. Over a period of 14 days, the albino rats exhibited a wound healing rate that was 9.34% faster compared to the control group. Conversely, after 21 days, the control group had a wound healing rate that was 1.78% faster than that of the albino group.^302^ SNPs, either alone or in conjunction with anti-bacterial drugs, are frequently employed to facilitate wound healing while preventing infection. In both laboratory settings using fibroblast cell cultures and clinical trials involving patients with partial thickness burns, dressings containing SNPs have been applied. A study has demonstrated that these dressings do not impact the growth of fibroblasts and keratinocytes, which are responsible for the regeneration of healthy skin.^303^

A subsequent investigation conducted a comparison of the effectiveness of two antibacterial substances, specifically nanocrystalline silver and cadexomer iodine. This study conducted a randomized controlled trial on community nursing clients who had leg ulcers that were affected by a high number of germs. Their injuries were cured using either Ag or I dressings. The outcomes substantiated that the utilization of Ag compounds expedited the therapeutic process, resulting in a rapid rate of recovery.^87^ In addition, the combined use of SNPs and antibiotics, namely tetracycline, demonstrates greater efficacy compared to using either SNPs or tetracycline alone in combating bacterial infection. Furthermore, this combined treatment also leads to an increase in wound contraction at a macroscopic level. Moreover, these findings indicate the potential application of a blend of SNPs and anti-bacterial medications in the treatment of infected skin injuries.^304^

#### 7.2.3 Anti-cancer properties

**Fig. 20:**
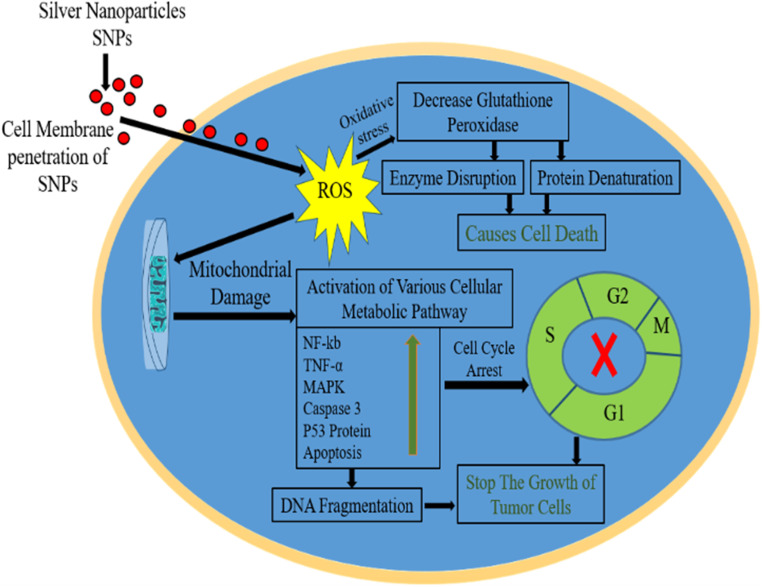
Anticancer mechanism of plant extracted SNPs.^300^

Medicinal plants provide natural products or active substances that have been scientifically demonstrated to play a function in preventing cancer by effectively destroying cancer cells. SNPs have a significant function in inhibiting cancer cells and thus, preventing the formation and progression of the illness. Researchers discovered that SNPs could inhibit the growth of malignant cells by leading to DNA degradation, disruption of mitochondrial membrane potential, ROS generation and oxidation, inhibitory enzymes, controlling signaling pathways, and inhibiting the cell cycle. Furthermore, SNPs can inhibit malignant cells spread by reducing angiogenesis within the lesion or inducing malignant cell apoptosis by deactivating proteins and controlling signaling pathways.^305,306^Fig. 20 shows Anticancer mechanism of plant extracted SNPs.

Research was conducted using lymphoma cell lines to examine the efficacy of SNPs as an antitumor agent in both laboratory settings and living organisms. The work validated the dosage-dependent toxicity of SNPs against lymphoma cells in a controlled laboratory setting, and also suggested their involvement in triggering programmed cell death. Furthermore, there were reports indicating that NPs had a substantial impact on prolonging the survival duration in the mouse model with tumors. Additionally, NPs were shown to play a function in reducing the volume of ascitic fluid in mice that had tumors.^87^

The study investigated the cell damaging and reactive properties of SNPs derived from *P. ginseng* leaves on human cancer cell lines. The results showed that the nanoformulation has antineoplastic efficacy.^307^ Khateef and his colleagues investigated the harmful effects of SNPs at different concentrations. The observation was made that the suppression of cell proliferation was intensified as the quantities of SNPs increased. Furthermore, the rise in the concentration of SNPs resulted in a reduction in cell viability.^308^ In addition, the use of wortmannin, a substance that inhibits autophagy, greatly increased the effectiveness of SNPs in treating melanoma cells.^309^ Numerous investigations have shown the potential of SNPs, including those derived from different plant extracts, to have anti-cancer properties.^310^ Further, studies showed below in Table 7 some other anticancer results of various plants synthesized SNP.

**Table 7:** Anticancer studies of various plants synthesized SNPs

SNPs extracted from plants	Cancer cell line	IC_50_ value (μg mL^−1^)	Ref.
*Curcuma longa* and *Zingiber officinale* mixture	HT-29	150	311
*Solanum trilobatum*	MCF-7	30	312
*Dimocarpus longan*	PC3	10	313
*Punica granatum*	A5449	5	314
*Detarium microcarpum*	HeLa	31.5	315
	PANC-1	84	
*Achillea biebersteinii*	MCF-7	20	316
*Melia dubia*	MCF-7	31.2	317
*Ulva lactuca*	MCF-7	37	318
*Cucumis prophetarum*	HepG-2	94.2	319
*Rosa damascene*	A549	80	320
*Gossypium hirsutum*	A549	40	321
*Syzygium aromaticum*	A549	70	322
*Podophyllum hexandrum*	HeLa	20	323
*Heliotropium indicum*	Siha	20	324
*Gum arabic*	HT-29	1.55	325
	Caco-2	1.26	
*Alternanthera sessilis*	PC-3	6.85	326
*Gracilaria edulis*	PC-3	53.99	327
*Dimocarpus longan*	VCaP	87.33	328

### 7.3 Environmental applications

#### 7.3.1 Water purification

The purification of drinking water is an imperative in contemporary times, given that water derived from various sources has the potential to include pathogenic microbes, heavy metals, and organic compounds at hazardous quantities.^329^ SNPs that possess enhanced stability, cost-effectiveness, and the ability to be controlled in their release rate have demonstrated successful utilization in the elimination of inorganic anions,^330^ heavy metals,^331^ organic pollutants,^332^ and bacteria^333^ from water. These findings indicate a promising outlook for the application of SNPs in the field of water and wastewater treatment. Nevertheless, the direct use of SNPs may result in their aggregation within aqueous environments, so slowly diminishing their effectiveness over an pre-longed duration.^334^ In the present context, the use of SNPs affixed to filter materials presents a potentially superior option for mitigating the issue of aggregation, while also offering cost-effectiveness and demonstrating good antibacterial properties, as evidenced by many research.^335^ The deposition of SNPs on cellulose fibres has demonstrated notable antibacterial efficacy against *Escherichia coli* infections. Furthermore, the loss of Ag^+^ from these sheets does not surpass the established target level of 0.1 ppm for drinking water, as determined by the environmental protection agency and the World Health Organization.^336^ Furthermore, there has been a notable rise in the use of SNPs that are integrated into ceramic materials and membranes for the purpose of disinfecting and treating water intended for domestic consumption at the point of consumption over the past twenty years.^337^ An additional use of SNPs in the field of water treatment is the prevention of fouling in membrane filters employed inside water treatment systems.^338^ The SNPs may be readily detached from the beads and effectively suppress the proliferation of microorganisms in an actual water sample. Utilizing paper impregnated with SNPs to facilitate the passage of bacterially tainted water might serve as a very efficient method for emergency water treatment.^339^ The polyacrylonitrile (PAN) sorbent, employed in the process of water treatment, effectively retains harmful bacteria on its surface. Nevertheless, the application of SNPs prevented the occurrence of biofilm growth on the surface.^340^ The water purification membrane, which was created by incorporating 1 mg L^−1^ of biosynthesized SNPs onto nitrocellulose membrane filters, effectively eliminated the microbial population of *E. coli*, *E. faecalis*, *P. aeruginosa*, and *S. aureus* suspensions. Furthermore, it achieved a significant reduction in the presence of *E. coli* and *S. aureus*, with reductions of up to 6 and 5.2 orders of magnitude, respectively.^341^

#### 7.3.2 Pollutant degradation

The catalytic activity of SNPs is triggered by the transfer of electrons from silver ions to a reducing substance (e^−^ donor) and a dye (e^−^ acceptor).^342^ That depend on its dimensions, form, and surface characteristics, as well on their overall and surface composition. Catalysts composed of tubular nanocomposites with tiny SNPs exhibited superior catalytic performance compared to those with larger NPs.^343^ The SNPs produced by biosynthesis showed a potent chemo catalytic effect, leading to the complete breakdown of 4-nitrophenol into 4-aminophenol,^344^ methyl orange, and methylene blue using sodium borohydride.^345^ The rate constantly exhibited a positive correlation with the quantity of SNPs employed as a catalyst. In addition, SNPs have been used as nano catalysts to break down dyes in wastewater and effluents. They also possess distinctive features that are essential for the process of carbon dioxide electrolysis, which plays a major role in turning CO_2_ into CO.^346^ SNPs function as a catalyst with different properties in the process of eliminating halogenated organic contaminants using BH_4_.^347^ The involvement of SNPs in the process of photocatalytic degradation of pollutants such as crystal violet, methylene blue, and malachite green has been investigated.^348^ SNPs enhance the photocatalytic activity of metal oxide by altering the absorption of the visible region.^349^

### 7.4 Industrial applications

#### 7.4.1 Catalysis

The use of NPs for water filtration may be categorized into three main kinds of contaminants: halogenated organics (such as pesticides), heavy metals, and micro-organisms.^338^ The characteristic catalytic degradation mechanism often entails the adsorption of pollutant molecules onto the surface of the nanoparticle, which is then followed by electron transfer activities. SNPs possess notable characteristics such as a substantial surface area, reactivity, and tunable features, rendering them very efficient catalysts for the degradation of dyes.^350^ Various parameters can exert an impact on the catalytic activity, including the size and shape of SNPs, the presence of capping agents, the concentration of SNPs, as well as the pH and temperature conditions. Particles of smaller size that possess a greater surface area have enhanced catalytic activity.^351^

In the presence of sodium borohydride, SNPs derived from extracts of *Hyptis capitata* leaves, fruits, and stems exhibited catalytic reduction of methyl orange colour. The reaction reached completion within a time frame of 3 hours, suggesting that the SNPs effectively facilitated the reduction of the azo-dye.^350^ The synthesis of SNPs utilizing an extract derived from the *Sphagneticola procumbens* plant shown the ability to photocatalytically degrade the harmful Orange G and Direct Blue-15 azo dyes when exposed to UV radiation.^351^ Printing and dye wastewater provide a significant challenge due to their intricate composition among the several forms of wastewater.^352^ The substantial quantities of non-biodegradable oil and hazardous 4-nitrophenol that are dissolved in wastewater present an immediate and pressing concern.^353^ SNPs play a crucial role in the catalytic degradation of 4-nitrophenol^354^ because of their large specific surface area, numerous showed low-coordination sites, and affordable price. Nevertheless, as a result of the surface's elevated energy, there is a propensity for diminished catalytic activity over time. Furthermore, the retrieval of the granules from the water is exceedingly challenging, hence resulting in potential secondary pollution. Consequently, the act of immobilizing SNPs within porous oil absorbent materials has been found as a highly successful method for enhancing the capacity to reuse SNPs.^355^ Melinte *et al.* synthesized several photo catalysts using silver, gold, or silver-gold NPs supported on photo cross-linked natural materials. These hybrid structures facilitated the photo-catalytic breakdown of polymers, namely 4-nitroaniline.^356^ Roy *et al.* investigated the process of breaking down methylene blue dye using biogenic SNPs that were created using (yeast) *S. cerevisiae* extract. In conclusion, various well-documented publications have demonstrated the catalytic degrading effectiveness of SNPs, indicating their potential utility in water treatment systems. When constructing a water treatment system using SNPs, it is important to take into account the potential discharge of these NPs and the possibility for secondary water pollution.^357^ A separate investigation resulted in the effective production of multifunctional 3D filter cotton by immobilizing PDA and SNP on the surface. The treated 3D filter cotton has exceptional catalytic degradation capabilities, making it suitable for the decomposition of water-soluble 4-nitrophenol. Additionally, it demonstrates remarkable effectiveness in separating oil and water, as well as the capacity to be reused.^358^ A research has described the use of green synthesis to produce very stable SNPs, which have demonstrated effectiveness as catalysts, photocatalysts, and antibacterial agents in treating wastewater.^359^

#### 7.4.2 Photocatalytic activity

Photocatalytic activity is the speedup of a photoreaction with the help of a catalyst, which in this case is often SNPs. These NPs have received a lot of interest because of their unusual features and ability to degrade organic contaminants, notably synthetic colors, when exposed to light. The use of plant extracts to synthesize SNPs not only provides an environmentally preferable alternative, but it also improves their photocatalytic capabilities. SNPs have been deeply studied for their capacity to breakdown dyes that are frequently found in industrial effluent. The photocatalytic activity of these NPs is increased by their large surface area and the formation of ROS when exposed to light. Photocatalytic activity by using various plant sources can be used to degrade dyes, organic pollutants and waste water treatment.^181^ Photocatalytic activity of SNPs extracted using several plants sources are given in the following Table 8.

**Table 8:** Photocatalytic applications of SNPs extracted from different sources

SNPs extracted from plants	Photocatalytic application	Ref.
*Zingiber officinale* (ginger)	Degradation of methylene blue and methyl orange	360
*Brassica oleracea* (cauliflower)	Removal of dyes or organic pollutants	361
*Coriandrum sativum* (parsley)	Effective degradation of industrial dyes under UV light	362
*Camellia sinensis* (green tea)	Photocatalytic reduction of organic pollutants and dyes	363
*Ananas comosus* (pineapple)	Efficient removal of organic dyes in wastewater treatment	364
*Capsicum annuum* (red pepper)	Photocatalytic treatment of wastewater pollutants	365
*Citrus sinensis*	Removal of textile dye pollutants in water	366
*Pandanus atrocarpus*	Degradation of environmental organic pollutants	367
*Eucalyptus globulus*	Effective degradation of methylene blue dye	368
*Piper chaba*	Effective photocatalytic degradation of organic dyes	63
*Azadirachta indica* (neem)	Photocatalytic removal of environmental contaminants and heavy metals	369

#### 7.4.3 Sensing and detection

Mobile and wearable medical gadgets might be an excellent option for observing patients. SNPs, because of their highly conductive nature, find uses in stretchy sensors.^370^ Notably, colorimetric, optical, and electrochemical sensors have demonstrated their efficacy in the analysis of environmental materials.^371,372^ An essential use of nanotechnology in the field of electro analysis is the modification of sensors using NPs to achieve cost-effective, highly sensitive, and selectively responsive devices.^373^ These sensors are created by distributing or covering SNPs on the surface of carbon-based nanomaterials' surface such fragmented graphene sponges, multiwalled carbon nanotubes onto polydimethylsiloxane, reduced graphene oxide, and carbon black.^374^ The prepared sensors have been used for producing electrocardiograms.^375^ for showing exceptional temperature-sensing aspects, *etc.*^370^ SNPs may be easily synthesized, and several batches consistently provide SNPs with uniform size, shape and dispersion. Therefore, they are appropriate for SERS sensors. It has been discovered that the intensity of SERS diminishes as the distances between particles rise.^376^ SNPs are also employed as a substrate in SERS to detect sialic acid linked with breast cancer, prostate-specific antigen (a biomarker for prostate cancer), and infertility.^377^ Colorimetric sensors are technological instruments that provide the real-time detection of an analytes in a given sample by providing visual information in the form of colour.^378^ Karimi and Samimi employed a colorimetric sensor and SNPs derived from the algae *Chaetomorpha* spiral to quantify the presence of mercury ions (Hg^2+^) in both mineral and tap water across a broad concentration spectrum ranging from 0.01 to 200 mmol L^−1^.^379^ SNPs are utilized as colorimetric sensors for the detection of carbohydrate antigen 125 (CA125), that is present on numerous ovarian cancer cells.^380^

#### 7.4.4 Biosensing

Plant-extracted SNPs are gaining significant popularity for biosensing applications due to their unique features, notably in the detection of environmental contaminants and biomolecules. SNPs have unique qualities, such as strong electrical conductivity and large surface area, making them ideal for developing advanced biosensors. They can function as electron transfer enhancers between biomolecules and electrodes, increasing the sensitivity and selectivity of sensors.^381^ Different plant sources extracted SNPs used in biosensing applications are given below in Table 9.

**Table 9:** Biosensing applications of SNPs extracted from various plant sources

SNPs extracted from plant sources	Applications	Ref.
*Citrus sinensis*	Detection of heavy metals and glucose	382
*Piper chaba*	Detection of methylene blue dye	63
*Psidium guajava*	Detection of glucose	305
*Piper longum*	Detection of bacterial infections	231
*Curcuma longa* (turmeric)	Biomarker detection	305
*Azadirachta indica*	Detection of pathogens	383

## 8. Advantages and challenges of using plants-based synthesis methods

The advantages and challenges of plant-based synthesis of SNPs highlight both the potential and the limitations faced in the innovative field.

### 8.1 Benefits of using plants-based synthesis of SNPs methods

The benefits of using plants-based synthesis methods are below.

#### 8.1.1 Environmental friendliness

The plant-based production of SNPs follows the principles of green chemistry, which prioritize the use of non-toxic substances and methods that reduce harm to the environment. This approach employs plant extracts as stabilizing and reducing agents, therefore substantially decreasing the requirement for toxic chemicals commonly employed in traditional synthesis procedures. This method not only reduces pollution but also encourages the sustainable utilization of natural resources.^384^ SNPs synthesized using green methods can be utilized for environmental remediation purposes, including water treatment and soil cleansing. Their capacity to engage with contaminants and diseases renders them highly helpful in tackling environmental concerns.^385^

#### 8.1.2 Biocompatibility

SNPs developed from plant extracts are often more biocompatible and less poisonous than those synthesized using chemical procedures. The inherent chemical constituents found in plant extracts enhance the safety and durability of the NPs, rendering them appropriate for many biological uses without causing substantial hazards to human well-being or the ecosystem.^254^

#### 8.1.3 Sustainability

Utilizing sustainable plant resources for the production of NPs contributes to the advancement of sustainability. Plants include a wide range of bioactive chemicals that help to decrease silver ions, enabling effective production under gentle settings. In contrast to traditional procedures, which can need significant energy inputs and produce hazardous by-products.^386^

#### 8.1.4 Cost-effectiveness

The utilization of plant-based extraction method for the production of SNPs is frequently more economical compared to conventional approaches. The use of easily accessible natural substances reduces the necessity for costly chemical agents and intricate apparatus, resulting in substantial reductions in manufacturing expenditures. Research suggests that the process of green synthesis can result in cost reductions of up to 40% when compared to traditional approaches.^254^

#### 8.1.5 Availability of plant-based synthesis methods

The accessibility of the plant-based synthesis method enables convenient scalability, rendering it viable for large-scale manufacturing. Scalability is crucial for satisfying industrial requirements without significant rises in expenses or intricacy.^386^

#### 8.1.6 Energy storage and economic viability

The use of agro-industrial waste for synthesis promotes economic viability by transforming waste into profitable goods.^254^ Green synthesis processes often need fewer energy inputs, hence leading to total cost reductions. Studies have demonstrated that employing these approaches can result in a 30% decrease in energy usage when compared to conventional synthesis methods, rendering them more environment friendly and financially feasible.^254,385^ Due to the growing demand for environment friendly products, SNPs produced from plants are expected to gain more market recognition. Their distinct characteristics and reduced ecological footprint provide them a favorable position in competitive marketplaces, hence improving their economic feasibility.^386^

### 8.2 Challenges and limitations

The challenges of plants-based synthesis of SNPs primarily revolve around variability in synthesis methods, scalability and reproducibility of results.

#### 8.2.1 Variability

A significant challenge is the lack of consistency in the chemical composition of plant extracts, which can result in differences in size, shape, and stability of the synthesized SNPs. Plants have various concentrations of phytochemicals, including flavonoids and phenolic acids, which function as reducing and stabilizing agents. The diversity in these compounds plays a crucial role in stabilization and reduction of silver ions in SNPs. For example, secondary metabolite like flavonoids, terpenoids, and phenolic compounds plays crucial role in nanoparticle formulation. The variability can impact the efficacy of silver ion reduction and the ultimate characteristics of the NPs. Different environmental conditions such as soil type, climate, and cultivation methods can influence the phytochemicals concentration. This variability could result in batch-to-batch inconsistencies during SNPs synthesis. Batch to batch variability in composition is a challenge that needs to be minimized. For instance, plants grown in nutrient rich soils might produce higher concentrations of polyphenols, which could result in better reduction of silver ions and higher nanoparticle yield. Higher concentrations of silver ions can lead to agglomeration while lower concentration may lead to fewer nanoparticles. A delicate balance must be maintained to achieve an optimal yield of well dispersed SNPs. Due to inconsistency nanoparticles characteristics such as size, shape, and stability can vary. Moreover, composition of plant extracts can vary based on several factors like plant species, growth conditions, seasonality, and geographical location.^387^

#### 8.2.2 Understanding the mechanism and standardization issues

The exact biochemical pathways and mechanisms by which plant extracts reduce silver ions to SNPs are not fully understood. This makes the work harder to predict and control the synthesis.^216^ There is no universal protocol for plants-based synthesis of SNPs making it difficult to replicate results across different studies. Different plants are used due to which size shape, stability and yield differs and limits the research.^94^

#### 8.2.3 Scalability

The content of plant extracts can exhibit substantial variation depending on factors such as geographical location, seasonal fluctuations, and extraction techniques. The unpredictability maintaining consistent reaction conditions in SNPs synthesis might hinder the capacity to scale up production while ensuring consistent nanoparticle size and characteristics.^387^ Expanding the synthesis process focuses meticulous optimization of several factors, such as pH, temperature and the concentration of plant extracts and silver ions. The process of optimization can be intricate and time-consuming, as the optimal conditions for small-scale synthesis may not simply correspond to higher sizes.^385^ The duration of the synthesis process can be prolonged as the scale increases. Increased quantities might result in extended response durations, thereby impacting the effectiveness and financial feasibility of the procedure. Raising output may need the inclusion of more infrastructure, such as specific reactors and purification systems, which might raise the initial investment expenditures and operating complexities. Different extraction techniques can yield different concentrations and quality of active phytochemicals. Variations in extraction conditions like temperature, time and solvent type can lead to differing yields. Inefficient extraction led to insufficient reducing agents for effective nanoparticle synthesis. Plants based synthesis is more economical than traditional methods, but in large scale operations may incur higher costs due to the need for larger quantities of raw materials, extraction and processing equipment. The availability of specific plant materials can be seasonal or limited by geographical factors. This variability can affect the scalability of SNP production since consistent access to high quality raw materials is crucial for large scale production.^384^

#### 8.2.4 Seasonal and geographical factors

Different plants or even different parts of the same plants contain various concentrations of phytochemicals, which leads to different size, shape and stability of synthesized SNPs.^328^ The concentrations of active compounds may vary depending upon the seasonal and geographical locations where plants are grown affecting the synthesis of process.^388^

#### 8.2.5 Reproducibility

Obtaining consistent results in terms of nanoparticle size, shape, and stability can be challenging due to the natural diversity in plant extracts. This lack of consistency can hinder the dependability of the synthesized SNPs for usage in manufacturing. Currently, there is no accepted worldwide procedure for creating SNPs utilizing plant extracts. The absence of uniformity in procedures can result in variations in outcomes among various laboratories and research, making it difficult to compare data and identify optimal methods.^387^ Characterizing SNPs is essential for interpreting their characteristics and possible uses. Nevertheless, the characterization techniques employed might produce varying outcomes based on the synthesis conditions and the particular plant extracts utilized. This heterogeneity might also hinder the capacity to reproduce results.^385^ Our current knowledge of the methodologies involved in the biosynthesis of SNPs utilizing plant extracts is very restricted. The lack of understanding in this area might result in unexpected impact and challenges while producing effective synthesis techniques. Variability in plant composition such as species differences, environmental factors, extraction method, plant material availability and process controlling conditions highly affects reproducibility of SNPs.^389^

#### 8.2.6 Potential toxicity

SNPs have wide range of applications in various fields but on other hand it poses certain risks.^390^ These nanoparticles may potentially be toxic to aquatic organism and their wide spread use as antimicrobial agents and disinfectants could contribute to the development of bacterial resistance.^391^ As the release of nano silver from SNPs may cause level to increase toxic threshold. Studies aimed at determining the toxicity limit of SNPs are insufficient as the toxicity depends on various factors, such as the concentration, size, shape, and surface area of the nanoparticles. Moreover, factors like the source of SNPs, the route of entry into the body, the methods of toxicological assessment and the units of measurement can significantly influence the results, making it challenging to establish a precise toxicity range for SNPs.^339^

### 8.3 Environmental and economic aspects

The conventional techniques employed for manufacturing NPs are expensive, toxic, and environmentally unfriendly. In order to cover these problems, scientists have identified the precise green routes, which are naturally existing origins and their constituents that may be utilized to produce NPs through synthesis. The origin of sustainable synthesis may be classified into 3 groups: (a) employing microorganisms such as fungus, yeasts, bacteria, and actinomycetes, (b) utilizing plants and its extracts, and (c) employing membranes, viral DNA, and diatoms. This paper primarily examines the process of synthesizing SNPs utilizing plant extract using a method known as green synthesis.^386^ The use of plants and its extracts in green synthesis has become more popular because of its rapid advancement, simplified procedure, cost-efficient approach, lack of pathogenicity, and environmentally sustainable nature.^216^

The utilization of plants extracts in the green synthesis of SNPs is in accordance with the ideals of sustainable development. This approach decreases the dependence on harmful substances and reduces environmental contamination linked to conventional chemical synthesis, which frequently contains dangerous ingredients. Conventional synthesis processes frequently produce hazardous waste, but plant-based synthesis substantially decreases trash generation. The use of water or ethanol as solvents also enhances the eco-friendliness of this method.^384,386^ Plant-based approaches often provide SNPs that exhibit higher biocompatibility in comparison to chemically synthesized ones. The presence of phytochemicals in plant extracts serves as both stabilizing and reducing agents, hence improving the safety profile of NPs used in biomedical applications.^385,386^ Utilizing renewable plant resources for nanoparticle synthesis encourages the adoption of environmentally sustainable techniques. Plants include several active chemicals that help in reduction of silver ions, enabling the synthesis process to be more efficient with less energy and milder conditions.^392^

The eco-friendly production of SNPs is more cost-effective compared to traditional approaches. Plant extracts are easily accessible and affordable, obviating the necessity for expensive chemical reagents and intricate equipment. This facilitates the procedure for large-scale manufacture. The simple nature of the plant-based synthesis method enables convenient expansion to suit industrial requirements without substantial cost escalation. The capacity to scale is essential for commercial applications in many domains such as health, agriculture, and environmental remediation.^384^ The economic feasibility is further supported by the wide-ranging uses of plant-derived SNPs, which encompass several sectors including agriculture (for pest management), medicine (as antibacterial agents), and environmental remediation (for water purification). Their market worth is enhanced by their wide-ranging application.^385^

## 9. Future perspectives

The current progress in the green synthesis of SNPs highlights an important shift towards sustainable and eco-friendly manufacturing methods. Using biological resources, including plant extracts and agricultural waste, offers a new method that reduces adverse environmental effects while increasing the value of byproducts from the food industry. Plant extracts, including those from *Citrus sinensis* and pomegranate,^393,394^ have demonstrated effectiveness in transforming silver ions into NPs that possess significant antibacterial characteristics. In addition, the utilization of agricultural waste such as banana,^394^ potato^395^ and onion peels^396^ not only decreases waste but also acts as a cost-efficient source for the synthesis of SNPs, exhibiting antibacterial and antioxidant properties.^390^ Generally smaller sized NPs have enhanced antibacterial activity as a result of increased surface area that comes into proximity with the microbial cell. Within the same size range, the antibacterial activity of SNPs follow this order: triangular > pentagonal, hexagonal, cubic, nano-rod > spherical. The triangular shape exhibited the maximum level of activity mostly because of its superior edge fitting, which is attributed to its sharp edges and the dominating stable (1 1 1) facet.^391,397^ Hexagonal, cubic, and nano-rod NPs possess curved edges, which may potentially decrease their effectiveness against bacteria in comparison to triangular-shaped NPs. On the other hand, NPs with a spherical form that have no sharp edges and are mostly composed of (1 0 0) face exhibited the lowest antibacterial effects.^398^ Discovery and utilization of a broader range of plants could pave way for further emerging trends in plants-based synthesis of SNPs. The enhanced biocompatibility and decreased toxicity of SNPs produced using environment friendly techniques are gaining more recognition, particularly in the field of medical applications such as cancer therapy. These NPs demonstrate increased cytotoxicity against cancer cells while protecting the structural integrity of normal cells.^399^ The several applications of these NPs encompass antibacterial and antifungal purposes, drug delivery systems, environmental remediation, and catalysis in chemical reactions.^400^

Some other key future directions which should be focused are to establish a standardized protocol that can widely be adopted to minimize the variability in the synthesis of NPs. This may include innovative methods like automated systems for high-throughput screening of plant extracts, enabling the rapid identification of most effective plant species and conditions for SNPs synthesis.^328^ In depth mechanistic studies can be done by using advanced techniques like genomics, proteomics, and metabolomics can be utilized to understand the molecular interactions between plant phytochemicals and silver ions, leading to more precise and predictable synthesis processes.^216,388^ The integration of plant-based synthesis of SNPs with other biotechnologies, such as biosensors, biocatalysts or bioimaging could lead to innovative applications in diagnostics, environmental monitoring and industrial processes.^94^ Characterization methods for SNPs have made great progress, offering valuable information about their dimensions,^395^ morphology, and surface characteristics,^401,402^ which are crucial for their performance. UV-vis spectroscopy,^401,403^ XRD,^403^ and high-resolution transmission electron microscopy^402^ have been used to verify the production and examine the structural properties of SNPs. By employing FT-IR analysis,^404^ we can determine the specific functional groups that are responsible for both stabilizing and reducing SNPs. This study contributes to a deeper comprehension of the synthesis process.^399^

In the future, SNPs made from steamed plant extracts show great potential for being used in several industries. These NPs have the potential to enhance wound healing, facilitate drug delivery, and optimize cancer therapy in the field of biomedicine.^384,405^ Their capacity as natural preservatives in the food industry has the potential to transform food safety by prolonging shelf life without the use of synthetic chemicals.^406^ Moreover, their efficacy in environmental remediation, including tasks like water purification and soil restoration, establishes them as useful agents in tackling pollution. SNPs may be utilized in cosmetics manufacturing to enhance items due to their antioxidant and antibacterial capabilities. Similarly, in the textile industry, SNPs provide antibacterial effects and contribute to self-cleaning features. Their optical characteristics also render them appropriate for the creation of sensors and their utilization in the fields of electronics and optoelectronics. The collaboration between researchers in materials science, chemistry, biology, medicine, and engineering is essential for fully realizing the capabilities of SNPs and promoting innovation in various fields. Future research will delve deeper into the mechanisms by which plant extracts reduce and stabilize SNPs. Understanding these processes at a molecular level will help in optimizing synthesis protocols and improving the reproducibility and scalability of nanoparticle production.^384^

## 10. Conclusion

The green synthesis of SNPs using plant extracts has been a sustainable, economically effective, and eco-friendly substitute to traditional chemical methods. The characterization of these SNPs using techniques such as FTIR, XRD, SEM, TEM, SAED, EIS, and LSV-UV discovered their high-quality, crystalline morphology and appropriate physical and chemical characteristics. The synthesized SNPs exhibited remarkable performance across a range of efforts, including photocatalysis for environmental cleanup, antimicrobial activity, and boosted resources in biomedical and biosensing technologies. This review emphasizes the potential of green-synthesized SNPs in focusing global confronts related to environmental pollution, healthcare, and industrial processes. The biocompatibility and functional adaptability of these SNPs opens new routes for sustainable nanotechnology, with wide-accomplished implications for future scientific advancements and practical applications. Further research of plant-based materials and accessible production techniques will increase the pertinency of SNPs in real-world scenarios. By addressing unresolved issues, green synthesis can develop into a more robust and scalable knowledge, paving the way for groundbreaking solutions to global trials in environmental sustainability, healthcare, and resources. This expedition towards harnessing the full potential of green synthesised SNPs underscores the importance of interdisciplinary research in shaping a sustainable opportunity.

## Data availability

No primary research results, software or code have been included and no new data were generated or analysed as part of this review.

## Conflicts of interest

There are no conflicts to declare.

## References

[cit1] Yakut Ş. M. , Karataş M., Green Engineering in Nanosynthesis and its Place in Environmental Engineering, 2021

[cit2] Rafique M., Sadaf I., Rafique M. S., Tahir M. B. (2017). A review on green synthesis of silver nanoparticles and their applications. Artif. Cells, Nanomed., Biotechnol..

[cit3] Ponsanti K., Tangnorawich B., Ngernyuang N., Pechyen C. (2022). Synthesis of mesoporous silica nanoparticles (MSNs)/silver nanoparticles (AgNPs): promising hybrid materials for detection of breast cancer cells. J. Mater. Sci.: Mater. Electron..

[cit4] Jayeoye T. J., Eze F. N., Olatunde O. O., Singh S., Zuo J., Olatunji O. J. (2021). Multifarious biological applications and toxic Hg2+ sensing potentiality of biogenic silver nanoparticles based on securidaca inappendiculata hassk stem extract. Int. J. Nanomed..

[cit5] Ontong J. C., Singh S., Nwabor O. F., Chusri S., Voravuthikunchai S. P. (2020). Potential of antimicrobial topical gel with synthesized biogenic silver nanoparticle using Rhodomyrtus tomentosa leaf extract and silk sericin. Biotechnol. Lett..

[cit6] Priyadarshini J. F., Sivakumari K., Selvaraj R., Ashok K., Jayaprakash P., Rajesh S. (2018). Green synthesis of silver nanoparticles from propolis. Res. J. Life Sci., Bioinf., Pharm. Chem. Sci..

[cit7] Mohammadi F., Yousefi M., Ghahremanzadeh R. (2019). Green synthesis, characterization and antimicrobial activity of silver nanoparticles (AgNPs) using leaves and stems extract of some plants. Adv. J. Chem., Sect. A.

[cit8] Syafiuddin A., Salmiati S. M. R., Beng Hong Kueh A., Hadibarata T., Nur H. (2017). A review of silver nanoparticles: research trends, global consumption, synthesis, properties, and future challenges. J. Chin. Chem. Soc..

[cit9] Burduşel A.-C., Gherasim O., Grumezescu A. M., Mogoantă L., Ficai A., Andronescu E. (2018). Biomedical applications of silver nanoparticles: an up-to-date overview. Nanomaterials.

[cit10] Tavaf Z., Tabatabaei M., Khalafi-Nezhad A., Panahi F. (2017). Evaluation of antibacterial, antibofilm and antioxidant activities of synthesized silver nanoparticles (AgNPs) and casein peptide fragments against Streptococcus mutans. Eur. J. Integr. Med..

[cit11] Domeradzka-Gajda K., Nocuń M., Roszak J., Janasik B., Quarles Jr C. D., Wąsowicz W. (2017). *et al.*, A study on the *in vitro* percutaneous absorption of silver nanoparticles in combination with aluminum chloride, methyl paraben or di-n-butyl phthalate. Toxicol. Lett..

[cit12] Kim W., Kim W. K., Lee K., Son M. J., Kwak M., Chang W. S. (2019). *et al.*, A reliable approach for assessing size-dependent effects of silica nanoparticles on cellular internalization behavior and cytotoxic mechanisms. Int. J. Nanomed..

[cit13] Adur A. J., Nandini N., Mayachar K. S., Ramya R., Srinatha N. (2018). Bio-synthesis and antimicrobial activity of silver nanoparticles using anaerobically digested parthenium slurry. J. Photochem. Photobiol., B.

[cit14] Shayo G. M., Elimbinzi E., Shao G. N. (2024). Preparation methods, applications, toxicity and mechanisms of silver nanoparticles as bactericidal agent and superiority of green synthesis method. Heliyon.

[cit15] Singh S., Chunglok W., Nwabor O. F., Ushir Y. V., Singh S., Panpipat W. (2022). Hydrophilic biopolymer matrix antibacterial peel-off facial mask functionalized with biogenic nanostructured material for cosmeceutical applications. J. Polym. Environ..

[cit16] Alexandre N., Ribeiro J., Gaertner A., Pereira T., Amorim I., Fragoso J. (2014). *et al.*, Biocompatibility and hemocompatibility of polyvinyl alcohol hydrogel used for vascular grafting—*In vitro* and
in vivo studies. J. Biomed. Mater. Res., Part A.

[cit17] Shachaf Y., Gonen-Wadmany M., Seliktar D. (2010). The biocompatibility of Pluronic® F127 fibrinogen-based hydrogels. Biomaterials.

[cit18] Teodorescu M., Bercea M. (2015). Poly (vinylpyrrolidone)-a versatile polymer for biomedical and beyond medical applications. Polym.-Plast. Technol. Eng..

[cit19] Aung Y.-Y., Kristanti A. N., Lee H. V., Fahmi M. Z. (2021). Boronic-acid-modified nanomaterials for biomedical applications. ACS Omega.

[cit20] Kumar A., Jayeoye T. J., Mohite P., Singh S., Rajput T., Munde S. (2024). *et al.*, Sustainable and consumer-centric nanotechnology-based materials: An update on the multifaceted applications, risks and tremendous opportunities. Nano-Struct. Nano-Objects.

[cit21] Iravani S., Korbekandi H., Mirmohammadi S. V., Zolfaghari B. (2014). Synthesis of silver nanoparticles: chemical, physical and biological methods. Res. Pharm. Sci..

[cit22] Gupta R., Xie H. (2018). Nanoparticles in daily life: applications, toxicity and regulations. J. Environ. Pathol., Toxicol. Oncol..

[cit23] Pansambal S., Oza R., Borgave S., Chauhan A., Bardapurkar P., Vyas S. (2023). *et al.*, Bioengineered cerium oxide (CeO2) nanoparticles and their diverse applications: a review. Appl. Nanosci..

[cit24] Kolahalam L. A., Viswanath I. K., Diwakar B. S., Govindh B., Reddy V., Murthy Y. (2019). Review on nanomaterials: Synthesis and applications. Mater. Today: Proc..

[cit25] Jadoun S., Arif R., Jangid N. K., Meena R. K. (2021). Green synthesis of nanoparticles using plant extracts: A review. Environ. Chem. Lett..

[cit26] Yadi M., Mostafavi E., Saleh B., Davaran S., Aliyeva I., Khalilov R. (2018). *et al.*, Current developments in green synthesis of metallic nanoparticles using plant extracts: a review. Artif. Cells, Nanomed., Biotechnol..

[cit27] Razavi M. , Salahinejad E., Fahmy M., Yazdimamaghani M., Vashaee D., and Tayebi L., Green chemical and biological synthesis of nanoparticles and their biomedical applications, Green Processes for Nanotechnology: from Inorganic to Bioinspired Nanomaterials, 2015, pp. 207–235

[cit28] Rajkumar T., Sapi A., Das G., Debnath T., Ansari A., Patra J. K. (2019). Biosynthesis of silver nanoparticle using extract of Zea mays (corn flour) and investigation of its cytotoxicity effect and radical scavenging potential. J. Photochem. Photobiol., B.

[cit29] Baig N., Kammakakam I., Falath W. (2021). Nanomaterials: A review of synthesis methods, properties, recent progress, and challenges. Mater. Adv..

[cit30] Pechyen C., Tangnorawich B., Toommee S., Marks R., Parcharoen Y. (2024). Green Synthesis of Metal Nanoparticles, Characterization, and Biosensing Applications. Sens. Int..

[cit31] Karataş E. , and Çiner F., Green Synthesis of Nanoparticles and Their Applications, Versatile Approaches to Engineering and Applied Sciences: Materials and Methods, 2023, p. 141

[cit32] Dutta P. P., Bordoloi M., Gogoi K., Roy S., Narzary B., Bhattacharyya D. R. (2017). *et al.*, Antimalarial silver and gold nanoparticles: Green synthesis, characterization and *in vitro* study. Biomed. Pharmacother..

[cit33] Khan M., Tareq F., Hossen M., Roki M. (2018). Green synthesis and characterization of silver nanoparticles using *Coriandrum sativum* leaf extract. J. Eng. Sci. Technol..

[cit34] Velmurugan P., Cho M., Lim S.-S., Seo S.-K., Myung H., Bang K.-S. (2015). *et al.*, Phytosynthesis of silver nanoparticles by Prunus yedoensis leaf extract and their antimicrobial activity. Mater. Lett..

[cit35] Dhand V., Soumya L., Bharadwaj S., Chakra S., Bhatt D., Sreedhar B. (2016). Green synthesis of silver nanoparticles using Coffea arabica seed extract and its antibacterial activity. Mater. Sci. Eng., C.

[cit36] Rostami-Vartooni A., Nasrollahzadeh M., Alizadeh M. (2016). Green synthesis of seashell supported silver nanoparticles using Bunium persicum seeds extract: application of the particles for catalytic reduction of organic dyes. J. Colloid Interface Sci..

[cit37] Farah M. A., Ali M. A., Chen S.-M., Li Y., Al-Hemaid F. M., Abou-Tarboush F. M. (2016). *et al.*, Silver nanoparticles synthesized from Adenium obesum leaf extract induced DNA damage, apoptosis and autophagy via generation of reactive oxygen species. Colloids Surf., B.

[cit38] Chick C. N., Misawa-Suzuki T., Suzuki Y., Usuki T. (2020). Preparation and antioxidant study of silver nanoparticles of Microsorum pteropus methanol extract. Bioorg. Med. Chem. Lett..

[cit39] Ajitha B., Reddy Y. A. K., Reddy P. S. (2015). Green synthesis and characterization of silver nanoparticles using Lantana camara leaf extract. Mater. Sci. Eng., C.

[cit40] Rai M., Bonde S., Golinska P., Trzcińska-Wencel J., Gade A., Abd-Elsalam K. A. (2021). *et al.*, Fusarium as a novel fungus for the synthesis of nanoparticles: mechanism and applications. J. Fungi.

[cit41] Singh T., Jyoti K., Patnaik A., Singh A., Chauhan R., Chandel S. (2017). Biosynthesis, characterization and antibacterial activity of silver nanoparticles using an endophytic fungal supernatant of Raphanus sativus. J. Genet. Eng. Biotechnol..

[cit42] Narayanan K. B., Sakthivel N. (2010). Biological synthesis of metal nanoparticles by microbes. Adv. Colloid Interface Sci..

[cit43] Prema P., Veeramanikandan V., Rameshkumar K., Gatasheh M. K., Hatamleh A. A., Balasubramani R. (2022). *et al.*, Statistical optimization of silver nanoparticle synthesis by green tea extract and its efficacy on colorimetric detection of mercury from industrial waste water. Environ. Res..

[cit44] Ahmed S., Ahmad M., Swami B. L., Ikram S. (2016). Green synthesis of silver nanoparticles using *Azadirachta indica* aqueous leaf extract. J. Radiat. Res. Appl. Sci..

[cit45] Roy P., Das B., Mohanty A., Mohapatra S. (2017). Green synthesis of silver nanoparticles using *Azadirachta indica* leaf extract and its antimicrobial study. Appl. Nanosci..

[cit46] Ongtanasup T., Kamdenlek P., Manaspon C., Eawsakul K. (2024). Green-synthesized silver nanoparticles from *Zingiber officinale* extract: antioxidant potential, biocompatibility, anti-LOX properties, and in silico analysis. BMC Complementary Med. Ther..

[cit47] Al-Kalifawi E. J., Al-Saadi T. M., Al-Dulaimi S. A., Al-Obodi E. E. (2015). Biosynthesis of silver nanoparticles by using onion (*Allium cepa*) extract and study antibacterial activity. J. Genet. Environ. Resour. Conserv..

[cit48] Oda A. M., Abdulkadhim H., Jabuk S. I., Hashim R., Fadhil I., Alaa D. (2019). *et al.*, Green synthesis of silver nanoparticle by cauliflower extract: characterisation and antibacterial activity against storage. IET Nanobiotechnol..

[cit49] Khan M. N., Bashir O., Khan T. A., AL-Thabaiti S. A., Khan Z. (2018). CTAB capped
synthesis of bio-conjugated silver nanoparticles and their enhanced catalytic activities. J. Mol. Liq..

[cit50] Talabani R. F., Hamad S. M., Barzinjy A. A., Demir U. (2021). Biosynthesis of silver nanoparticles and their applications in harvesting sunlight for solar thermal generation. Nanomaterials.

[cit51] Wirwis A., Sadowski Z. (2023). Green synthesis of silver nanoparticles: optimizing green tea leaf extraction for enhanced physicochemical properties. ACS Omega.

[cit52] Hermanto D., Ismillayli N., Fatwa D. H., Zuryati U. K., Muliasari H., Wirawan R. (2024). *et al.*, Bio-mediated electrochemically synthesis of silver nanoparticles using green tea (*Camellia sinensis*) leaves extract and their antibacterial activity. S. Afr. J. Chem. Eng..

[cit53] Ali S. M., Yousef N. M., Nafady N. A. (2015). Application of biosynthesized silver nanoparticles for the control of land snail Eobania vermiculata and some plant pathogenic fungi. J. Nanomater..

[cit54] Wasilewska A., Klekotka U., Zambrzycka M., Zambrowski G., Święcicka I., Kalska-Szostko B. (2023). Physico-chemical properties and antimicrobial activity of silver nanoparticles fabricated by green synthesis. Food Chem..

[cit55] Castillo-López D., Pal U. (2014). Green synthesis of Au nanoparticles using potato extract: stability and growth mechanism. J. Nanopart. Res..

[cit56] Khalilzadeh M. A., Borzoo M. (2016). Green synthesis of silver nanoparticles using onion extract and their application for the preparation of a modified electrode for determination of ascorbic acid. J. Food Drug Anal..

[cit57] Anis S. N. S., Liew W. C., Marsin A. M., Muhamad I. I., Teh S. H., Khudzari A. Z. M. (2023). Microwave-assisted green synthesis of silver nanoparticles using pineapple leaves waste. Clean. Eng. Technol..

[cit58] Siemieniec J., Kruk P. (2013). Synteza nanocząstek srebra oraz złota metodami zielonej chemii. Chemik.

[cit59] Kaviya S., Santhanalakshmi J., Viswanathan B., Muthumary J., Srinivasan K. (2011). Biosynthesis of silver nanoparticles using *Citrus sinensis* peel extract and its antibacterial activity. Spectrochim. Acta, Part A.

[cit60] Jabbar A. H., Al-janabi H. S. O., Hamzah M. Q., Mezan S. O., Tumah A. N., Ameruddin A. S. B. (2020). *et al.*, Green synthesis and characterization of silver nanoparticle (AgNPs) using *Pandanus atrocarpus* extract. Int. J. Adv. Sci. Technol..

[cit61] Ahmadi F., Lackner M. (2024). Green Synthesis of Silver Nanoparticles from *Cannabis Sativa*: Properties, Synthesis, Mechanistic Aspects, and Applications. ChemEngineering.

[cit62] Shirsul J., Tripathi A., Ankamwar B. (2024). Green Biosynthesis of Silver Nanoparticles Utilizing *Monstera deliciosa* Leaf Extract and Estimation of its Antimicrobial Characteristics. Part. Part. Syst. Charact..

[cit63] Mahiuddin M., Saha P., Ochiai B. (2020). Green synthesis and catalytic activity of silver nanoparticles based on *Piper chaba* stem extracts. Nanomaterials.

[cit64] Kusumahastuti D. K., Cahyanti M. N., Aminu N. R. (2024). Green Synthesis of Silver Nanoparticles with Snake Fruit Peel Extract: A Preliminary Study For Optimization of The Preparation Technique. Jur. Penelit. Pendidik. IPA.

[cit65] Balamurugan V., Ragavendran C., Arulbalachandran D., Alrefaei A. F., Rajendran R. (2024). Green synthesis of silver nanoparticles using *Pandanus tectorius* aerial root extract: Characterization, antibacterial, cytotoxic, and photocatalytic properties, and ecotoxicological assessment. Inorg. Chem. Commun..

[cit66] Ahn E.-Y., Park Y. (2020). Anticancer prospects of silver nanoparticles green-synthesized by plant extracts. Mater. Sci. Eng., C.

[cit67] Pungle R., Nile S. H., Makwana N., Singh R., Singh R. P., Kharat A. S. (2022). Green synthesis of silver nanoparticles using the *Tridax Procumbens* plant extract and screening of its antimicrobial and anticancer activities. Oxid. Med. Cell. Longevity.

[cit68] Abdullah M. A. H., Aziz A. H. A., Engliman N. S. (2024). Comparison Study Between Hydrothermal And Coprecipitation Method For Green Synthesize Of Magnetic Silver Nanoparticles. Chem. Nat. Resour. Eng. J..

[cit69] Okaiyeto K., Hoppe H., Okoh A. I. (2021). Plant-based synthesis of silver nanoparticles using aqueous leaf extract of *Salvia officinalis*: characterization and its antiplasmodial activity. J. Cluster Sci..

[cit70] Tesfaye M., Gonfa Y., Tadesse G., Temesgen T., Periyasamy S. (2023). Green synthesis of silver nanoparticles using *Vernonia amygdalina* plant extract and its antimicrobial activities. Heliyon.

[cit71] Birusanti A. B., Mallavarapu U., Nayakanti D., Espenti C. S., Mala S. (2019). Sustainable green synthesis of silver nanoparticles by using *Rangoon creeper* leaves extract and their spectral analysis and anti-bacterial studies. IET Nanobiotechnol..

[cit72] Oves M., Rauf M. A., Aslam M., Qari H. A., Sonbol H., Ahmad I. (2022). *et al.*, Green synthesis of silver nanoparticles by *Conocarpus Lancifolius* plant extract and their antimicrobial and anticancer activities. Saudi J. Biol. Sci..

[cit73] Khatun M., Khatun Z., Karim M. R., Habib M. R., Rahman M. H., Aziz M. A. (2023). Green synthesis of silver nanoparticles using extracts of *Mikania cordata* leaves and evaluation of their antioxidant, antimicrobial and cytotoxic properties. Food Chem. Adv..

[cit74] Metwaly F.-E. M., Moghazy M. A., Sheded M. G., Mohamed A. A. (2024). Green synthesis of silver nanoparticles using leaf extract of the hydrophyte *Persicaria senegalensis*: Preparation and antioxidant activity. Inorg. Nano-Met. Chem..

[cit75] Maghimaa M., Alharbi S. A. (2020). Green synthesis of silver nanoparticles from *Curcuma longa* L. and coating on the cotton fabrics for antimicrobial applications and wound healing activity. J. Photochem. Photobiol., B.

[cit76] Hawar S. N., Al-Shmgani H. S., Al-Kubaisi Z. A., Sulaiman G. M., Dewir Y. H., Rikisahedew J. J. (2022). Green synthesis of silver nanoparticles from *Alhagi graecorum* leaf extract and evaluation of their cytotoxicity and antifungal activity. J. Nanomater..

[cit77] Vijayan R., Joseph S., Mathew B. (2019). Green synthesis of silver nanoparticles using *Nervalia zeylanica* leaf extract and evaluation of their antioxidant, catalytic, and antimicrobial potentials. Part. Sci. Technol..

[cit78] Marta F., Jiří K. (2024). Biosynthesis of Silver Nanoparticles from *Reynoutria bohemica* by Hot and Cold Route. Waste Biomass Valorization.

[cit79] Bhusal M., Pathak I., Bhadel A., Shrestha D. K., Sharma K. R. (2024). Synthesis of silver nanoparticles assisted by aqueous root and leaf extracts of *Rhus chinensis* Mill and its antibacterial activity. Heliyon.

[cit80] Al-Sahli S. A., Al-Otibi F., Alharbi R. I., Amina M., Al Musayeib N. M. (2024). Silver nanoparticles improve the fungicidal properties of *Rhazya stricta* decne aqueous extract against plant pathogens. Sci. Rep..

[cit81] Purohit A., Sharma R., Shiv Ramakrishnan R., Sharma S., Kumar A., Jain D. (2022). *et al.*, Biogenic synthesis of silver nanoparticles (AgNPs) using aqueous leaf extract of *Buchanania lanzan spreng* and evaluation of their antifungal activity against phytopathogenic fungi. Bioinorg. Chem. Appl..

[cit82] Liem L. N., The N. P., Nguyen D. (2019). Microwave assisted green synthesis of silver nanoparticles using Mulberry leaves extract and silver nitrate solution. Technologies.

[cit83] Jalasutram J., Nowduri A., Kumari P. P., Shaik T. (2024). First-Time Investigation of *Bombax ceiba* Stem Crude Extract's Role for Autoclave-assisted Silver Nanoparticle Synthesis from Silver Sulphate and Silver Nitrate: Multifaceted Characterization, Catalytic and Antibacterial Investigations. Moroccan J. Chem..

[cit84] Viswanathan S., Palaniyandi T., Shanmugam R., Karunakaran S., Pandi M., Wahab M. R. A. (2024). *et al.*, Synthesis, characterization, cytotoxicity, and antimicrobial studies of green synthesized silver nanoparticles using red seaweed *Champia parvula*. Biomass Convers. Biorefin..

[cit85] Jalilian F., Chahardoli A., Sadrjavadi K., Fattahi A., Shokoohinia Y. (2020). Green synthesized silver nanoparticle from *Allium ampeloprasum* aqueous extract: Characterization, antioxidant activities, antibacterial and cytotoxicity effects. Adv. Powder Technol..

[cit86] Green Synthesis Reviews: Synthesis of Silver Nanoparticles from Galangal Rhizome Extract (Alpina galanga) and Centella asiatica Leaves as Bacterial Inhibitors, ed. Proceeding International Conference on Religion, Science and Education, A. D. Rizkita, F. H. Syarif, N. R. Al'masum, T. E. J. Gulo, and S. B. Shapira, 2023

[cit87] Almatroudi A. (2020). Silver nanoparticles: Synthesis, characterisation and biomedical applications. Open Life Sci..

[cit88] Anuradha G., SyamaSundar B., Ramana M. (2014). Ocimum americanum L. leaf extract mediated synthesis of silver nanoparticles: A novel approach towards weed utilization. Arch. Appl. Sci. Res..

[cit89] Mallikarjuna K., Narasimha G., Dillip G., Praveen B., Shreedhar B., Lakshmi C. S. (2011). *et al.*, Green synthesis of silver nanoparticles using Ocimum leaf extract and their characterization. Dig. J. Nanomater. Biostruct..

[cit90] Anju T., Parvathy S., Veettil M. V., Rosemary J., Ansalna T., Shahzabanu M. (2021). *et al.*, Green synthesis of silver nanoparticles from *Aloe vera* leaf extract and its antimicrobial activity. Mater. Today: Proc..

[cit91] Abdel-Wahab B. A., Haque A., Alotaibi H. F., Alasiri A. S., Elnoubi O. A., Ahmad M. Z. (2024). *et al.*, Eco-friendly green synthesis of silver nanoparticles utilizing olive oil waste by-product and their incorporation into a chitosan-*Aloe vera* gel composite for enhanced wound healing in acid burn injuries. Inorg. Chem. Commun..

[cit92] Ansari M., Ahmed S., Abbasi A., Khan M. T., Subhan M., Bukhari N. A. (2023). *et al.*, Plant mediated fabrication of silver nanoparticles, process optimization, and impact on tomato plant. Sci. Rep..

[cit93] Sanchez-Salvador J. L., Campano C., Negro C., Monte M. C., Blanco A. (2021). Increasing the Possibilities of TEMPO-Mediated Oxidation in the Production of Cellulose Nanofibers by Reducing the Reaction Time and Reusing the Reaction Medium. Adv. Sustainable Syst..

[cit94] Iravani S. (2011). Green synthesis of metal nanoparticles using plants. Green Chem..

[cit95] Jain D., Daima H. K., Kachhwaha S., Kothari S. (2009). Synthesis of plant-mediated silver nanoparticles using papaya fruit extract and evaluation of their anti microbial activities. Dig. J. Nanomater. Biostruct..

[cit96] Moradi F., Sedaghat S., Moradi O., Arab Salmanabadi S. (2021). Review on green nano-biosynthesis of silver nanoparticles and their biological activities: With an emphasis on medicinal plants. Inorg. Nano-Met. Chem..

[cit97] Nie S., Liu C., Zhang Z., Liu Y. (2016). Nitric acid-mediated shape-controlled synthesis and catalytic activity of silver hierarchical microcrystals. RSC Adv..

[cit98] Raza M. A., Kanwal Z., Rauf A., Sabri A. N., Riaz S., Naseem S. (2016). Size-and shape-dependent antibacterial studies of silver nanoparticles synthesized by wet chemical routes. Nanomaterials.

[cit99] Marin S., Mihail Vlasceanu G., Elena Tiplea R., Raluca Bucur I., Lemnaru M., Minodora Marin M. (2015). *et al.*, Applications and toxicity of silver nanoparticles: a recent review. Curr. Top. Med. Chem..

[cit100] Wei L., Lu J., Xu H., Patel A., Chen Z.-S., Chen G. (2015). Silver nanoparticles: synthesis, properties, and therapeutic applications. Drug discovery today.

[cit101] Menichetti A., Mavridi-Printezi A., Mordini D., Montalti M. (2023). Effect of size, shape and surface functionalization on the antibacterial activity of silver nanoparticles. J. Funct. Biomater..

[cit102] Thoms S., Gonsalves R. A., Jose J., Zyoud S. H., Prasad A. R., Garvasis J. (2024). Plant-Based Synthesis, characterization Approaches, Applications and Toxicity of Silver Nanoparticles: A Comprehensive Review. J. Biotechnol..

[cit103] Wang L., Zhang T., Li P., Huang W., Tang J., Wang P. (2015). *et al.*, Use of synchrotron radiation-analytical techniques to reveal chemical origin of silver-nanoparticle cytotoxicity. ACS Nano.

[cit104] Tortella G., Rubilar O., Durán N., Diez M., Martínez M., Parada J. (2020). *et al.*, Silver nanoparticles: Toxicity in model organisms as an overview of its hazard for human health and the environment. J. Hazard. Mater..

[cit105] Choi Y., Kim H.-A., Kim K.-W., Lee B.-T. (2018). Comparative toxicity of silver nanoparticles and silver ions to Escherichia coli. J. Environ. Sci..

[cit106] Salem J. K., El-Nahhal I. M., Najri B. A., Hammad T. M. (2016). Utilization of surface Plasmon resonance band of silver nanoparticles for determination of critical micelle concentration of cationic surfactants. Chem. Phys. Lett..

[cit107] Shrivas K., Sahu S., Patra G. K., Jaiswal N. K., Shankar R. (2016). Localized surface plasmon resonance of silver nanoparticles for sensitive colorimetric detection of chromium in surface water, industrial waste water and vegetable samples. Anal. Methods.

[cit108] Yu C.-C., Chou S.-Y., Tseng Y.-C., Tseng S.-C., Yen Y.-T., Chen H.-L. (2015). Single-shot laser treatment provides quasi-three-dimensional paper-based substrates for SERS with attomolar sensitivity. Nanoscale.

[cit109] Duman H., Eker F., Akdaşçi E., Witkowska A. M., Bechelany M., Karav S. (2024). Silver nanoparticles: A comprehensive review of synthesis methods and chemical and physical properties. Nanomaterials.

[cit110] Salayová A., Bedlovičová Z., Daneu N., Baláž M., Lukáčová Bujňáková Z., Balážová Ľ. (2021). *et al.*, Green synthesis of silver nanoparticles with antibacterial activity using various medicinal plant extracts: Morphology and antibacterial efficacy. Nanomaterials.

[cit111] Ongtanasup T., Prommee N., Jampa O., Limcharoen T., Wanmasae S., Nissapatorn V. (2022). *et al.*, The cholesterol-modulating effect of the new herbal medicinal recipe from yellow vine (Coscinium fenestratum (Goetgh.)), ginger (*Zingiber officinale* Roscoe.), and safflower (Carthamus tinctorius L.) on suppressing PCSK9 expression to upregulate LDLR expression in HepG2 cells. Plants.

[cit112] Gupta V., Kannan K., Tari V., Chandra N. (2024). Bioengineered silver nanoparticles using *Brassica oleracea* sub sp. botrytis (L.) for enhanced antibacterial activity. Phys. Chem. Solid State.

[cit113] Rolim W. R., Pelegrino M. T., de Araújo Lima B., Ferraz L. S., Costa F. N., Bernardes J. S. (2019). *et al.*, Green tea extract mediated biogenic synthesis of silver nanoparticles: Characterization, cytotoxicity evaluation and antibacterial activity. Appl. Surf. Sci..

[cit114] Maryani F., Septama A. W. (2022). Microwave-assisted green synthesis of Desmodium triquetrum-mediated silver nanoparticles: enhanced antibacterial, antibiofilm, and cytotoxicity activities against human breast cancer cell lines. Mater. Adv..

[cit115] Gopinath V., MubarakAli D., Priyadarshini S., Priyadharsshini N. M., Thajuddin N., Velusamy P. (2012). Biosynthesis of silver nanoparticles from Tribulus terrestris and its antimicrobial activity: a novel biological approach. Colloids Surf., B.

[cit116] Velmurugan G., Chohan J. S., Paramasivam P., Maranan R. (2024). Green synthesis of silver nanoparticles from southern *Eucalyptus globulus*: Potent antioxidants and photocatalysts for rhodamine B dye degradation. Desalin. Water Treat..

[cit117] Yontar A. K., Çevik S. (2023). Bio-Synthesized Silver Nanoparticles Using *Cannabis Sativa* Seed Extracts and Its Anticancer Effects. Plasmonics.

[cit118] Knyazev Y. V., Balaev D. A., Yaroslavtsev R. N., Krasikov A. A., Velikanov D. A., Mikhlin Y. L. (2022). *et al.*, Tuning of the Interparticle interactions in ultrafine ferrihydrite nanoparticles. Adv. Nan. Res..

[cit119] Balamurugan V., Ragavendran C., Arulbalachandran D. (2024). Eco-friendly green synthesis of AgNPs from Elaeocarpus serratus fruit extract: potential to antibacterial, antioxidant, cytotoxic effects of colon cancerous cells (HT-29) and its toxicity assessments of marine microcrustacean Artemia nauplii. Mol. Biol. Rep..

[cit120] Vasyliev G., Vorobyova V. (2020). Valorization of Food Waste to Produce Eco-Friendly Means of Corrosion Protection and “Green” Synthesis of Nanoparticles. Adv. Mater. Sci. Eng..

[cit121] Kavimani V., Divakaran D., Sriariyanun M., Suganya Priyadharshini G., Gopal P., Suyambulingam I. (2023). *et al.*, Facile exfoliation and physicochemical characterization of biomass-based cellulose derived from *Pandanus tectorius* leaves for sustainable environment. Biomass Convers. Biorefin..

[cit122] Walker J. R., Lintott E. A. (1997). A phytochemical register of New Zealand lichens. N. Z. J. Bot..

[cit123] Sengupta A., Sarkar A. (2022). Synthesis and characterization of nanoparticles from neem leaves and banana peels: a green prospect for dye degradation in wastewater. Ecotoxicology.

[cit124] Emam M., Soliman M. M., Eisa W. H., Hasanin M. (2022). Solid and liquid green Ag nanoparticles based on banana peel extract as an eco-friendly remedy for ringworm in pets. Surf. Interface Anal..

[cit125] Saxena M., Saxena J., Nema R., Singh D., Gupta A. (2013). Phytochemistry of medicinal plants. J. Pharmacogn. Phytochem..

[cit126] Nicolescu C. M., Olteanu R. L., Bumbac M. (2017). Growth dynamics study of silver nanoparticles obtained by green synthesis using *Salvia officinalis* extract. Anal. Lett..

[cit127] Bumbac M., Olteanu R. L., Ion R. M., Nicolescu C. M. (2018). Influence of Temperature on the Growth of Silver Nanoparticles Synthesized Using *Salvia officinalis* Aqueous Extract. Spectroscopy.

[cit128] Mandal J. (2013). *In vitro* regeneration of *Rangoon creeper* (Quisqualis indica Linn.). Indian J. Biotechnol..

[cit129] Al-Taweel A. M., Perveen S., Fawzy G. A., Mehmood R., Khan A., Khan S. I. (2016). New ellagic acid derivative from the fruits of heat-tolerant plant *Conocarpus lancifolius* Engl, and their anti-inflammatory, cytotoxic, PPAR agonistic activities. Pak. J. Pharm. Sci..

[cit130] Siddiqui S. A., Rahman A., Rahman M. O., Akbar M. A., Ali M. A., Al-Hemaid F. M. (2019). *et al.*, A novel triterpenoid 16-hydroxy betulinic acid isolated from *Mikania cordata* attributes multi-faced pharmacological activities. Saudi J. Biol. Sci..

[cit131] Lall N. , Cuyler M., and Payne B. D., Cosmeceutical Products from Indigenous South African Wetland Plants, 2024

[cit132] Hu X., Wu L., Du M., Wang L. (2022). Eco-friendly synthesis of size-controlled silver nanoparticles by using *Areca catechu* nut aqueous extract and investigation of their potent antioxidant and anti-bacterial activities. Arabian J. Chem..

[cit133] Amudhan M. S., Begum V. H., Hebbar K. (2012). A review on phytochemical and pharmacological potential of *Areca catechu* L. seed. Int. J. Pharm. Sci. Res..

[cit134] Van N. T. H., Riviere C., Long P. Q., Vien T. A., Van Kiem P., Van Minh C. (2015). Flavonoids, megastigmanes and other constituents from Ardisia incarnata. Biochem. Syst. Ecol..

[cit135] Vishwanath R., Negi B. (2021). Conventional and green methods of synthesis of silver nanoparticles and their antimicrobial properties. Curr. Res. Green Sustainable Chem..

[cit136] Baruah D., Yadav R. N. S., Yadav A., Das A. M. (2019). Alpinia nigra fruits mediated synthesis of silver nanoparticles and their antimicrobial and photocatalytic activities. J. Photochem. Photobiol., B.

[cit137] Maurya S. K. , Verma N., and Verma D. K., Current Research Journal of Pharmaceutical and Allied Sciences, 2018

[cit138] Lim T. , and Lim T., *Allium ampeloprasum*. Edible Medicinal and Non Medicinal Plants: Volume 9, Modified Stems, Roots, Bulbs, 2015, pp. 103–123

[cit139] Mlalila N. G., Swai H. S., Hilonga A., Kadam D. M. (2016). Antimicrobial dependence of silver nanoparticles on surface plasmon resonance bands against Escherichia coli. Nanotechnol., Sci. Appl..

[cit140] Meena K., Singh S., Bharti A. (2014). Vijay. J. Mater. Sci.: Mater. Electron..

[cit141] Khodadadi B., Bordbar M., Nasrollahzadeh M. (2017). Achillea millefolium L. extract mediated green synthesis of waste peach kernel shell supported silver nanoparticles: application of the nanoparticles for catalytic reduction of a variety of dyes in water. J. Colloid Interface Sci..

[cit142] Khodadadi B., Bordbar M., Nasrollahzadeh M. (2017). Green synthesis of Pd nanoparticles at Apricot kernel shell substrate using Salvia hydrangea extract: catalytic activity for reduction of organic dyes. J. Colloid Interface Sci..

[cit143] Edison T. N. J. I., Lee Y. R., Sethuraman M. G. (2016). Green synthesis of silver nanoparticles using Terminalia cuneata and its catalytic action in reduction of direct yellow-12 dye. Spectrochim. Acta, Part A.

[cit144] Wang J., Liu J., Guo X., Yan L., Lincoln S. F. (2016). The formation and catalytic activity of silver nanoparticles in aqueous polyacrylate solutions. Front. Chem. Sci. Eng..

[cit145] Ghosh B. K., Hazra S., Naik B., Ghosh N. N. (2015). Preparation of Cu nanoparticle loaded SBA-15 and their excellent catalytic activity in reduction of variety of dyes. Powder Technol..

[cit146] Shah D., Kaur H. (2014). Resin-trapped gold nanoparticles: An efficient catalyst for reduction of nitro compounds and Suzuki-Miyaura coupling. J. Mol. Catal. A: Chem..

[cit147] Atarod M., Nasrollahzadeh M., Sajadi S. M. (2016). Green synthesis of Pd/RGO/Fe3O4 nanocomposite using Withania coagulans leaf extract and its application as magnetically separable and reusable catalyst for the reduction of 4-nitrophenol. J. Colloid Interface Sci..

[cit148] Bordbar M., Alimohammadi T., Khoshnevisan B., Khodadadi B., Yeganeh-Faal A. (2015). Preparation of MWCNT/TiO_2_–Co nanocomposite electrode by electrophoretic deposition and electrochemical study of hydrogen storage. Int. J. Hydrogen Energy.

[cit149] Zhang Y., Zhu P., Chen L., Li G., Zhou F., Lu D. D. (2014). *et al.*, Hierarchical architectures of monodisperse porous Cu microspheres: synthesis, growth mechanism, high-efficiency and recyclable catalytic performance. J. Mater. Chem. A.

[cit150] Vidhu V., Philip D. (2014). Catalytic degradation of organic dyes using biosynthesized silver nanoparticles. Micron.

[cit151] Nie P., Zhao Y., Xu H. (2023). Synthesis, applications, toxicity and toxicity mechanisms of silver nanoparticles: A review. Ecotoxicol. Environ. Saf..

[cit152] Khan S. A., Shahid S., Lee C.-S. (2020). Green synthesis of gold and silver nanoparticles using leaf extract of Clerodendrum inerme; characterization, antimicrobial, and antioxidant activities. Biomolecules.

[cit153] Alshehri A. A., Malik M. A. (2020). Phytomediated photo-induced green synthesis of silver nanoparticles using Matricaria chamomilla L. and its catalytic activity against rhodamine B. Biomolecules.

[cit154] Singh R., Hano C., Nath G., Sharma B. (2021). Green biosynthesis of silver nanoparticles using leaf extract of Carissa carandas L. and their antioxidant and antimicrobial activity against human pathogenic bacteria. Biomolecules.

[cit155] Wahid I., Kumari S., Ahmad R., Hussain S. J., Alamri S., Siddiqui M. H. (2020). *et al.*, Silver nanoparticle regulates salt tolerance in wheat through changes in ABA concentration, ion homeostasis, and defense systems. Biomolecules.

[cit156] Silva Viana R. L., Pereira F. G., Jane Campos Medeiros M., Antonio Morgano M., Gabriela Chagas Faustino Alves M., Domingues P. L. F. (2020). *et al.*, Green synthesis of antileishmanial and antifungal silver nanoparticles using corn cob xylan as a reducing and stabilizing agent. Biomolecules.

[cit157] Mickymaray S. (2019). One-step synthesis of silver nanoparticles using Saudi Arabian desert seasonal plant Sisymbrium irio and antibacterial activity against multidrug-resistant bacterial strains. Biomolecules.

[cit158] Bawazeer S., Rauf A., Shah S. U. A., Shawky A. M., Al-Awthan Y. S., Bahattab O. S. (2021). *et al.*, Green synthesis of silver nanoparticles using Tropaeolum majus: Phytochemical screening and antibacterial studies. Green Process. Synth..

[cit159] Patil M. P., Kim G.-D. (2017). Eco-friendly approach for nanoparticles synthesis and mechanism behind antibacterial activity of silver and anticancer activity of gold nanoparticles. Appl. Microbiol. Biotechnol..

[cit160] Yaqoob A. A., Umar K., Ibrahim M. N. M. (2020). Silver nanoparticles: various methods of synthesis, size affecting factors and their potential applications–a review. Appl. Nanosci..

[cit161] Shanmuganathan R., Karuppusamy I., Saravanan M., Muthukumar H., Ponnuchamy K., Ramkumar V. S. (2019). *et al.*, Synthesis of silver nanoparticles and their biomedical applications-a comprehensive review. Curr. Pharm. Des..

[cit162] Suriati G., Mariatti M., Azizan A. (2014). Synthesis of silver nanoparticles by chemical reduction method: Effect of reducing agent and surfactant concentration. Int. J. Automot. Mech. Eng..

[cit163] Khatoon U. T., Rao G. N., Mohan K. M., Ramanaviciene A., Ramanavicius A. (2017). Antibacterial and antifungal activity of silver nanospheres synthesized by tri-sodium citrate assisted chemical approach. Vacuum.

[cit164] Sholikhah U., Pujiyanto A., Lestari E., Sarmini E., Lubis H. (2018). Critical parameters of silver nanoparticles (AgNPs) synthesized by sodium borohydride reduction. Res. J. Chem. Environ..

[cit165] Elgawady Y., Ponnamma D., Hassan M. K., Adham S., Karim A., Al-Maadeed M. A. A. (2022). In situ synthesized amphiphilic polysulfone-poly (ethylene-glycol) block copolymer/silver nanocomposite for separating oil/water emulsion. J. Appl. Polym. Sci..

[cit166] Sotiriou G. A., Teleki A., Camenzind A., Krumeich F., Meyer A., Panke S. (2011). *et al.*, Nanosilver on nanostructured
silica: Antibacterial activity and Ag surface area. Chem. Eng. J..

[cit167] Pradeep M., Kruszka D., Kachlicki P., Mondal D., Franklin G. (2021). Uncovering the phytochemical basis and the mechanism of plant extract-mediated eco-friendly synthesis of silver nanoparticles using ultra-performance liquid chromatography coupled with a photodiode array and high-resolution mass spectrometry. ACS Sustain. Chem. Eng..

[cit168] Quintero-Quiroz C., Acevedo N., Zapata-Giraldo J., Botero L. E., Quintero J., Zárate-Triviño D. (2019). *et al.*, Optimization of silver nanoparticle synthesis by chemical reduction and evaluation of its antimicrobial and toxic activity. Biomater. Res..

[cit169] Sportelli M. C., Izzi M., Volpe A., Clemente M., Picca R. A., Ancona A. (2018). *et al.*, The pros and cons of the use of laser ablation synthesis for the production of silver nano-antimicrobials. Antibiotics.

[cit170] Jeevanandam J., Chan Y. S., Danquah M. K. (2016). Biosynthesis of metal and metal oxide nanoparticles. ChemBioEng Rev..

[cit171] Saratale R. G., Shin H. S., Kumar G., Benelli G., Kim D.-S., Saratale G. D. (2018). Exploiting antidiabetic activity of silver nanoparticles synthesized using Punica granatum leaves and anticancer potential against human liver cancer cells (HepG2). Artificial cells, nanomedicine, and biotechnology.

[cit172] Abbasi B. H., Nazir M., Muhammad W., Hashmi S. S., Abbasi R., Rahman L. (2019). *et al.*, A comparative evaluation of the antiproliferative activity against Hepg2 liver carcinoma cells of plant-derived silver nanoparticles from basil extracts with contrasting anthocyanin contents. Biomolecules.

[cit173] Mohanpuria P., Rana N. K., Yadav S. K. (2008). Biosynthesis of nanoparticles: technological concepts and future applications. J. Nanopart. Res..

[cit174] Sharma N. C., Sahi S. V., Nath S., Parsons J. G., Gardea-Torresde J. L., Pal T. (2007). Synthesis of plant-mediated gold nanoparticles and catalytic role of biomatrix-embedded nanomaterials. Environ. Sci. Technol..

[cit175] Chandran S. P., Chaudhary M., Pasricha R., Ahmad A., Sastry M. (2006). Synthesis of gold nanotriangles and silver nanoparticles using Aloevera plant extract. Biotechnol. Prog..

[cit176] Saxena A., Tripathi R., Zafar F., Singh P. (2012). Green synthesis of silver nanoparticles using aqueous solution of Ficus benghalensis leaf extract and characterization of their antibacterial activity. Mater. Lett..

[cit177] Mikhailova E. O. (2020). Silver nanoparticles: Mechanism of action and probable bio-application. J. Funct. Biomater..

[cit178] Shankar S. S., Rai A., Ahmad A., Sastry M. (2004). Rapid synthesis of Au, Ag, and bimetallic Au core–Ag shell nanoparticles using Neem (*Azadirachta indica*) leaf broth. J. Colloid Interface Sci..

[cit179] Li S., Shen Y., Xie A., Yu X., Qiu L., Zhang L. (2007). *et al.*, Green synthesis of silver nanoparticles using *Capsicum annuum* L. extract. Green Chem..

[cit180] Tripathi R. M., Ranac D., Shrivastav A., Singh R. P., Shrivastav B. R. (2013). Biogenic Synthesis of Silver Nanoparticles Using Saraca indica Leaf Extract and Evaluation of Their Antibacterial Activity. Nano Biomed. Eng..

[cit181] Shaikh W. A., Chakraborty S., Owens G., Islam R. U. (2021). A review of the phytochemical mediated synthesis of AgNP (silver nanoparticle): The wonder particle of the past decade. Appl. Nanosci..

[cit182] Jain S., Mehata M. S. (2017). Medicinal plant leaf extract and pure flavonoid mediated green
synthesis of silver nanoparticles and their enhanced antibacterial property. Sci. Rep..

[cit183] Gurunathan S., Han J. W., Kwon D.-N., Kim J.-H. (2014). Enhanced antibacterial and anti-biofilm activities of silver nanoparticles against Gram-negative and Gram-positive bacteria. Nanoscale Res. Lett..

[cit184] Ovais M., Khalil A. T., Islam N. U., Ahmad I., Ayaz M., Saravanan M. (2018). *et al.*, Role of plant phytochemicals and microbial enzymes in biosynthesis of metallic nanoparticles. Appl. Microbiol. Biotechnol..

[cit185] Kampan N. C., Madondo M. T., McNally O. M., Quinn M., Plebanski M. (2015). Paclitaxel and its evolving role in the management of ovarian cancer. BioMed Res. Int..

[cit186] Schläger S., Dräger B. (2016). Exploiting plant alkaloids. Curr. Opin. Biotechnol..

[cit187] James W. O. (1953). Alkaloid formation in plants. J. Pharm. Pharmacol..

[cit188] Prabhu N., Raj D. T., Yamuna G. K., Ayisha S. S., Puspha J., Innocent D. (2010). Synthesis of silver phyto nanoparticles and their antibacterial efficacy. Dig. J. Nanomater. Biostruct..

[cit189] Njagi E. C., Huang H., Stafford L., Genuino H., Galindo H. M., Collins J. B. (2011). *et al.*, Biosynthesis of iron and silver nanoparticles at room temperature using aqueous sorghum bran extracts. Langmuir.

[cit190] Tamulevičienė A., Puišo J., Prosyčevas I., Tamulevičius S. (2009). Investigation of silver nanoparticles formation kinetics during reduction of silver nitrate with sodium citrate. Mater. Sci..

[cit191] Hasnain M. S., Javed M. N., Alam M. S., Rishishwar P., Rishishwar S., Ali S. (2019). *et al.*, Purple heart plant leaves extract-mediated silver nanoparticle synthesis: Optimization by Box-Behnken design. Mater. Sci. Eng., C.

[cit192] Khan S., Singh S., Gaikwad S., Nawani N., Junnarkar M., Pawar S. V. (2020). Optimization of process parameters for the synthesis of silver nanoparticles from Piper betle leaf aqueous extract, and evaluation of their antiphytofungal activity. Environ. Sci. Pollut. Res..

[cit193] Ćwik M., Buczyńska D., Sulowska K., Roźniecka E., Mackowski S., Niedziółka-Jönsson J. (2019). Optical properties of submillimeter silver nanowires synthesized using the hydrothermal method. Materials.

[cit194] Kvítek L., Panáček A., Soukupová J., Kolář M., Večeřová R., Prucek R. (2008). *et al.*, Effect of surfactants and polymers on stability and antibacterial activity of silver nanoparticles (NPs). J. Phys. Chem. C.

[cit195] Sharma V. K., Yngard R. A., Lin Y. (2009). Silver nanoparticles: green synthesis and their antimicrobial activities. Adv. Colloid Interface Sci..

[cit196] Fatimah I. (2016). Green synthesis of silver nanoparticles using extract of Parkia speciosa Hassk pods assisted by microwave irradiation. J. Adv. Res..

[cit197] Dwivedi A. D., Gopal K. (2010). Biosynthesis of silver and gold nanoparticles using Chenopodium album leaf extract. Colloids Surf., A.

[cit198] Anandachockalingam A., Shanmugam R., Ryntathiang I., Jothinathan M. K. D. (2024). Green Synthesis of Silver Nanoparticles Using *Zingiber officinale* and Ocimum gratissimum Formulation for Its Anti-inflammatory and Antidiabetic Activity: An *In Vitro* Study. Cureus.

[cit199] Kadam J., Dhawal P., Barve S., Kakodkar S. (2020). Green
synthesis of silver nanoparticles using cauliflower waste and their multifaceted applications in photocatalytic degradation of methylene blue dye and Hg 2+ biosensing. SN Appl. Sci..

[cit200] Pram P. , Mishra N., Vaithilingam M., Samuel M. K., Mohanan M., Kothari N., and et al., Green Synthesis of Silver Nanoparticles using *Coriandrum sativum* and Murraya koenigii Leaf Extract and its Thrombolytic Activity. Cardiovascular & Hematological Agents in Medicinal Chemistry (Formerly Current Medicinal Chemistry-Cardiovascular & Hematological Agents), 2024, vol. 22, (2), pp. 230–239 10.2174/0118715257279159240118050207 38975619

[cit201] Elattar K. M., Ghoniem A. A., Al-Otibi F. O., El-Hersh M. S., Helmy Y. A., Saber W. I. (2023). Phytogenic synthesis and characterization of silver metallic/bimetallic nanoparticles using *Beta vulgaris* L. extract and assessments of their potential biological activities. Appl. Sci..

[cit202] Azevedo M. M. R. , Bressan C. R., Ferreira L. P., dos Santos Torres C. B., Oliveira Y. V. S., Nakazato G., and et al., Green Synthesis of Silver Nanoparticles From Pineapple (*Ananas Comosus*) Peel Extract: Physical-chemical Characterization and *in Vitro* Biological Activities, 2023

[cit203] Smirnov O., Kalynovskyi V., Zelena P., Yumyna Y., Dzhagan V., Kovalenko M. (2023). *et al.*, Bactericidal activity of Ag nanoparticles biosynthesized from *Capsicum annuum* pericarps against phytopathogenic Clavibacter michiganensis. Sci. Nat..

[cit204] Subhani A. A., Irshad M., Ali S., Jawad M., Akhtar M. F., Summer M. (2024). UV-spectrophotometric optimization of temperature, pH, concentration and time for *eucalyptus globulus* capped silver nanoparticles synthesis, their characterization and evaluation of biological applications. J. Fluoresc..

[cit205] Takcı D. K., Ozdenefe M. S., Genc S. (2023). Green synthesis of silver nanoparticles with an antibacterial activity using *Salvia officinalis* aqueous extract. J. Cryst. Growth.

[cit206] Li J. F., Liu Y.-C., Chokkalingam M., Rupa E. J., Mathiyalagan R., Hurh J. (2020). *et al.*, Phytosynthesis of silver nanoparticles using rhizome extract of Alpinia officinarum and their photocatalytic removal of dye under UV and visible light irradiation. Optik.

[cit207] Raina S., Roy A., Bharadvaja N. (2020). Degradation of dyes using biologically synthesized silver and copper nanoparticles. Environ. Nanotechnol., Monit. Manage..

[cit208] Velmurugan P., Shim J., Kim H., Lim J.-M., Kim S. A., Seo Y.-S. (2020). *et al.*, Bio-functionalization of cotton, silk, and leather using different in-situ silver nanoparticle synthesis modules, and their antibacterial properties. Res. Chem. Intermed..

[cit209] Rashid S., Azeem M., Khan S. A., Shah M. M., Ahmad R. (2019). Characterization and synergistic antibacterial potential of green synthesized silver nanoparticles using aqueous root extracts of important medicinal plants of Pakistan. Colloids Surf., B.

[cit210] de Aragao A. P., de Oliveira T. M., Quelemes P. V., Perfeito M. L. G., Araujo M. C., Santiago JdA. S. (2019). *et al.*, Green synthesis of silver nanoparticles using the seaweed Gracilaria birdiae and their antibacterial activity. Arabian J. Chem..

[cit211] Singh A. K., Tiwari R., Kumar V., Singh P., Khadim S. R., Tiwari A. (2017). *et al.*, Photo-induced biosynthesis of silver nanoparticles from aqueous extract of Dunaliella salina and their anticancer potential. J. Photochem. Photobiol., B.

[cit212] Deshi J. J., Barminas J. T., Onwuka J. C., Dass P. M., Maitera O. N., Muazu I. (2016). Antimicrobial efficacy of biosynthesized silver nanoparticles from different solvent extracts of Waltheria americana root. J. Anal. Sci. Technol..

[cit213] Rajan A., Vilas V., Philip D. (2015). Catalytic and antioxidant properties of biogenic silver nanoparticles synthesized using *Areca catechu* nut. J. Mol. Liq..

[cit214] Suresh G., Gunasekar P. H., Kokila D., Prabhu D., Dinesh D., Ravichandran N. (2014). *et al.*, Green synthesis of silver nanoparticles using Delphinium denudatum root extract exhibits antibacterial and mosquito larvicidal activities. Spectrochim. Acta, Part A.

[cit215] Edison T. J. I., Sethuraman M. (2012). Instant green synthesis of silver nanoparticles using Terminalia chebula fruit extract and evaluation of their catalytic activity on reduction of methylene blue. Process Biochem..

[cit216] Mittal A. K., Chisti Y., Banerjee U. C. (2013). Synthesis of metallic nanoparticles using plant extracts. Biotechnol. Adv..

[cit217] Rajakumar G., Rahuman A. A. (2011). Larvicidal activity of synthesized silver nanoparticles using Eclipta prostrata leaf extract against filariasis and malaria vectors. Acta Trop..

[cit218] Jagtap U. B., Bapat V. A. (2013). Green synthesis of silver nanoparticles using Artocarpus heterophyllus Lam. seed extract and its antibacterial activity. Ind. Crops Prod..

[cit219] Ebrahimzadeh M. A., Naghizadeh A., Amiri O., Shirzadi-Ahodashti M., Mortazavi-Derazkola S. (2020). Green and facile synthesis of Ag nanoparticles using Crataegus pentagyna fruit extract (CP-AgNPs) for organic pollution dyes degradation and antibacterial application. Bioorg. Chem..

[cit220] Ocsoy I., Temiz M., Celik C., Altinsoy B., Yilmaz V., Duman F. (2017). A green approach for formation of silver nanoparticles on magnetic graphene oxide and highly effective antimicrobial activity and reusability. J. Mol. Liq..

[cit221] Saha J., Begum A., Mukherjee A., Kumar S. (2017). A novel green synthesis of silver nanoparticles and their catalytic action in reduction of Methylene Blue dye. Sustainable Environ. Res..

[cit222] Ameen F., Srinivasan P., Selvankumar T., Kamala-Kannan S., Al Nadhari S., Almansob A. (2019). *et al.*, Phytosynthesis of silver nanoparticles using Mangifera indica flower extract as bioreductant and their broad-spectrum antibacterial activity. Bioorg. Chem..

[cit223] Hamedi S., Shojaosadati S. A. (2019). Rapid and green synthesis of silver nanoparticles using Diospyros lotus extract: Evaluation of their biological and catalytic activities. Polyhedron.

[cit224] Swarnavalli G. C. J., Dinakaran S., Raman N., Jegadeesh R., Pereira C. (2017). Bio inspired synthesis of monodispersed silver nano particles using Sapindus emarginatus pericarp extract–Study of antibacterial efficacy. J. Saudi Chem. Soc..

[cit225] Taheri M. M., Kadir M. R. A., Shafiai N. K. A., Shokuhfar T., Assadian M., Shirdar M. R. (2015). Green synthesis of silver nanoneedles using shallot and apricot tree gum. Trans. Nonferrous Met. Soc. China.

[cit226] Rajkuberan C., Prabukumar S., Sathishkumar G., Wilson A., Ravindran K., Sivaramakrishnan S. (2017). Facile synthesis of silver nanoparticles using Euphorbia antiquorum L. latex extract and evaluation of their biomedical perspectives as anticancer agents. J. Saudi Chem. Soc..

[cit227] Mariselvam R., Ranjitsingh A., Nanthini A. U. R., Kalirajan K., Padmalatha C., Selvakumar P. M. (2014). Green synthesis of silver nanoparticles from the extract of the inflorescence of Cocos nucifera (Family: Arecaceae) for enhanced antibacterial activity. Spectrochim. Acta, Part A.

[cit228] Ibrahim H. M. (2015). Green synthesis and characterization of silver nanoparticles using banana peel extract and their antimicrobial activity against representative microorganisms. J. Radiat. Res. Appl. Sci..

[cit229] Natarajan K., Selvaraj S., Ramachandra M. (2010). Microbial production of silver nanoparticles. Dig. J. Nanomater. Biostruct..

[cit230] Valli J. S., Vaseeharan B. (2012). Biosynthesis of silver nanoparticles by Cissus quadrangularis extracts. Mater. Lett..

[cit231] Zhang X.-F., Liu Z.-G., Shen W., Gurunathan S. (2016). Silver nanoparticles: synthesis, characterization, properties, applications, and therapeutic approaches. Int. J. Mol. Sci..

[cit232] Leung A. B., Suh K. I., Ansari R. R. (2006). Particle-size and velocity measurements in flowing conditions using dynamic light scattering. Appl. Opt..

[cit233] Ritu V. K. K., Das A., Chandra P. (2023). Phytochemical-based synthesis of silver nanoparticle: mechanism and potential applications. J. Bionanoscience.

[cit234] Link S., El-Sayed M. A. (2003). Optical properties and ultrafast dynamics of metallic nanocrystals. Annu. Rev. Phys. Chem..

[cit235] Noginov M., Zhu G., Bahoura M., Adegoke J., Small C., Ritzo B. (2007). *et al.*, The effect of gain and absorption on surface plasmons in metal nanoparticles. Appl. Phys. B.

[cit236] Reda M., Ashames A., Edis Z., Bloukh S., Bhandare R., Abu Sara H. (2019). Green synthesis of potent antimicrobial silver nanoparticles using different plant extracts and their mixtures. Processes.

[cit237] Ajitha B., Reddy Y. A. K., Reddy P. S. (2014). Biogenic nano-scale silver particles by Tephrosia purpurea leaf extract and their inborn antimicrobial activity. Spectrochim. Acta, Part A.

[cit238] Ijaz I., Gilani E., Nazir A., Bukhari A. (2020). Detail review on chemical, physical and green synthesis, classification, characterizations and applications of nanoparticles. Green Chem. Lett. Rev..

[cit239] Anandalakshmi K., Venugobal J., Ramasamy V. (2016). Characterization of silver nanoparticles by green synthesis method using Pedalium murex leaf extract and their antibacterial activity. Appl. Nanosci..

[cit240] Lavoie D., Little B., Ray R., Bennett R., Lambert M., Asper V. (1995). *et al.*, Environmental scanning electron microscopy of marine aggregates. J. Microsc..

[cit241] Carter C. B. , and Williams D. B., Transmission Electron Microscopy: Diffraction, Imaging, and Spectrometry, Springer, 2016

[cit242] Asoro M. A., Kovar D., Ferreira P. J. (2013). In situ transmission electron microscopy observations of sublimation in silver nanoparticles. ACS Nano.

[cit243] Rajeshkumar S., Bharath L. (2017). Mechanism of plant-mediated synthesis of silver nanoparticles–a review on biomolecules involved, characterisation and antibacterial activity. Chem.-Biol. Interact..

[cit244] Otunola G. A., Afolayan A. J., Ajayi E. O., Odeyemi S. W. (2017). Characterization, antibacterial and antioxidant properties of silver nanoparticles synthesized from aqueous extracts of Allium sativum, *Zingiber officinale*, and Capsicum frutescens. Pharmacogn. Mag..

[cit245] Wadhwa P., Sharma S., Sahu S., Sharma A., Kumar D. (2022). A review of nanoparticles characterization techniques. Curr. Nanomater..

[cit246] Kumar S., Barth A. (2010). Following enzyme activity with infrared spectroscopy. Sensors.

[cit247] Cabral M., Pedrosa F., Margarido F., Nogueira C. (2013). End-of-life Zn–MnO2 batteries: electrode materials characterization. Environ. Technol..

[cit248] Dey A., Mukhopadhyay A. K., Gangadharan S., Sinha M. K., Basu D. (2009). Characterization of microplasma sprayed hydroxyapatite coating. J. Therm. Spray Technol..

[cit249] Varberg T. D., Skakuj K. (2015). X-ray diffraction of intermetallic compounds: A physical
chemistry laboratory experiment. J. Chem. Educ..

[cit250] Gan J., Lu X., Wu J., Xie S., Zhai T., Yu M. (2013). *et al.*, Oxygen vacancies promoting photoelectrochemical performance of In2O3 nanocubes. Sci. Rep..

[cit251] Khan M. M., Ansari S. A., Amal M. I., Lee J., Cho M. H. (2013). Highly visible light active Ag@ TiO 2 nanocomposites synthesized using an electrochemically active biofilm: a novel biogenic approach. Nanoscale.

[cit252] Wang H., Zhang L., Chen Z., Hu J., Li S., Wang Z. (2014). *et al.*, Semiconductor heterojunction photocatalysts: design, construction, and photocatalytic performances. Chem. Soc. Rev..

[cit253] Ansari S. A., Khan M. M., Ansari M. O., Cho M. H. (2015). Silver nanoparticles and defect-induced visible light photocatalytic and photoelectrochemical performance of Ag@*m*-TiO_2_ nanocomposite. Sol. Energy Mater. Sol. Cells.

[cit254] Osman A. I., Zhang Y., Farghali M., Rashwan A. K., Eltaweil A. S., Abd El-Monaem E. M. (2024). *et al.*, Synthesis of green nanoparticles for energy, biomedical, environmental, agricultural, and food applications: A review. Environ. Chem. Lett..

[cit255] Ankanna S., Prasad T., Elumalai E., Savithramma N. (2010). Production of biogenic silver nanoparticles using Boswellia ovalifoliolata stem bark. Dig. J. Nanomater. Biostruct..

[cit256] Prabhu S., Poulose E. K. (2012). Silver nanoparticles: mechanism of antimicrobial action, synthesis, medical applications, and toxicity effects. Int. Nano Lett..

[cit257] Salleh A., Naomi R., Utami N. D., Mohammad A. W., Mahmoudi E., Mustafa N. (2020). *et al.*, The potential of silver nanoparticles for antiviral and antibacterial applications: A mechanism of action. Nanomaterials.

[cit258] Du J., Hu Z., Yu Z., Li H., Pan J., Zhao D. (2019). *et al.*, Antibacterial activity of a novel Forsythia suspensa fruit mediated green silver nanoparticles against food-borne pathogens and mechanisms investigation. Mater. Sci. Eng., C.

[cit259] Cavassin E. D., de Figueiredo L. F. P., Otoch J. P., Seckler M. M., de Oliveira R. A., Franco F. F. (2015). *et al.*, Comparison of methods to detect the *in vitro* activity of silver nanoparticles (AgNP) against multidrug resistant bacteria. J. Nanobiotechnol..

[cit260] Yan X., He B., Liu L., Qu G., Shi J., Hu L. (2018). *et al.*, Antibacterial mechanism of silver nanoparticles in Pseudomonas aeruginosa: proteomics approach. Metallomics.

[cit261] Pandey J. K., Swarnkar R., Soumya K., Dwivedi P., Singh M. K., Sundaram S. (2014). *et al.*, Silver nanoparticles synthesized by pulsed laser ablation: as a potent antibacterial agent for human enteropathogenic Gram-positive and Gram-negative bacterial strains. Appl. Biochem. Biotechnol..

[cit262] Du H., Lo T.-M., Sitompul J., Chang M. W. (2012). Systems-level analysis of Escherichia coli response to silver nanoparticles: the roles of anaerobic respiration in microbial resistance. Biochem. Biophys. Res. Commun..

[cit263] Beer C., Foldbjerg R., Hayashi Y., Sutherland D. S., Autrup H. (2012). Toxicity of silver nanoparticles—nanoparticle or silver ion?. Toxicol. Lett..

[cit264] Cronholm P., Karlsson H. L., Hedberg J., Lowe T. A., Winnberg L., Elihn K. (2013). *et al.*, Intracellular uptake and toxicity of Ag and CuO nanoparticles: a comparison between nanoparticles and their corresponding metal ions. Small.

[cit265] Mansoor S., Zahoor I., Baba T. R., Padder S. A., Bhat Z., Koul A. M. (2021). *et al.*, Fabrication of silver nanoparticles against fungal pathogens. Front. Nanotechnol..

[cit266] Bhardwaj K., Dhanjal D. S., Sharma A., Nepovimova E., Kalia A., Thakur S. (2020). *et al.*, Conifer-derived metallic nanoparticles: Green synthesis and biological applications. Int. J. Mol. Sci..

[cit267] Sivakumar T. (2021). A modern review of silver nanoparticles mediated plant extracts and its potential bioapplications. Int. J. Botany Stud..

[cit268] Elumalai E. , Prasad T., Venkata Kambala V. K., Nagajyothi P., David E., Green Synthesis of Silver Nanoparticle Using Euphorbia hirta L and their Antifungal Activities, 2010

[cit269] Siddiqi K. S., Rashid M., Rahman A., Tajuddin H. A., Rehman S. (2018). Biogenic fabrication and characterization of silver nanoparticles using aqueous-ethanolic extract of lichen (Usnea longissima) and their antimicrobial activity. Biomater. Res..

[cit270] Latha M., Priyanka M., Rajasekar P., Manikandan R., Prabhu N. (2016). Biocompatibility and antibacterial activity of the Adathoda vasica Linn extract mediated silver nanoparticles. Microb. Pathog..

[cit271] Rao M. L., Savithramma N. (2012). Antimicrobial activity of silver nanoparticles synthesized by using stem extract of Svensonia hyderobadensis (Walp.) Mold â€“A rare medicinal plant. Res. Biotechnol..

[cit272] Senthilkumar S., Sivakumar T. (2014). Green tea (*Camellia sinensis*) mediated synthesis of zinc oxide (ZnO) nanoparticles and studies on their antimicrobial activities. Int. J. Pharmacol. Pharm. Sci..

[cit273] Senthilkumar S. R., Sivakumar T., Arulmozhi K. T., Mythili N. (2016). FT-IR Analysis and Correlation Studies on the Antioxidant Activity, Total Phenolics, and Total Flavonoids of Indian Commercial Teas (Camellia sinensis L.)—A Novel Approach. Int. J. Biosci. Nanosci..

[cit274] Govindaraju K., Tamilselvan S., Kiruthiga V., Singaravelu G. (2010). Biogenic silver nanoparticles by Solanum torvum and their promising antimicrobial activity. J. Biopestic..

[cit275] Agarwal P., Mehta A., Kachhwaha S., Kothari S. (2013). Green synthesis of silver nanoparticles and their activity against Mycobacterium tuberculosis. Adv. Sci., Eng. Med..

[cit276] Sivakumar T. (2019). Phytochemical screening and GC-MS analysis of bioactive compounds and biosynthesis of silver nanoparticles using sprout extracts of Vigna radiate L. and their antioxidant and antibacterial activity. Asian J. Pharm. Clin. Res..

[cit277] Vankar P. S., Shukla D. (2012). Biosynthesis of silver nanoparticles using lemon leaves extract and its application for antimicrobial finish on fabric. Appl. Nanosci..

[cit278] Loo Y. Y., Rukayadi Y., Nor-Khaizura M.-A.-R., Kuan C. H., Chieng B. W., Nishibuchi M. (2018). *et al.*, *In vitro* antimicrobial activity of green synthesized silver nanoparticles against selected Gram-negative foodborne pathogens. Front. Microbiol..

[cit279] Kumar P. V., Pammi S., Kollu P., Satyanarayana K., Shameem U. (2014). Green synthesis and characterization of silver nanoparticles using Boerhaavia diffusa plant extract and their anti bacterial activity. Ind. Crops Prod..

[cit280] Singh A., Jain D., Upadhyay M., Khandelwal N., Verma H. (2010). Green synthesis of silver nanoparticles using Argemone mexicana leaf extract and evaluation of their antimicrobial activities. Dig. J. Nanomater. Biostruct..

[cit281] Simon S., Sibuyi N. R. S., Fadaka A. O., Meyer S., Josephs J., Onani M. O. (2022). *et al.*, Biomedical applications of plant extract-synthesized silver nanoparticles. Biomedicines.

[cit282] Fatima M., Zaidi S., Amraiz D., Afzal F. (2016). *In vitro* antiviral activity of Cinnamomum cassia and its nanoparticles against H7N3 influenza a virus. J. Microbiol. Biotechnol..

[cit283] Sharma V., Kaushik S., Pandit P., Dhull D., Yadav J. P., Kaushik S. (2019). Green synthesis of silver nanoparticles from medicinal plants and evaluation of their antiviral potential against chikungunya virus. Appl. Microbiol. Biotechnol..

[cit284] Haggag E. G., Elshamy A. M., Rabeh M. A., Gabr N. M., Salem M., Youssif K. A. (2019). *et al.*, Antiviral potential of green synthesized silver nanoparticles of Lampranthus coccineus and Malephora lutea. Int. J. Nanomed..

[cit285] Bunghez I., Barbinta Patrascu M., Badea N., Doncea S., Popescu A., Ion R. (2012). Antioxidant silver nanoparticles green synthesized using ornamental plants. J. Optoelectron. Adv. Mater..

[cit286] Salari S., Bahabadi S. E., Samzadeh-Kermani A., Yosefzaei F. (2019). In-vitro evaluation of antioxidant and antibacterial potential of greensynthesized silver nanoparticles using Prosopis farcta fruit extract. Iran. J. Pharm. Res..

[cit287] Nagaich U., Gulati N., Chauhan S. (2016). Antioxidant and antibacterial potential of silver nanoparticles: biogenic synthesis utilizing apple extract. J. Pharm..

[cit288] Netala V. R., Bukke S., Domdi L., Soneya S., Reddy S G., Bethu M. S. (2018). *et al.*, Biogenesis of silver nanoparticles using leaf extract of Indigofera hirsuta L. and their potential biomedical applications (3-in-1 system). Artificial cells, nanomedicine, and biotechnology.

[cit289] Kharat S. N., Mendhulkar V. D. (2016). Synthesis, characterization and studies on antioxidant activity of silver nanoparticles using Elephantopus scaber leaf extract. Mater. Sci. Eng., C.

[cit290] Mitchell M. J., Billingsley M. M., Haley R. M., Wechsler M. E., Peppas N. A., Langer R. (2021). Engineering precision nanoparticles for drug delivery. Nat. Rev. Drug Discovery.

[cit291] Dhir R., Chauhan S., Subham P., Kumar S., Sharma P., Shidiki A. (2024). *et al.*, Plant-mediated synthesis of silver nanoparticles: unlocking their pharmacological potential–a comprehensive review. Front. Bioeng. Biotechnol..

[cit292] Patel R. R., Singh S. K., Singh M. (2023). Green synthesis of silver nanoparticles: methods, biological applications, delivery and toxicity. Mater. Adv..

[cit293] Ivanova N. , Gugleva V., Dobreva M., Pehlivanov I., Stefanov S., and Andonova V., Silver Nanoparticles as Multi-Functional Drug Delivery Systems, IntechOpen London, UK, 2018

[cit294] Khalid S., Hanif R. (2017). Green biosynthesis of silver nanoparticles conjugated to gefitinib as delivery vehicle. Int. J. Adv. Sci. Eng. Technol..

[cit295] Lee S. H., Jun B.-H. (2019). Silver nanoparticles: synthesis and application for nanomedicine. Int. J. Mol. Sci..

[cit296] Benyettou F., Rezgui R., Ravaux F., Jaber T., Blumer K., Jouiad M. (2015). *et al.*, Synthesis of silver nanoparticles for the dual delivery of doxorubicin and alendronate to cancer cells. J. Mater. Chem. B.

[cit297] Ding Q., Liu D., Guo D., Yang F., Pang X., Che R. (2017). *et al.*, Shape-controlled fabrication of magnetite silver hybrid nanoparticles with high performance magnetic hyperthermia. Biomaterials.

[cit298] Naveed M., Batool H., Rehman S. U., Javed A., Makhdoom S. I., Aziz T. (2022). *et al.*, Characterization and evaluation of the antioxidant, antidiabetic, anti-inflammatory, and cytotoxic activities of silver nanoparticles synthesized using Brachychiton populneus leaf extract. Processes.

[cit299] Agarwal H., Nakara A., Shanmugam V. K. (2019). Anti-inflammatory mechanism of various metal and metal oxide nanoparticles synthesized using plant extracts: A review. Biomed. Pharmacother..

[cit300] Akhter M. S., Rahman M. A., Ripon R., Mubarak M., Akter M., Mahbub S. (2024). *et al.*, A systematic review on green synthesis of silver nanoparticles using plants extract and their bio-medical applications. Heliyon.

[cit301] Naganthran A., Verasoundarapandian G., Khalid F. E., Masarudin M. J., Zulkharnain A., Nawawi N. M. (2022). *et al.*, Synthesis, characterization and biomedical application of silver nanoparticles. Materials.

[cit302] Garg S., Chandra A., Mazumder A., Mazumder R. (2014). Green synthesis of silver nanoparticles using Arnebia nobilis root extract and wound healing potential of its hydrogel. Asian J. Pharm..

[cit303] Rigo C., Ferroni L., Tocco I., Roman M., Munivrana I., Gardin C. (2013). *et al.*, Active silver nanoparticles for wound healing. Int. J. Mol. Sci..

[cit304] Ahmadi M., Adibhesami M. (2017). The effect of silver nanoparticles on wounds contaminated with Pseudomonas aeruginosa in mice: an experimental study. Iran. J. Pharm. Res..

[cit305] Xu L., Wang Y.-Y., Huang J., Chen C.-Y., Wang Z.-X., Xie H. (2020). Silver nanoparticles: Synthesis, medical applications and biosafety. Theranostics.

[cit306] Ahmed R. H., Mustafa D. E. (2020). Green synthesis of silver nanoparticles mediated by traditionally used medicinal plants in Sudan. Int. Nano Lett..

[cit307] Castro-Aceituno V., Ahn S., Simu S. Y., Singh P., Mathiyalagan R., Lee H. A. (2016). *et al.*, Anticancer activity of silver nanoparticles from Panax ginseng fresh leaves in human cancer cells. Biomed. Pharmacother..

[cit308] Khateef R., Khadri H., Almatroudi A., Alsuhaibani S. A., Mobeen S. A., Khan R. A. (2019). Potential in-vitro anti-breast cancer activity of green-synthesized silver nanoparticles preparation against human MCF-7 cell-lines. Adv. Nat. Sci.:Nanosci. Nanotechnol..

[cit309] Lin J., Huang Z., Wu H., Zhou W., Jin P., Wei P. (2014). *et al.*, Inhibition of autophagy enhances the anticancer activity of silver nanoparticles. Autophagy.

[cit310] Ratan Z. A., Haidere M. F., Nurunnabi M., Shahriar S. M., Ahammad A. S., Shim Y. Y. (2020). *et al.*, Green chemistry synthesis of silver nanoparticles and their potential anticancer effects. Cancers.

[cit311] Venkatadri B., Shanparvish E., Rameshkumar M., Arasu M. V., Al-Dhabi N. A., Ponnusamy V. K. (2020). *et al.*, Green synthesis of silver nanoparticles using aqueous rhizome extract of *Zingiber officinale* and *Curcuma longa*: In-vitro anti-cancer potential on human colon carcinoma HT-29 cells. Saudi J. Biol. Sci..

[cit312] Ramar M., Manikandan B., Marimuthu P. N., Raman T., Mahalingam A., Subramanian P. (2015). *et al.*, Synthesis of silver nanoparticles using Solanum trilobatum fruits extract and its antibacterial, cytotoxic activity against human breast cancer cell line MCF 7. Spectrochim. Acta, Part A.

[cit313] He Y., Du Z., Ma S., Cheng S., Jiang S., Liu Y. (2016). *et al.*, Biosynthesis, antibacterial activity
and anticancer effects against prostate cancer (PC-3) cells of silver nanoparticles using Dimocarpus Longan Lour. peel extract. Nanoscale Res. Lett..

[cit314] Sarkar S., Kotteeswaran V. (2018). Green synthesis of silver nanoparticles from aqueous leaf extract of Pomegranate (Punica granatum) and their anticancer activity on human cervical cancer cells. Adv. Nat. Sci.:Nanosci. Nanotechnol..

[cit315] Adebayo I. A., Arsad H., Gagman H. A., Ismail N. Z., Samian M. R. (2020). Inhibitory effect of eco-friendly naturally synthesized silver nanoparticles from the leaf extract of medicinal Detarium microcarpum plant on pancreatic and cervical cancer cells. Asian Pac. J. Cancer Prev..

[cit316] Baharara J., Namvar F., Mousavi M., Ramezani T., Mohamad R. (2014). Anti-angiogenesis effect of biogenic silver nanoparticles synthesized using saliva officinalis on chick chorioalantoic membrane (CAM). Molecules.

[cit317] Kathiravan V., Ravi S., Ashokkumar S. (2014). Synthesis of silver nanoparticles from Melia dubia leaf extract and their *in vitro* anticancer activity. Spectrochim. Acta, Part A.

[cit318] Devi J. , and Bhimba B., Anticancer Activity of Silver Nanoparticles Synthesized by the Seaweed Ulva lactuca *In vitro*, vol. 1, p. , p. 242, 10.4172/scientificreports, silver nitrate solution was added to the filtrate slowly under magnetic stirring conditions for even coating of silver and subjected to heating at 12 C for 10 min, The extract is used as reducing and stabilizing agent for 1mM of Silver nitrate This one pot green synthesis was the modified method followed by Vigneshwaran et al. (18). 2012

[cit319] Hemlata M. P. R., Singh A. P., Tejavath K. K. (2020). Biosynthesis of silver nanoparticles using Cucumis prophetarum aqueous leaf extract and their antibacterial and antiproliferative activity against cancer cell lines. ACS Omega.

[cit320] Venkatesan B., Subramanian V., Tumala A., Vellaichamy E. (2014). Rapid synthesis of biocompatible silver nanoparticles using aqueous extract of Rosa damascena petals and evaluation of their anticancer activity. Asian Pac. J. Trop. Med..

[cit321] Kanipandian N., Thirumurugan R. (2014). A feasible approach to phyto-mediated synthesis of silver nanoparticles using industrial crop Gossypium hirsutum (cotton) extract as stabilizing agent and assessment of its *in vitro* biomedical potential. Ind. Crops Prod..

[cit322] Venugopal K., Rather H., Rajagopal K., Shanthi M., Sheriff K., Illiyas M. (2017). *et al.*, Synthesis of silver nanoparticles (Ag NPs) for anticancer activities (MCF 7 breast and A549 lung cell lines) of the crude extract of Syzygium aromaticum. J. Photochem. Photobiol., B.

[cit323] Jeyaraj M., Rajesh M., Arun R., MubarakAli D., Sathishkumar G., Sivanandhan G. (2013). *et al.*, An investigation on the cytotoxicity and caspase-mediated apoptotic effect of biologically synthesized silver nanoparticles using Podophyllum hexandrum on human cervical carcinoma cells. Colloids Surf., B.

[cit324] Vijistella Bai G. (2014). Green Synthesis Of Silver Nanostructures Against Human Cancer Cell Lines And Certain Pathogens. Int. J. Pharm., Chem. Biol. Sci..

[cit325] Fadaka A. O., Meyer S., Ahmed O., Geerts G., Madiehe M. A., Meyer M. (2022). *et al.*, Broad spectrum anti-bacterial activity and non-selective toxicity of gum Arabic silver nanoparticles. Int. J. Mol. Sci..

[cit326] Firdhouse M. J., Lalitha P. (2015). Biosynthesis of silver nanoparticles and its applications. J. Nanotechnol..

[cit327] Priyadharshini R. I., Prasannaraj G., Geetha N., Venkatachalam P. (2014). Microwave-mediated extracellular synthesis of metallic silver and zinc oxide nanoparticles using macro-algae (Gracilaria edulis) extracts and its anticancer activity against human PC3 cell lines. Appl. Biochem. Biotechnol..

[cit328] Ahmed S., Ahmad M., Swami B. L., Ikram S. (2016). A review on plants extract mediated synthesis of silver nanoparticles for antimicrobial applications: a green expertise. J. Adv. Res..

[cit329] Yusuf M. , Silver nanoparticles: synthesis and applications, Handbook of Ecomaterials, 2019, p. 2343

[cit330] Liu F., Yang J., Zuo J., Ma D., Gan L., Xie B. (2014). *et al.*, Graphene-supported nanoscale zero-valent iron: removal of phosphorus from aqueous solution and mechanistic study. J. Environ. Sci..

[cit331] Tang W.-W., Zeng G.-M., Gong J.-L., Liang J., Xu P., Zhang C. (2014). *et al.*, Impact of humic/fulvic acid on the removal of heavy metals from aqueous solutions using nanomaterials: a review. Sci. Total Environ..

[cit332] Yan J., Han L., Gao W., Xue S., Chen M. (2015). Biochar supported nanoscale zerovalent iron composite used as persulfate activator for removing trichloroethylene. Bioresour. Technol..

[cit333] Kalhapure R. S., Sonawane S. J., Sikwal D. R., Jadhav M., Rambharose S., Mocktar C. (2015). *et al.*, Solid lipid nanoparticles of clotrimazole silver complex: an efficient nano antibacterial against Staphylococcus aureus and MRSA. Colloids Surf., B.

[cit334] Li X., Lenhart J. J., Walker H. W. (2012). Aggregation kinetics and dissolution of coated silver nanoparticles. Langmuir.

[cit335] Quang D. V., Sarawade P. B., Jeon S. J., Kim S. H., Kim J.-K., Chai Y. G. (2013). *et al.*, Effective water disinfection using silver nanoparticle containing silica beads. Appl. Surf. Sci..

[cit336] Dankovich T. A., Gray D. G. (2011). Bactericidal paper impregnated with silver nanoparticles for point-of-use water treatment. Environ. Sci. Technol..

[cit337] Ren D., Smith J. A. (2013). Retention and transport of silver nanoparticles in a ceramic porous medium used for point-of-use water treatment. Environ. Sci. Technol..

[cit338] Zahoor M., Nazir N., Iftikhar M., Naz S., Zekker I., Burlakovs J. (2021). *et al.*, A review on silver nanoparticles: Classification, various methods of synthesis, and their potential roles in biomedical applications and water treatment. Water.

[cit339] Abu-Saied M., Taha T. H., El-Deeb N. M., Hafez E. E. (2018). Polyvinyl alcohol/Sodium alginate integrated silver nanoparticles as probable solution for decontamination of microbes contaminated water. Int. J. Biol. Macromol..

[cit340] Dawadi S., Katuwal S., Gupta A., Lamichhane U., Thapa R., Jaisi S. (2021). *et al.*, Current research on silver nanoparticles: synthesis, characterization, and applications. J. Nanomater..

[cit341] Che W., Xiao Z., Wang Z., Li J., Wang H., Wang Y. (2019). *et al.*, Wood-based mesoporous filter decorated with silver nanoparticles for water purification. ACS Sustain. Chem. Eng..

[cit342] Cheon J. Y., Kim S. J., Park W. H. (2019). Facile interpretation of catalytic reaction between organic dye pollutants and silver nanoparticles with different shapes. J. Nanomater..

[cit343] Zhang Z., Shao C., Sun Y., Mu J., Zhang M., Zhang P. (2012). *et al.*, Tubular nanocomposite catalysts based on size-controlled and highly dispersed silver nanoparticles assembled on electrospun silica nanotubes for catalytic reduction of 4-nitrophenol. J. Mater. Chem..

[cit344] Samuel M. S., Jose S., Selvarajan E., Mathimani T., Pugazhendhi A. (2020). Biosynthesized silver nanoparticles using Bacillus amyloliquefaciens; Application for cytotoxicity effect on A549 cell line and photocatalytic degradation of p-nitrophenol. J. Photochem. Photobiol., B.

[cit345] Panáček A., Prucek R., Hrbáč J., Nevečná Tj, Šteffková J., Zbořil R. (2014). *et al.*, Polyacrylate-assisted size control of silver nanoparticles and their catalytic activity. Chem. Mater..

[cit346] Salehi-Khojin A., Jhong H.-R. M., Rosen B. A., Zhu W., Ma S., Kenis P. J. (2013). *et al.*, Nanoparticle silver catalysts that show enhanced activity for carbon dioxide electrolysis. J. Phys. Chem. C.

[cit347] Adhikary J., Meyerstein D., Marks V., Meistelman M., Gershinsky G., Burg A. (2018). *et al.*, Sol-gel entrapped Au0-and Ag0-nanoparticles catalyze reductive de-halogenation of halo-organic compounds by BH_4_^−^. Appl. Catal., B.

[cit348] Jaffri S. B., Ahmad K. S. (2020). Biomimetic detoxifier Prunus cerasifera Ehrh. silver nanoparticles: innate green bullets for morbific pathogens and persistent pollutants. Environ. Sci. Pollut. Res..

[cit349] Ziashahabi A., Prato M., Dang Z., Poursalehi R., Naseri N. (2019). The effect of silver oxidation on the photocatalytic activity of Ag/ZnO hybrid plasmonic/metal-oxide nanostructures under visible light and in the dark. Sci. Rep..

[cit350] Revathy R., Joseph J., Augustine C., Sajini T., Mathew B. (2022). Synthesis and catalytic applications of silver nanoparticles: a sustainable chemical approach using indigenous reducing and capping agents from Hyptis capitata. Environ. Sci.:Adv..

[cit351] Aslam M., Fozia F., Gul A., Ahmad I., Ullah R., Bari A. (2021). *et al.*, Phyto-extract-mediated synthesis of silver nanoparticles using aqueous extract of Sanvitalia procumbens, and characterization, optimization and photocatalytic degradation of azo dyes Orange G and Direct Blue-15. Molecules.

[cit352] Choi H.-J., Kim K. H. (2016). Parametric study of a dyeing wastewater treatment by modified sericite. Environ. Technol..

[cit353] Jin J., Zhao X., Du Y.-H., Ding M., Xiang C., Yan N. (2018). *et al.*, Nanostructured three-dimensional percolative channels for separation of oil-in-water emulsions. Iscience.

[cit354] Cao E., Duan W., Wang F., Wang A., Zheng Y. (2017). Natural cellulose fiber derived hollow-tubular-oriented polydopamine: in-situ formation of Ag nanoparticles for reduction of 4-nitrophenol. Carbohydr. Polym..

[cit355] Guo M., Zhang Y., Du F., Wu Y., Zhang Q., Jiang C. (2019). Silver nanoparticles/polydopamine coated polyvinyl alcohol sponge as an effective and recyclable catalyst for reduction of 4-nitrophenol. Mater. Chem. Phys..

[cit356] Melinte V., Stroea L., Buruiana T., Chibac A. L. (2019). Photocrosslinked hybrid composites with Ag, Au or Au-Ag NPs as visible-light triggered photocatalysts for degradation/reduction of aromatic nitroderivatives. Eur. Polym. J..

[cit357] Roy K., Sarkar C. K., Ghosh C. K. (2015). Rapid colorimetric detection of Hg2+ ion by green silver nanoparticles synthesized using Dahlia pinnata leaf extract. Green Process. Synth..

[cit358] Cheng D., Zhang Y., Liu Y., Bai X., Ran J., Bi S. (2020). *et al.*, Mussel-inspired synthesis of filter cotton-based AgNPs for oil/water separation, antibacterial and catalytic application. Mater. Today Commun..

[cit359] Rani P., Kumar V., Singh P. P., Matharu A. S., Zhang W., Kim K.-H. (2020). *et al.*, Highly stable AgNPs prepared via a novel green approach for catalytic and photocatalytic removal of biological and non-biological pollutants. Environ. Int..

[cit360] Hu D., Gao T., Kong X., Ma N., Fu J., Meng L. (2022). *et al.*, Ginger (*Zingiber officinale*) extract mediated green synthesis of silver nanoparticles and evaluation of their antioxidant activity and potential catalytic reduction activities with Direct Blue 15 or Direct Orange 26. PLoS One.

[cit361] Kalsoom U., Bhatti H. N., Aftab K., Amin F., Jesionowski T., Bilal M. (2023). Biocatalytic potential of *Brassica oleracea* L. var. botrytis leaves peroxidase for efficient degradation of textile dyes in aqueous medium. Bioprocess Biosyst. Eng..

[cit362] Chand K., Cao D., Fouad D. E., Shah A. H., Dayo A. Q., Zhu K. (2020). *et al.*, Green synthesis, characterization and photocatalytic application of silver nanoparticles synthesized by various plant extracts. Arabian J. Chem..

[cit363] Khac K. T., Phu H. H., Thi H. T., Do Thi H. (2023). Biosynthesis of silver nanoparticles using tea leaf extract (*Camellia sinensis*) for photocatalyst and antibacterial effect. Heliyon.

[cit364] Baran A., Keskin C., Baran M. F., Huseynova I., Khalilov R., Eftekhari A. (2021). *et al.*, Ecofriendly synthesis of silver nanoparticles using *Ananas comosus* fruit peels: anticancer and antimicrobial activities. Bioinorg. Chem. Appl..

[cit365] Hassaan M. A., El-Nemr M. A., Elkatory M. R., Ragab S., Niculescu V.-C., El Nemr A. (2023). Principles of photocatalysts and their different applications: a review. Top. Curr. Chem..

[cit366] Khabeeri O. M., Al-Thabaiti S. A., Khan Z. (2020). *Citrus sinensis* peel waste assisted synthesis of AgNPs: effect of surfactant on the nucleation and morphology. SN Appl. Sci..

[cit367] Characterization and anti-urolthiatic activity green synthesized of silver nanoparticles using the *Pandanus atrocarpus* extraction, AIP Conference Proceedings, ed. A. H. Jabbar, S. O. Mezan, S. M. A. Absi, A. T. Jabbar, A. S. Kareem, and F. Q. J. Al-Zayadi, AIP Publishing, 2022

[cit368] Rabeea M. A. , Owaid M. N., and Muslim R. F., Synthesis And Characterization Of Silver Nanoparticles By Natural Organic Compounds Extracted From Eucalyptus Leaves And Their Role In The Catalytic Degradation Of Methylene Blue Dye, 2021

[cit369] Kiran S., Albargi H. B., Afzal G., Aimun U., Anjum M. N., Qadir M. B. (2023). *et al.*, A zadirachta indica-assisted green synthesis of magnesium oxide nanoparticles for degradation of Reactive Red 195 dye: a sustainable environmental remedial approach. Appl. Water Sci..

[cit370] Neella N., Gaddam V., Nayak M., Dinesh N., Rajanna K. (2017). Scalable fabrication of highly sensitive flexible temperature sensors based on silver nanoparticles coated reduced graphene oxide nanocomposite thin films. Sens. Actuators, A.

[cit371] Maciel J. V., Durigon A. M. M., Souza M. M., Quadrado R. F., Fajardo A. R., Dias D. (2019). Polysaccharides derived from natural sources applied to the development of chemically modified electrodes for environmental applications: A review. Trends Environ. Anal. Chem..

[cit372] de Almeida L. S., Oreste E. Q., Maciel J. V., Heinemann M. G., Dias D. (2020). Electrochemical devices obtained from biochar: Advances in renewable and environmentally-friendly technologies applied to analytical chemistry. Trends Environ. Anal. Chem..

[cit373] Khan Z. U. H., Khan A., Chen Y., ullah Khan A., Shah N. S., Muhammad N. (2017). *et al.*, Photo catalytic applications of gold nanoparticles synthesized by green route and electrochemical degradation of phenolic Azo dyes using AuNPs/GC as modified paste electrode. J. Alloys Compd..

[cit374] Min S.-H., Lee G.-Y., Ahn S.-H. (2019). Direct printing of highly sensitive, stretchable, and durable strain sensor based on silver nanoparticles/multi-walled carbon nanotubes composites. Composites, Part B.

[cit375] Chiu C.-W., Li J.-W., Huang C.-Y., Yang S.-S., Soong Y.-C., Lin C.-L. (2020). *et al.*, Controlling the structures, flexibility, conductivity stability of three-dimensional conductive networks of silver nanoparticles/carbon-based nanomaterials with nanodispersion and their application in wearable electronic sensors. Nanomaterials.

[cit376] Pan T.-t, Sun D.-W., Pu H., Wei Q. (2018). Simple approach for the rapid detection of alternariol in pear fruit by surface-enhanced Raman scattering with pyridine-modified
silver nanoparticles. J. Agric. Food Chem..

[cit377] Yang L., Zhen S. J., Li Y. F., Huang C. Z. (2018). Silver nanoparticles deposited on graphene oxide for ultrasensitive surface-enhanced Raman scattering immunoassay of cancer biomarker. Nanoscale.

[cit378] Rastogi L., Sashidhar R., Karunasagar D., Arunachalam J. (2014). Gum kondagogu reduced/stabilized silver nanoparticles as direct colorimetric sensor for the sensitive detection of Hg2+ in aqueous system. Talanta.

[cit379] Karimi S., Samimi T. (2019). Green and simple synthesis route of Ag@ AgCl nanomaterial using green marine crude extract and its application for sensitive and selective determination of mercury. Spectrochim. Acta, Part A.

[cit380] Gao J., Jia M., Xu Y., Zheng J., Shao N., Zhao M. (2018). Prereduction-promoted enhanced growth of silver nanoparticles for ultrasensitive colorimetric detection of alkaline phosphatase and carbohydrate antigen 125. Talanta.

[cit381] Trang N. L. N., Nga D. T. N., Hoang V.-T., Ngo X.-D., Nhung P. T., Le A.-T. (2022). Bio-AgNPs-based electrochemical nanosensors for the sensitive determination of 4-nitrophenol in tomato samples: the roles of natural plant extracts in physicochemical parameters and sensing performance. RSC Adv..

[cit382] Jayaprakash M., Kannappan S. (2022). An overview of a sustainable approach to the biosynthesis of AgNPs for electrochemical sensors. Arabian J. Chem..

[cit383] Beck F., Loessl M., Baeumner A. J. (2023). Signaling strategies of silver nanoparticles in optical and electrochemical biosensors: Considering their potential for the point-of-care. Microchim. Acta.

[cit384] Hano C. , and Abbasi B. H., Plant-based Green Synthesis of Nanoparticles: Production, Characterization and Applications, MDPI, 2021, p. 31 10.3390/biom12010031 PMC8773616 35053179

[cit385] Ibrahim N. H., Taha G. M., Hagaggi N. S. A., Moghazy M. A. (2024). Green synthesis of silver nanoparticles and its environmental sensor ability to some heavy metals. BMC Chem..

[cit386] Alabdallah N. M., Hasan M. M. (2021). Plant-based green synthesis of silver nanoparticles and its effective role in abiotic stress tolerance in crop plants. Saudi J. Biol. Sci..

[cit387] Fares A., Mahdy A., Ahmed G. (2024). Unraveling the mysteries of silver nanoparticles: synthesis, characterization, antimicrobial effects and uptake translocation in plant—a review. Planta.

[cit388] Singh P., Kim Y.-J., Zhang D., Yang D.-C. (2016). Biological synthesis of nanoparticles from plants and microorganisms. Trends Biotechnol..

[cit389] Yan A., Chen Z. (2019). Impacts of silver nanoparticles on plants: a focus on the phytotoxicity and underlying mechanism. Int. J. Mol. Sci..

[cit390] Rodríguez-Félix F., Graciano-Verdugo A. Z., Moreno-Vásquez M. J., Lagarda-Díaz I., Barreras-Urbina C. G., Armenta-Villegas L. (2022). *et al.*, Trends in Sustainable Green Synthesis of Silver Nanoparticles Using Agri-Food Waste Extracts and Their Applications in Health. J. Nanomater..

[cit391] Manosalva N., Tortella G., Cristina Diez M., Schalchli H., Seabra A. B., Durán N. (2019). *et al.*, Green synthesis of silver nanoparticles: effect of synthesis reaction parameters on antimicrobial activity. World J. Microbiol. Biotechnol..

[cit392] Mohammadlou M., Maghsoudi H., Jafarizadeh-Malmiri H. (2016). A review on green silver nanoparticles based on plants: Synthesis, potential applications and eco-friendly approach. Int. Food Res. J..

[cit393] Hosseingholian A., Gohari S. D., Feirahi F., Moammeri F., Mesbahian G., Moghaddam Z. S. (2023). *et al.*, Recent advances in green synthesized nanoparticles: From production to application. Mater. Today Sustain..

[cit394] Mbatha L. S., Akinyelu J., Chukwuma C. I., Mokoena M. P., Kudanga T. (2023). Current trends and prospects for application of green synthesized metal nanoparticles in cancer and COVID-19 therapies. Viruses.

[cit395] Chung I.-M., Park I., Seung-Hyun K., Thiruvengadam M., Rajakumar G. (2016). Plant-mediated synthesis of silver nanoparticles: their characteristic properties and therapeutic applications. Nanoscale Res. Lett..

[cit396] Tariq M., Mohammad K. N., Ahmed B., Siddiqui M. A., Lee J. (2022). Biological synthesis of silver nanoparticles and prospects in plant disease management. Molecules.

[cit397] Reidy B., Haase A., Luch A., Dawson K. A., Lynch I. (2013). Mechanisms of silver nanoparticle release, transformation and toxicity: a critical review of current knowledge and recommendations for future studies and applications. Materials.

[cit398] Vanlalveni C., Lallianrawna S., Biswas A., Selvaraj M., Changmai B., Rokhum S. L. (2021). Green synthesis of silver nanoparticles using plant extracts and their antimicrobial activities: A review of recent literature. RSC Adv..

[cit399] Husain S., Nandi A., Simnani F. Z., Saha U., Ghosh A., Sinha A. (2023). *et al.*, Emerging trends in advanced translational applications of silver nanoparticles: a progressing dawn of nanotechnology. J. Funct. Biomater..

[cit400] Ying S., Guan Z., Ofoegbu P. C., Clubb P., Rico C., He F. (2022). *et al.*, Green synthesis of nanoparticles: Current developments and limitations. Environ. Technol. Innovation.

[cit401] Banerjee P., Satapathy M., Mukhopahayay A., Das P. (2014). Leaf extract mediated green synthesis of silver nanoparticles from widely available Indian plants: synthesis, characterization, antimicrobial property and toxicity analysis. Bioresour. Bioprocess..

[cit402] Srikar S. K., Giri D. D., Pal D. B., Mishra P. K., Upadhyay S. N. (2016). Green synthesis of silver nanoparticles: a review. Green Sustainable Chem..

[cit403] Giri A. K., Jena B., Biswal B., Pradhan A. K., Arakha M., Acharya S. (2022). *et al.*, Green synthesis and characterization of silver nanoparticles using Eugenia roxburghii DC. extract and activity against biofilm-producing bacteria. Sci. Rep..

[cit404] Ejaz U., Afzal M., Mazhar M., Riaz M., Ahmed N., Rizg W. Y. (2024). *et al.*, Characterization, synthesis, and biological activities of silver nanoparticles produced via green synthesis method using Thymus vulgaris aqueous extract. Int. J. Nanomed..

[cit405] Plaskova A., Mlcek J. (2023). New insights of the application of water or ethanol-water plant extract rich in active compounds in food. Front. Nutr..

[cit406] Gallina L., Cravotto C., Capaldi G., Grillo G., Cravotto G. (2022). Plant extraction in water: Towards highly efficient industrial applications. Processes.

